# Electrochemical Three‐Component Synthesis of Vinyl Sulfonamides via Decarboxylative Sulfonylation of Cinnamic Acids

**DOI:** 10.1002/cssc.202501920

**Published:** 2025-12-19

**Authors:** Po‐Chung Chien, Harald Kelm, Georg Manolikakes

**Affiliations:** ^1^ Department of Chemistry RPTU University Kaiserslautern‐Landau Kaiserslautern Germany

**Keywords:** decarboxylative functionalization, electrochemistry, multicomponent reactions, sulfonamide, sulfur dioxide

## Abstract

An efficient, electrochemical three‐component reaction for the synthesis of vinyl sulfonamides from cinnamic acids, SO_2_, and amines is reported. This metal‐free protocol utilizes inexpensive graphite electrodes and easy‐to‐use SO_2_ stock solutions to facilitate a decarboxylative transformation under mild conditions. The reaction proceeds with high regio‐ and stereoselectivity. The use of cinnamic acid derivatives as biobased feedstocks, combined with the demonstrated scalability and electrode/electrolyte reusability, highlights the potential of this approach for a sustainable synthesis of the important vinyl sulfonamide scaffold.

## Graphical abstract

An efficient, metal‐free electrochemical three‐component reaction of cinnamic acids, SO_2_, and amines to access (*E*)‐*β*‐styryl sulfonamides is reported. Utilizing graphite electrodes and SO_2_ stock solutions, this decarboxylative protocol proceeds under mild conditions with high regio‐ and stereoselectivity. Scalability, electrode/electrolyte reusability, and renewable feedstocks underscore its sustainable synthetic potential.

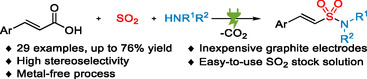

## 1 Introduction

**FIGURE 1:**
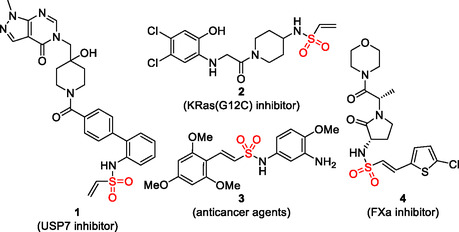
Representative examples of medically relevant vinyl sulfonamides.

Sulfonamides, characterized by the SO_2_–N functional group, are important compounds in medicinal and industrial chemistry [1, 2, 3, 4, 5]. As such, the development of efficient and sustainable methods for sulfonamide synthesis remains a significant goal in synthetic chemistry. One promising strategy involves the direct incorporation of sulfur dioxide (SO_2_) into organic molecules, enabling a modular and efficient synthesis of diverse sulfonamides with an increased step economy [6, 7, 8]. Potassium metabisulfite (K_2_S_2_O_5_) and DABSO (1,4‐diazabicyclo [2.2.2]octane bis(sulfur dioxide) adduct) [9, 10] have been established as convenient and easy‐to‐handle SO_2_ surrogates for this purpose [7, 8]. Recent advances also include the use of SO_2_ stock solutions, which offer a practical and accessible way to handle SO_2_ in laboratory settings [11]. As a result, efficient protocols for sulfonamide synthesis using both SO_2_ surrogates and SO_2_ stock solutions have been increasingly developed and optimized [12]. Among the various sulfonamide scaffolds, vinyl sulfonamides are of particular interest due to their electrophilic olefin moiety. Vinyl sulfonamides can be found in different covalent inhibitors, such as the Kras inhibitor or deubiquitinase USP7 inhibitor, both promising leads for cancer therapies, shown in Figure 1 [13, 14, 15]. Furthermore, vinyl sulfonamides have shown promising potential in drug discovery. In nude mouse xenograft assays, the compound (*E*)‐*N*‐(3‐amino‐4‐methoxyphenyl)‐2‐(2′, 4′, 6′‐trimethoxyphenyl)ethenesulfonamide (**3**) produced a marked reduction in tumor size, demonstrating its in vivo anticancer activity. In addition, GW813893 (**4**) has been identified as a potent, selective, and orally active factor Xa (FXa) inhibitor, providing key preclinical evidence supporting its potential as an oral antithrombotic agent (Figure 1) [16, 17, 18, 19]. Overall, vinyl sulfonamides have emerged as powerful tools in medicinal chemistry and drug development.

**SCHEME 1:**
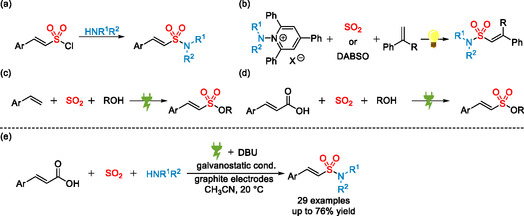
Representative approaches for the synthesis of vinyl sulfonates and vinyl sulfonamides. (a) Typical methods. (b) Photochemical sysnthesis of vinyl sulfonamides. (c) Electrochemical sysnthesis of vinyl sulfonates. (d) Electrochemical decarboxylative synthesis of vinyl sulfonates. (e) This work.

Despite the wide‐ranging applications of vinyl sulfonamides, their synthesis remains relatively underexplored, motivating our interest in developing new synthetic strategies. Established methods for vinyl sulfonamide synthesis typically rely on vinyl sulfonyl chlorides as key intermediates (Scheme 1a) [20, 21]. The common approach for the synthesis of vinyl sulfonyl chlorides is the addition of sulfuryl chloride, a harsh reagent, to olefins [22, 23]. Therefore, this route requires an additional step for the introduction of the sulfonyl functionality and is limited due to the incompatibility of sulfuryl chloride with sensitive functional groups. In 2024, our group reported a photochemical strategy for the synthesis of vinyl sulfonamides via the direct incorporation of SO_2_ (Scheme 1b) [24, 25]. This transformation offers a complementary route involving the in situ generation of sulfamoyl radicals as key intermediates for the introduction of the sulfonamide functionality. Recently, electrochemical strategies for the synthesis of sulfonates [26, 27, 28, 29, 30, 31, 32, 33, 34], sulfonamides [32, 35, 36], and sulfamides [37] via direct SO_2_ incorporation have emerged as efficient and modular methods for assembling sulfonyl‐functionalized scaffolds in a more sustainable manner [6, 7, 38, 39]. Notably, in 2024, the Waldvogel group reported an electrochemical multicomponent synthesis of vinyl sulfonates from styrenes, alcohols, and SO_2_, enabling direct formation of the sulfonate functionality from simple starting materials (Scheme 1c) [30]. In 2025, our group disclosed an electrochemical three‐component protocol for the synthesis of vinyl sulfonates via the decarboxylation of cinnamic acids with SO_2_ incorporation (Scheme 1d) [40]. This approach enables the direct valorization of cinnamic acids, as renewable, bio‐based feedstocks, into value‐added olefin sulfonates [41, 42].

Overall, electrochemical incorporation of sulfur dioxide avoids otherwise necessary pre‐functionalization of the substrates and redox reagents, which in turn leads to a minimized amount of reagent waste, thus simplifies the work‐up, costs, and sustainability of the overall process.

Building on this strategy, we now report a so far unprecedented electrochemical three‐component reaction for the synthesis of vinyl sulfonamides, based on the decarboxylative incorporation of SO_2_ into cinnamic acids (Scheme 1e). The reaction uses simple‐to‐handle SO_2_ stock solutions and inexpensive graphite electrodes, affording *β*‐styryl sulfonamides directly from potentially bio‐based feedstock streams with CO_2_ and H_2_ as the only by‐products.

## 2 Results and Discussion

### 2.1 Optimization of the Reaction Conditions

Optimization studies for the synthesis of vinyl sulfonamide **7a** using cinnamic acid (**5a**), an SO_2_ stock solution (4.6 M in MeCN, see SI for further details), and morpholine (**6a**) in the presence of a base, an anode electrode, and supporting electrolytes are summarized in Table 1. In the chosen electrolysis setup, SO_2_ is expected to be reduced at the cathode to form the radical anion, which can subsequently dimerize to dithionite or form complex ions with additional SO_2_ molecules [26, 35]. Therefore, a divided cell is preferred over an undivided one to prevent anodic oxidation of these species. In this divided cell setup, the catholyte contained supporting electrolytes and 5.0 equiv. (equivalents) of AcOH as a proton source for hydrogen evolution [43, 44, 45], utilizing the same electrode materials as in the anodic reaction. The best yield was obtained with inexpensive graphite electrodes, *n*Bu_4_NPF_6_ (0.1 M) as supporting electrolyte, 1,8‐diazabicyclo [5.4.0]undec‐7‐ene (DBU) as base, a current density of 15 mA/cm^2^, and an applied charge of 3.5 *F*, affording the vinyl sulfonamide **7a** in 73% isolated yield (Entry 1). The use of other electrodes commonly used in electrochemical SO_2_ fixation [29, 30, 31, 32, 35, 36, 37] furnished product **7a** in lower yields (Entries 2–4). Common cathode materials used in electrochemical cross‐dehydrogenative coupling (CDC) reactions, such as platinum, afforded the product in lower yield (Entry 5). Other bases such as 2,6‐lutidine, pyridine, and 1,5‐diazabicyclo [4.3.0]non‐5‐ene (DBN) led to decreased yields of the sulfonamide product (Entries 6–8). No product was observed with simple amine bases, such as *N*,*N*‐diisopropylethylamine (DIPEA), presumably due to facile anodic oxidation of the amine base (Entry 9). Varying the current density (10 or 20 mA/cm^2^) or the applied amount of charge (3.0 or 3.8 *F*) led to decreased yields of 55–65% (Entries 10–13). Decreasing or increasing the amount of morpholine, base, SO_2_, or the concentration of supporting electrolyte resulted in reduced yields of product **7a** (Entries 14–21). Replacing the supporting electrolyte *n*Bu_4_NPF_6_ with *n*Bu_4_NBF_4_ resulted in a lower yield (Entry 22). Only trace amounts of products were observed in the absence of a base (Entry 23). Finally, no reaction took place without applying an electric current (Entry 24). Notably, a stereoselective formation of the *E*‐vinyl sulfonamide was observed in all cases (*E*:*Z* > 20:1) [46].

**TABLE 1:** Influence of different parameters on the reaction outcome.

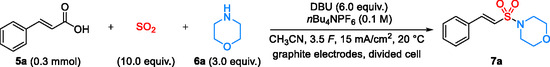					
Entry	Deviation from the standard conditions	Yield, %^a^	Entry	Deviation from the standard conditions	Yield, %^a^
**1**	**None**	**76 (73)** b	13	3.8 *F*	63
2	BDD electrodes	0	14	Morpholine (2.0 equiv.)	51
3	Glassy carbon electrodes	10	15	Morpholine (4.0 equiv.)	54
4	Pt foil electrodes	9	16	DBU (4.0 equiv.)	44
5	Graphite anode; Pt foil cathode	51	17	DBU (8.0 equiv.)	59
6	2,6‐Lutidine	31	18	SO_2_ (7.5 equiv.)	56
7	Pyridine	13	19	SO_2_ (12.5 equiv.)	73
8	DBN	51	20	*n*Bu_4_NPF_6_ (0.05 M)	57
9	DIPEA	0	21	*n*Bu_4_NPF_6_ (0.2 M)	58
10	10 mA/cm^2^	65	22	*n*Bu_4_NBF_4_ (0.1 M)	56
11	20 mA/cm^2^	55	23	No base	Traces
12	3.0 *F*	60	24	No electric current	0

a 1H NMR yield with the use of CHPh_3_ as the internal standard.

b Isolated yield.

### 2.2 Scope of the Reaction

**SCHEME 2:**
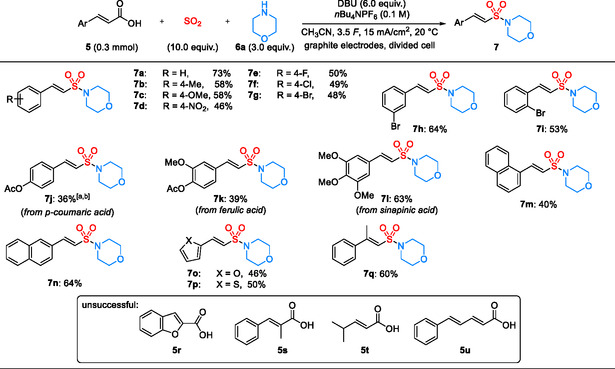
Scope of cinnamic acids and related substrates for the synthesis of vinyl sulfonamides **7**. Isolated yields are shown. (a) The applied charge of 3.0 *F* was used. (b) Under the original condition, 25% of **7j** was obtained.

Using the optimized conditions, the reaction of different cinnamic acid derivatives **5** with morpholine **6a** produced the corresponding products **7a**–**7q** in 36–73% (Scheme 2). Reactions with electron‐rich substrates afforded the corresponding vinyl sulfonamides **7b** and **7c** in slightly higher yields (58%) compared to reactions with electron‐poor substrates (**7d** or **7e**; 46% and 50%). Halogenated cinnamic acids **5e**–**5i** were well tolerated, furnishing the desired products **7e**–**7i** in 48–64% yield, without a significant influence of the substitution pattern. Hydroxy cinnamic acids, such as ferulic acid or sinapinic acid, represent an interesting class of biobased feedstock for these three‐component transformations. In order to avoid oxidative degradation of the phenolic core [43, 44, 45], reactions with the readily accessible acetylated derivatives **5j** (from *p*‐coumaric acid), **5k** (from ferulic acid), and **5l** (from sinapinic acid) were performed, furnishing the corresponding products **7j**–**7l** in 36–63%. A reduced applied charge of 3.0 *F* had to be used in the synthesis of **7j** to minimize product degradation under the reaction conditions. Overall, these reactions demonstrate that our process can provide a valuable approach to structurally interesting vinyl sulfonamides from hydroxycinnamic acid–based feedstocks, which are readily derived from lignocellulosic biomass [41, 42].

Reactions of the two naphthyl derivatives **5m** and **5n** furnished the corresponding sulfonamides **7m** and **7n** in 40% and 64% yield. The two heterocyclic acid derivatives **5o** and **5p** underwent decarboxylative SO_2_ insertion, delivering the sulfonamide products **7o** and **7p** in 46% and 50% yield. In contrast to our previous work on vinyl sulfonate synthesis [40], no additional functionalization of the aromatic core was observed in these cases. The reaction of *β*‐methylcinnamic acid **5q** afforded the trisubstituted vinyl sulfonamide **7q** in 60% yield. Importantly, a highly stereoselective formation of the *E*‐configured products was observed in every case (*E*:*Z* > 20:1). Unfortunately, all attempts to convert benzofuran‐2‐carboxylic acid **5r**, *α*‐methylcinnamic acids **5s**, simple acrylic acid derivatives **5t**, and conjugated dienoic acids **5u**, failed under our standard reaction conditions.

**SCHEME 3:**
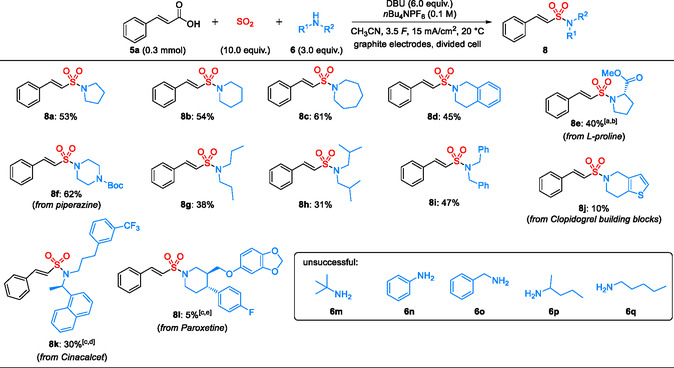
Scope of amines for the synthesis of vinyl sulfonamides **8**. Isolated yields are shown. (a) DBU (9.0 equiv.) was used. (b) Under the original condition, 16% of **8e** was obtained. (c) 0.3 mmol of amine and 2.0 equiv. of cinnamic acid were used. (d) Under the original condition, 19% of **8k** was obtained. (e) Under the original condition, 13% of **8l** was obtained.

Next, reactions of cinnamic acid **5a** with different amines **6** were investigated using the optimized conditions (Scheme 3). Both cyclic and acyclic secondary amines were tolerated, affording the desired vinyl sulfonamides **8a**–**8l**. Moderate to good yields of 45–61% were obtained with pyrrolidine, piperidine, azepane, and tetrahydroisoquinoline as cyclic secondary amines (compounds **8a**–**8d**). Sulfonamide **8e** was obtained in 40% yield using l‐proline methyl ester hydrochloride as the amine with an increased amount (9.0 equiv.) of DBU. Compound **8f** was obtained in 62% yield using Boc‐piperazine as an amine component. Three‐component reactions with acyclic secondary amines afforded the vinyl sulfonamides **8g**–**8i** in slightly lower yields (31–47%). Notably, the reaction of tetrahydrothieno[3,2‐*c*]pyridine, a building block in the synthesis of antiplatelet drug clopidogrel, afforded the product **8j** in 10% yield. As before, all products were formed with excellent diastereoselectivity (*E*:*Z* > 20:1). Reactions with drugs containing structurally complex amines, such as cinacalcet and paroxetine, proceeded less efficiently, affording sulfonamides **8k** and **8l** in 30% and 5% yield, respectively. These reactions show that the developed process is feasible for a three‐component diversification of drug‐like or API‐based amines. The controlled installation of an electrophilic vinyl sulfonamide warhead can serve as a starting point for the efficient construction of covalent drugs from already established drug‐like scaffolds. The lower yields with tetrahydrothieno[3,2‐*c*]pyridine and paroxetine presumably arise from competitive oxidative degradation of the amine **6j** and **6l** or sulfamide formation for cinacalcet (see Supporting Information for details) [37, 43, 44, 45]. Although yields for the diversification of the selected APIs and drug‐like scaffolds are low so far, our method provides fast access to the new drug‐like scaffolds containing a covalent vinyl sulfonamide scaffold for initial biological testing. Furthermore, optimization for individual substrate combinations can lead to higher‐yielding processes.

While secondary amines afforded the desired products, all reactions with primary amines **6m**–**6q** were unsuccessful so far. So far, we have no conclusive explanation for the failure of primary amines. However, we assume that the formation of the key amidosulfinate intermediate is less efficient, and a competing oxidative degradation of the cinnamic acid takes place.

### 2.3 Scale‐Up and Reusability Tests

**SCHEME 4:**
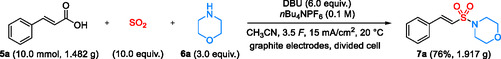
Scale‐up experiment.

A 33‐fold scale‐up experiment was performed to demonstrate the applicability of this protocol (Scheme 4). The gram‐scale reaction of **5a** with **6a** provided vinyl sulfonamide **7a** in 76% yield, with a similar result to that in the small‐scale reaction (73%; equals 43% current efficiency (CE); theoretical maximum CE (for 3.5 *F*) =  57%).

**FIGURE 2:**
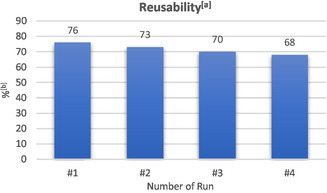
Reusability tests. (a) Anolyte: **5a** (0.3 mmol, 0.1 M), SO_2_ (10.0 equiv.), **6a** (3.0 equiv.), DBU (6.0 equiv.), *n*Bu_4_NPF_6_ (0.1 M), CH_3_CN, divided cell (glass frit), graphite electrodes, 15 mA/cm^2^, 3.5 *F*, 20°C. Catholyte: *n*Bu_4_NPF_6_ (0.1 M), AcOH (5.0 equiv.), CH_3_CN. (b) ^1^H NMR yield with the use of CHPh_3_ as the internal standard.

Additionally, we evaluated the reusability of the electrolysis setup by performing the reaction under identical conditions across four consecutive runs (Figure 2). In each cycle, only the anolyte was replaced, while the catholyte and electrodes were reused without further treatment except addition of additional AcOH before each run (see Supporting Information for details). These experiments showed a slight drop in yield from 76% to 68% over four runs. Compared to our previous work on vinyl sulfonate synthesis [40], only minor electrode fouling and no deposition of a polymer layer on the anode were observed. As demonstrated by the Waldvogel group, electrolyte mixtures containing alcohol, amine, and SO_2_ can be readily recycled [36]. Recycling of both the anolyte and catholyte mixture can lead to highly sustainable processes for the synthesis of medicinally relevant vinyl sulfonamides. Moreover, the possible option to evaporate anolyte components facilitates downstream processing in large‐scale electrolysis, marking a key step toward practical implementation [47]. Together, the scale‐up and reusability tests highlight the potential for the electrification of technical organic syntheses.

### 2.4 Mechanistic Studies

**SCHEME 5:**
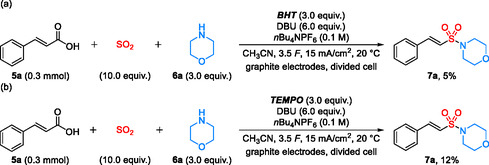
Control experiments.

**SCHEME 6:**
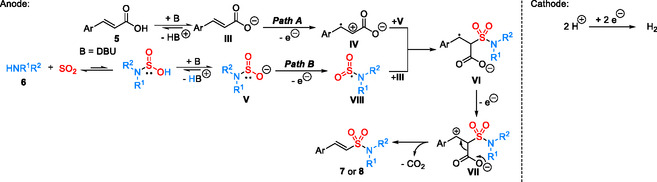
Proposed reaction pathway.

Control experiments and cyclic voltammetry studies were conducted to elucidate the reaction mechanism. The addition of radical scavengers such as 2,6‐di‐*tert*‐butyl‐4‐methylphenol (BHT) (Scheme 5a) or 2,2,6,6‐tetramethylpiperidinyl‐oxyl (TEMPO) (Scheme 5b) significantly suppressed product formation as well as conversion of the cinnamic acid **5a**. However, no direct trapping products derived from the radical scavengers were observed.

**FIGURE 3:**
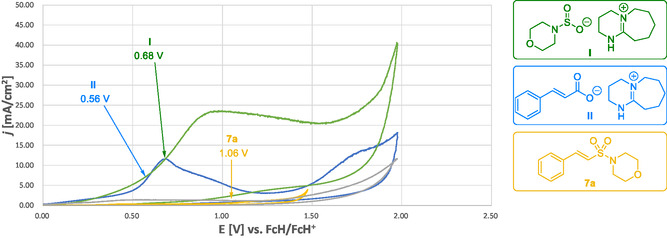
Cyclic voltammograms: cinnamate **II** (blue), amidosulfinate intermediate **I** (green), vinyl sulfonamide **7a** (yellow), and blank measurement (0.1 M *n*Bu_4_NPF_6_ in MeCN; grey). The numbers given refer to the half‐wave oxidation potential of the respective compounds or mixtures.

Cyclic voltammetry studies show that under standard conditions (excess base), the cinnamate salt **II** undergoes oxidation at a half‐wave potential of 0.56 V before the amidosulfinate **I** or overoxidation of the vinyl sulfonamide **7a** (Figure 3; see Supporting Information Figures S8–S12 for cyclic voltammograms for all individual components and mixtures).

Based on these results and literature precedents [48, 49, 50, 51, 52], we propose that a pseudo‐Kolbe‐type reaction pathway involving oxidation of the carbon scaffold is operative, rather than a classical Kolbe‐type direct oxidation of the carboxylic acid (Scheme 6). Initially, the cinnamic acid is deprotonated to form the corresponding carboxylate **III**. Subsequent one‐electron oxidation of **III** generates radical cation **IV**. The amidosulfinate species **V** is formed in situ from amine **6** and SO_2_, with DBU facilitating the equilibrium shift toward the deprotonated form [35, 36, 37]. Regioselective addition of **V** to **IV** yields the stabilized benzylic radical **VI** (Path A). A second anodic oxidation of **VI** produces the benzylic cation **VII**, which undergoes decarboxylation to afford the final products **7** and **8**. Although the reaction mechanism can be rationalized thermodynamically based on the oxidation potentials obtained from cyclic voltammetry, the kinetic perspective should also be considered (Path B). Specifically, the amidosulfinate species **V** is oxidized first to generate the S‐centered radical intermediate **VIII**, preceding oxidation of the carboxylate **III**. Regioselective addition of **VIII** to **III** then affords the intermediate **VI**, which undergoes subsequent oxidation and decarboxylation to yield the final products. The stereoselective formation of the *E*‐isomer likely proceeds via the most stable staggered conformation under kinetic control. On the cathodic side, hydrogen evolution from acetic acid serves as a simple and efficient counter‐reaction.

## 3 Conclusion

In summary, an electrochemical three‐component reaction for the synthesis of vinyl sulfonamides from cinnamic acids, amines, and a SO_2_ stock solution, using inexpensive graphite electrodes, is described. The method demonstrates a broad substrate scope (29 examples, up to 76% yield) and is compatible with structurally complex amines, enabling a late‐stage functionalization of drug‐like molecules. Mechanistic studies provide evidence for a pseudo‐Kolbe‐type decarboxylation pathway, enabling regioselective access to vinyl sulfonamides. Hence, this method extends the scope of still less‐explored pseudo‐Kolbe‐type decarboxylations to novel scaffolds and functionalities. The potential applicability of this process in technical organic syntheses was demonstrated with scale‐up experiments and electrode/electrolyte reusability studies. Furthermore, the direct conversion of biomass‐derived cinnamic acid opens an intriguing approach for the sustainable synthesis of vinyl sulfonamides for medical or materials applications. Studies to expand the applicability of decarboxylative SO_2_‐insertion reactions to other scaffolds are currently ongoing in our laboratories.

## Notes

An initial version of the manuscript was deposited to the ChemRxiv repository prior to submission [53].

## Supporting Information

Additional supporting information can be found online in the Supporting Information section. Supporting Information File 1: Experimental details, spectral and crystal data, DOIS, and copies of NMR spectra for all compounds prepared in this study. X‐Ray Data: cif and checkcif files for compound 7a (CCDC 2472612). The authors have cited additional references within the Supporting Information [55, 56, 57]. **Supporting Fig. S1:** IKA ElectraSyn 2.0 and IKA Carousell. **Supporting Fig. S2:** IKA Pro‐Divide divided cell. **Supporting Fig. S3**: Rohde & Schwarz HMP4040 power source. **Supporting Fig. S4:** Electrochemical glass cell used for the scale‐up experiment equipped with a P4 frit, graphite electrodes, caps, silicone septa, and electrode holders. Scale is given in cm. **Supporting Fig. S5:** Scale‐up experiment in a 2 x 100 mL glass cell before (left) and after (right) the electrolysis. **Supporting Fig. S6:** Tests for reusing the electrodes, glass frit membrane, and catholyte. ^[a]^Conditions described above. ^[b]1^H‐NMR yield with the use of CHPh_3_ as the internal standard. **Supporting Fig. S7:** Unsuccessful derivatives under the standard conditions. **Supporting Fig. S8:** Cyclic voltammograms of cinnamate **II** (blue), cinnamic acid **5a** (blue), morpholine **6a** (dark blue), amidosulfinate intermediate **I** (green), DBU (brown), vinyl sulfonamide **7a** (yellow), and blank measurement (0.1 M *n*Bu_4_NPF_6_ in CH_3_CN; grey). **Supporting Fig. S9:** Cyclic voltammograms of cinnamate **II** (blue), cinnamic acid **5a** (blue), DBU (brown), and blank measurement (0.1 M *n*Bu_4_NPF_6_ in CH_3_CN; grey). **Supporting Fig. S10:** Cyclic voltammograms of morpholine **6a** (dark blue), amidosulfinate intermediate **I** (green), DBU (brown), and blank measurement (0.1 M *n*Bu_4_NPF_6_ in CH_3_CN; grey). **Supporting Fig. S11:** Cyclic voltammograms of cinnamate **II**. **Supporting Fig. S12:** Cyclic voltammograms of (*E*)‐4‐(styrylsulfonyl)morpholine **7a**. **Supporting Fig. S13:** ^1^H (CDCl_3_, 400 MHz) and ^13^C{^1^H} (CDCl_3_, 101 MHz) NMR Spectrum of **7a**. **Supporting Fig. S14:** ^1^H (CDCl_3_, 400 MHz) and ^13^C{^1^H} (CDCl_3_, 101 MHz) NMR Spectrum of **7b**. **Supporting Fig. S15:** ^1^H (CDCl_3_, 400 MHz) and ^13^C{^1^H} (CDCl_3_, 101 MHz) NMR Spectrum of **7c**. **Supporting Fig. S16:** ^1^H (DMSO‐*d*_6_, 400 MHz) and ^13^C{^1^H} (DMSO‐*d*_6_, 101 MHz) NMR Spectrum of **7d**. **Supporting Fig. S17:** ^1^H (CDCl_3_, 400 MHz), ^13^C{^1^H} (CDCl_3_, 101 MHz), and ^19^F{^1^H} (CDCl_3_, 376 MHz) NMR Spectrum of **7e**. **Supporting Fig. S18:** ^1^H (CDCl_3_, 400 MHz) and ^13^C{^1^H} (CDCl_3_, 101 MHz) NMR Spectrum of **7f**. **Supporting Fig. S19:** ^1^H (CDCl_3_, 400 MHz) and ^13^C{^1^H} (CDCl_3_, 101 MHz) NMR Spectrum of **7g**. **Supporting Fig. S20:** ^1^H (CDCl_3_, 400 MHz) and ^13^C{^1^H} (CDCl_3_, 101 MHz) NMR Spectrum of **7h**. **Supporting Fig. S21:** ^1^H (CDCl_3_, 400 MHz) and ^13^C{^1^H} (CDCl_3_, 101 MHz) NMR Spectrum of **7i**. **Supporting Fig. S22:** ^1^H (CDCl_3_, 400 MHz) and ^13^C{^1^H} (CDCl_3_, 101 MHz) NMR Spectrum of **7j**. **Supporting Fig. S23:** ^1^H (CDCl_3_, 400 MHz) and ^13^C{^1^H} (CDCl_3_, 101 MHz) NMR Spectrum of **7k**. **Supporting Fig. S24:** ^1^H (CDCl_3_, 400 MHz) and ^13^C{^1^H} (CDCl_3_, 101 MHz) NMR Spectrum of **7l**. **Supporting Fig. S25:** ^1^H (CDCl_3_, 400 MHz) and ^13^C{^1^H} (CDCl_3_, 101 MHz) NMR Spectrum of **7m**. **Supporting Fig. S26**: ^1^H (CDCl_3_, 400 MHz) and ^13^C{^1^H} (CDCl_3_, 101 MHz) NMR Spectrum of **7n**. **Supporting Fig. S27:** ^1^H (CDCl_3_, 400 MHz) and ^13^C{^1^H} (CDCl_3_, 101 MHz) NMR Spectrum of **7o**. **Supporting Fig. S28:** ^1^H (CDCl_3_, 400 MHz) and ^13^C{^1^H} (CDCl_3_, 101 MHz) NMR Spectrum of **7p**. **Supporting Fig. S29:** ^1^H (CDCl_3_, 400 MHz) and ^13^C{^1^H} (CDCl_3_, 101 MHz) NMR Spectrum of **7q**. **Supporting Fig. S30:** ^1^H (CDCl_3_, 400 MHz) and ^13^C{^1^H} (CDCl_3_, 101 MHz) NMR Spectrum of **8a**. **Supporting Fig. S31:** ^1^H (CDCl_3_, 400 MHz) and ^13^C{^1^H} (CDCl_3_, 101 MHz) NMR Spectrum of **8b**. **Supporting Fig. S32:** ^1^H (CDCl_3_, 400 MHz) and ^13^C{^1^H} (CDCl_3_, 101 MHz) NMR Spectrum of **8c**. **Supporting Fig. S33:** ^1^H (CDCl_3_, 400 MHz) and ^13^C{^1^H} (CDCl_3_, 101 MHz) NMR Spectrum of **8d**. **Supporting Fig. S34:** ^1^H (CDCl_3_, 400 MHz) and ^13^C{^1^H} (CDCl_3_, 101 MHz) NMR Spectrum of **8e**. **Supporting Fig. S35:** ^1^H (CDCl_3_, 400 MHz) and ^13^C{^1^H} (CDCl_3_, 101 MHz) NMR Spectrum of **8f**. **Supporting Fig. S36:** ^1^H (CDCl_3_, 400 MHz) and ^13^C{^1^H} (CDCl_3_, 101 MHz) NMR Spectrum of **8g**. **Supporting Fig. S37:** ^1^H (CDCl_3_, 400 MHz) and ^13^C{^1^H} (CDCl_3_, 101 MHz) NMR Spectrum of **8h**. **Supporting Fig. S38:** ^1^H (CDCl_3_, 400 MHz) and ^13^C{^1^H} (CDCl_3_, 101 MHz) NMR Spectrum of **8i**. **Supporting Fig. S39:** ^1^H (CDCl_3_, 400 MHz) and ^13^C{^1^H} (CDCl_3_, 101 MHz) NMR Spectrum of **8j**. **Supporting Fig. S40:** ^1^H (CDCl_3_, 400 MHz), ^13^C{^1^H} (CDCl_3_, 101 MHz), and 19^F^{^1^H} (CDCl^3^, 376 MHz) NMR Spectrum of **8k**. **Supporting Fig. S41**: ^1^H (CDCl_3_, 400 MHz), ^13^C{^1^H} (CDCl_3_, 101 MHz), and ^19^ ^F^{^1^H} (CDCl_3_, 376 MHz) NMR Spectrum of **9k**. **Supporting Fig. S42:** ^1^H (CDCl_3_, 400 MHz), ^13^C{^1^H} (CDCl_3_, 101 MHz), and ^19F^{^1^H} (CDCl_3_, 376 MHz) NMR Spectrum of **8l**. **Supporting Table S1:** Screening of electrodes, applied charge, current density, base, electrolyte, and solvent. **Supporting Table S2:** Screening of the stoichiometry of morpholine, DBU, SO_2_, and other parameters. **Supporting Table S3:** Control experiments. **Supporting Table S4:** Crystal data for **7a** (CCDC no. 2472612, the thermal ellipsoid drawn at 50% probability level).

## Conflicts of Interest

The authors declare no conflicts of interest.

## Supporting information

Supplementary Material

## Data Availability

The data that support the findings of this study are openly available in [Chemotion Repository] at [https://dx.doi.org/10.14272/collection/PCC_2025‐06‐20] [54]. All DOIs minted for the data are linked in Supporting Information.
