# Supplementary material for: Electrochemical Three‐Component Synthesis of Vinyl Sulfonamides via Decarboxylative Sulfonylation of Cinnamic Acids
Source: ChemSusChem. 2025 Dec 19;19(6):e202501920. doi: 10.1002/cssc.202501920 (PMC13021357; doi:10.1002/cssc.202501920)
Supplement: Supplementary file 1 — Supplementary Material [file CSSC-19-e202501920-s001.pdf]

Electrochemical Three-Component Synthesis of Vinyl Sulfonamides *via* Decarboxylative Sulfonylation of Cinnamic Acids

Po-Chung Chien, Harald Kelm, Georg Manolikakes*

Department of Chemistry
RPTU University Kaiserslautern-Landau
Erwin-Schrödinger-Str. 54
D-67663 Kaiserslautern (Germany)
manolikakes@chemie.uni-kl.de

Table of Contents

1 General Information ... 3
1.1. Instruments and Analytical Methods ... 3
1.2. Electrochemical Setup ... 5
2 Preparation of the SO_2 Stock Solutions ... 7
2.1 The Procedure for Preparation of the SO_2 Stock Solutions ... 7
2.2 Determination of the SO_2 Concentration of the Stock Solution ... 7
3 Synthesis of Starting Materials ... 7
3.1 Typical Procedure for the Preparation of Naphthylacrylic Acid (TP-1) ... 7
3.2 Typical Procedure for the Preparation of Acetoxyphenylacrylic Acid (TP-2) ... 8
4 Optimization of Reaction Conditions ... 9
4.1 Typical Procedure for the Optimization of Reaction Conditions (TP-3) ... 9
4.2 Optimization of Reaction Conditions ... 10

5 Synthesis of Alkyl Vinyl Sulfonamides [Scope of Cinnamic Acids and Related Substrates] 11

5.1 Typical Procedure for the Preparation of Alkyl Vinyl Sulfonamides [Scope of Cinnamic Acids and Related Substrates] (TP-4) 11

5.2 Analytical Data for Compounds of Type 7 13

5.3 Scale-up Reaction 28

5.4 Reusability Tests 29

6 Synthesis of Alkyl Vinyl Sulfonamides [Scope of Amines] 31

6.1 Typical Procedure for the Preparation of Alkyl Vinyl Sulfonamides [Scope of Amines] (TP-5) 31

6.2 Analytical Data for Compounds of Type 8 and 9 32

7 Unsuccessful Substrates 44

8 Control Experiments 45

9 Cyclic Voltammetry Results 46

10 References 50

11 X-ray crystallographic data for selected compounds 51

12 ^{1}H, ^{13}C NMR, and ^{19}F NMR spectra for compounds 7, 8, and 9 52

1 General Information

Unless otherwise mentioned, all reactions were performed under ambient conditions, and chemicals of analytical grade were used as purchased without further purification. All yields refer to isolated yields of compounds estimated to be > 95% pure as determined by ^{1}H-NMR.

1.1. Instruments and Analytical Methods

Chromatography: Thin-layer chromatography was performed on a precoated aluminum-backed silica gel plate (Merck 60 F254, 0.2 mm thickness), which was visualized by fluorescence quenching. Flash chromatography was performed on silica gel (Macherey-Nagel Kieselgel 60 0.063–0.2 mm).

Solvents: Anhydrous acetonitrile (CH_3CN), purchased from Thermo Fisher Scientific, was used without any further purification. The solvents for column chromatography were technical standards.

Materials: Starting materials, which were not commercially available, were synthesized according to the previously reported methods.[40,55,56]

NMR spectroscopy: Spectra of proton (^{1}H), carbon (^{13}C), and fluorine (^{19}F) nuclear magnetic resonance were recorded at 400 MHz, 101 MHz, and 376 MHz, respectively. Chemical shifts are reported in δ ppm referenced to an internal standard, such as TMS for ^{1}H-NMR (δ = 0.0 ppm), $CDCl_3$ for ^{13}C-NMR (δ = 77.16 ppm), DMSO-d_6 for ^{1}H-NMR (δ = 2.50 ppm), and DMSO-d_6 for ^{13}C-NMR (δ = 39.52 ppm). The coupling constants (*J*) are reported in Hz, and the following abbreviations were used to explain the multiplicities: s (singlet), d (doublet), t (triplet), q (quartet), m (multiplet), dd (doublet of doublet), and td (triplet of doublet).

Melting points: Melting points were measured on a hot stage melting point apparatus and are uncorrected.

Mass spectrometry: Mass spectra (MS) were measured using atmospheric-pressure chemical ionization (APCI) techniques with a quadrupole mass analyzer. High resolution mass spectra (HRMS) were measured using atmospheric-pressure chemical ionization (APCI) and electrospray ionization (ESI) with a 6200 series TOF and 6500 series Q-TOF analyzer.

Gas Chromatography coupled with Mass Spectrometry (GC/MS): Analysis of crude reaction mixtures was performed using Thermo Scientific ISQ 7000 equipped with an electron ionization (EI) source and a quadrupole mass analyzer.

Infrared spectroscopy: Infrared spectra (IR) of neat substances were recorded on an FT-IR (Fourier transform infrared spectroscopy) spectrometer equipped with a diamond universal ATR sampling technique (attenuated total reflectance). The absorption bands are reported in wave numbers (cm^{-1}), and only selected peaks are shown.

X-ray Crystallography: The measurement of the crystal structure was carried out on a Rigaku/Oxford diffraction Xcalibur/Gemini dual wavelength diffractometer with a Mo-Kα (λ = 0.71073 Å) radiation X-ray source.

Cyclic Voltammetry (CV) Measurements: Cyclic voltammetry was performed using Interface 1010T (Gamry Instruments, Pennsylvania, United States of America). WE: Pt electrode; CE: Pt electrode; RE: Ag/AgCl; Scan rate v = 100 mV/s. All data is displayed against the half-wave potential of ferrocene/ferrocenium redox couple (FcH/FcH^+; 0.02 V vs. Ag/AgCl) as the internal reference. Oxidation potentials are marked and displayed as the half-wave potential of the respective peak.

1.2. Electrochemical Setup

Screening and small-scale reactions: Electrochemical reactions were carried out using an IKA ElectraSyn 2.0. Parallel reactions were performed with the IKA Carousell. The divided cell used for screening or small-scale reactions was an IKA Pro-Divide equipped with a P4 glass frit (pore size 10-16 microns). All used electrodes were purchased from IKA. Graphite (SK-50) electrodes from IKA were used for small-scale reactions.

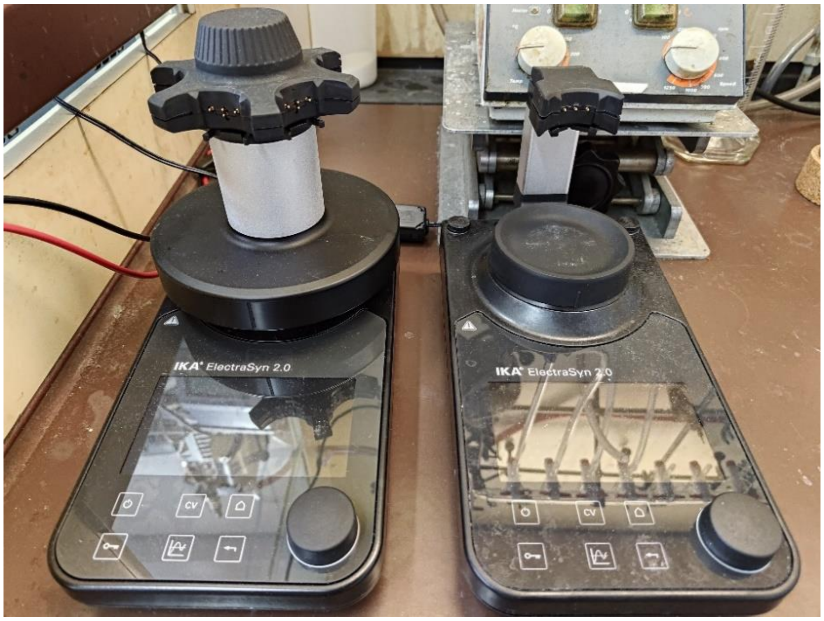

Figure S1: IKA ElectraSyn 2.0 and IKA Carousell.

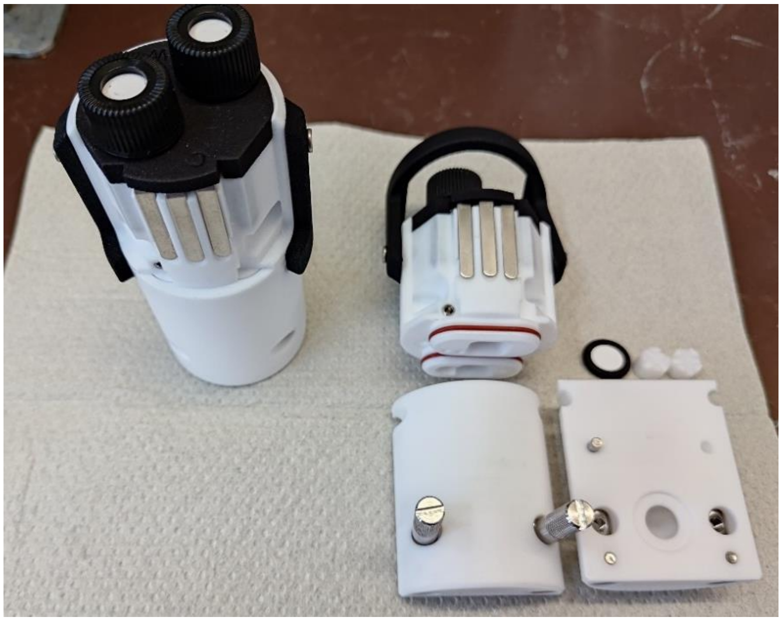

Figure S2: IKA Pro-Divide divided cell.

Scale-up cell: The scale-up experiment was performed in a divided glass cell with a volume of 100 mL per half-cell equipped with caps, silicone septa, electrodes, electrode holders, a glass frit (P4, d = 24 mm), and stirring bars. The electrolysis was conducted using Rohde & Schwarz HMP4040 (Rohde & Schwarz, München, Bayern, Deutschland) as the power source. The electrode dimensions were 52 mm × 8 mm × 2 mm, with 38 mm submerged into the reaction mixture, resulting in an active electrode area of 3.0 cm^2. The graphite (SK-50) electrodes were purchased from IKA. The holders are available at Sigma-Aldrich within the SynLectro™ series.

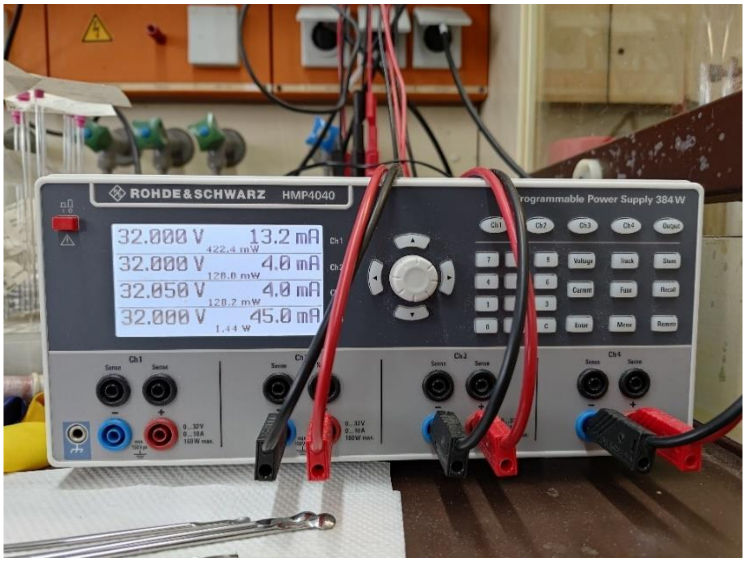

Figure S3: Rohde & Schwarz HMP4040 power source.

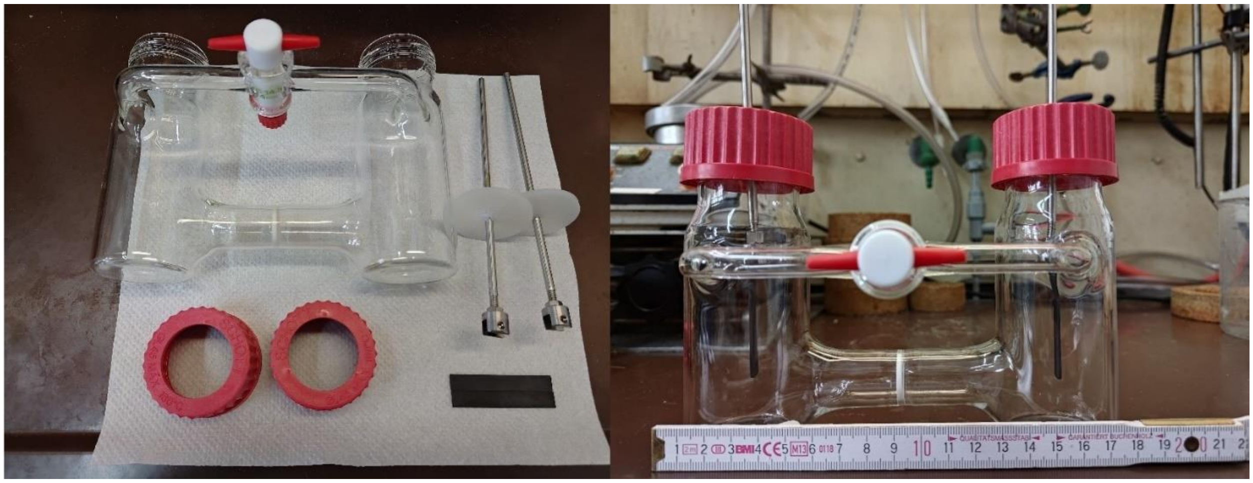

Figure S4: Electrochemical glass cell used for the scale-up experiment equipped with a P4 frit, graphite electrodes, caps, silicone septa, and electrode holders. Scale is given in cm.

2 Preparation of the SO_2 Stock Solutions

2.1 The Procedure for Preparation of the SO_2 Stock Solutions

300 mL of anhydrous acetonitrile and molecular sieves (3 Å, 50.0 g) were transferred into a dry two-neck round-bottom flask. Sulfur dioxide was bubbled into the solution at a slow flow rate for 15 minutes at 0 °C. Excess gaseous sulfur dioxide leaving the apparatus was purged with aq. NaOH solution.

2.2 Determination of the SO_2 Concentration of the Stock Solution

The exact concentration of the prepared SO_2 solution was determined according to iodometric titration.[57] To a solution of I_2 (1.27 g, 5.00 mmol) and KI (2.20 g, 13.3 mmol) in H_2O (100 mL) was slowly added the freshly prepared SO_2 stock solution (1.0 mL). The resulting solution was then titrated with a freshly prepared aq. $Na_2S_2O_3$ solution (0.2 M) as titrant to determine the amount of excess I_2 (end point marked by a color change from brown to colorless; optionally, starch can be added for better visualization). Titrations of individual batches of stock solutions were repeated three times for a more accurate determination of the concentration.

3 Synthesis of Starting Materials

3.1 Typical Procedure for the Preparation of Naphthylacrylic Acid (TP-1)

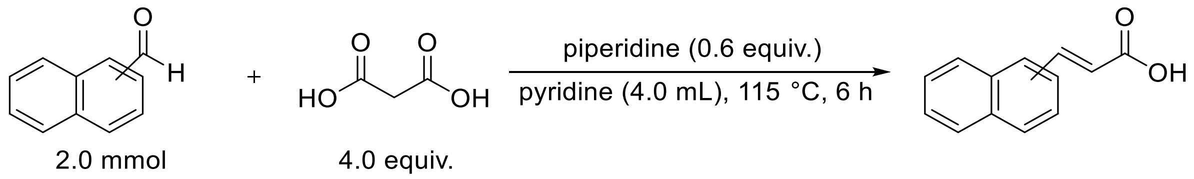

The naphthylacrylic acid was synthesized according to the reported literature.[40,55]

A 25.0 mL single-neck round-bottomed flask equipped with a magnetic stir bar was charged with the corresponding naphthaldehyde (312.4 mg, 2.0 mmol), malonic acid (832.5 mg, 4.0 equiv.), piperidine (0.13 mL, 0.6 equiv.), and pyridine (4.0 mL) and heated to 115 °C for 6 h. Afterwards, the reaction mixture was cooled to room temperature, poured into an Erlenmeyer flask, and acidified with 2.0 M HCl to a final pH = 1. The resulting precipitate was filtered and washed with distilled water (30.0 mL) to afford the naphthylacrylic acid.

3.2 Typical Procedure for the Preparation of Acetoxyphenylacrylic Acid (TP-2)

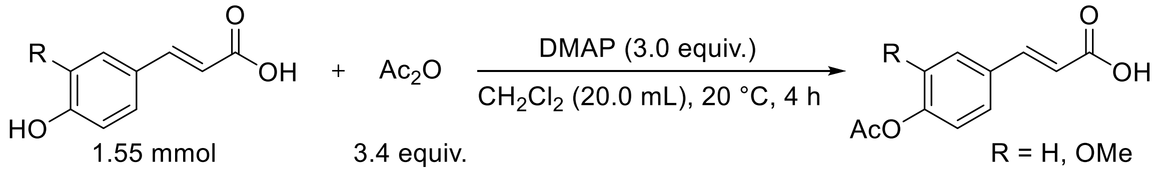

The acetoxyphenylacrylic acid was synthesized according to the reported literature.[40,56]

A 50.0 mL single-neck round-bottomed flask equipped with a magnetic stir bar was charged with hydroxycinnamic acid (1.55 mmol), CH_2Cl_2 (20.0 mL), acetic anhydride (0.5 mL, 3.4 equiv.), and DMAP (568.1 mg, 3.0 equiv.). The resulting solution was stirred at 20 °C for 4 h. After completion of the reaction, the reaction mixture was acidified (pH = 1) with 2.0 M HCl and extracted with CH_2Cl_2 (3 x 20.0 mL). The organic layer was dried over anhydrous Na_2SO_4 and concentrated in *vacuo*. The crude residue was subjected to flash column chromatography on silica gel to obtain acetoxyphenylacrylic acid.

4 Optimization of Reaction Conditions

4.1 Typical Procedure for the Optimization of Reaction Conditions (TP-3)

The reactions were carried out using IKA Pro-Divide cells with a P4 glass frit.

Anolyte: An oven-dried 10.0 mL pear-shaped flask was charged with cinnamic acid (44.5 mg, 0.3 mmol), morpholine, ammonium salt, and anhydrous acetonitrile. The mixture was cooled to 0 °C in an ice bath, followed by the addition of SO_2 stock solution (4.6 M in acetonitrile) and base so that a total volume of 3.0 mL was achieved.

Catholyte: An oven-dried 10.0 mL pear-shaped flask was charged with ammonium salt, acetic acid (5.0 equiv.), and anhydrous acetonitrile so that a total volume of 3.0 mL was achieved as well.

The reaction mixtures were transferred with syringes to their respective compartment simultaneously. The amperage and amount of applied charge were set to the desired values, and the electrolysis was conducted at room temperature under constant stirring (400 rpm).

After completion of the electrolysis, the anolyte was transferred to a round-bottom flask, and the anode compartment was rinsed with additional EtOAc (2 x 3.0 mL). ^{1}H NMR yields were calculated by the addition of triphenylmethane (73.2 mg, 1.0 equiv.) as the internal standard to this mixture. After 5 min of stirring, 1.0 mL of the mixture was taken, and the solvent was removed under reduced pressure. The crude residue was dissolved in $CDCl_3$ for ^{1}H NMR experiments.

4.2 Optimization of Reaction Conditions

Optimization of the conditions was carried out by using linear screening with cinnamic acid and morpholine as substrates according to the procedure described in **TP-3**.

Table S1: Screening of electrodes, applied charge, current density, base, electrolyte, and solvent.

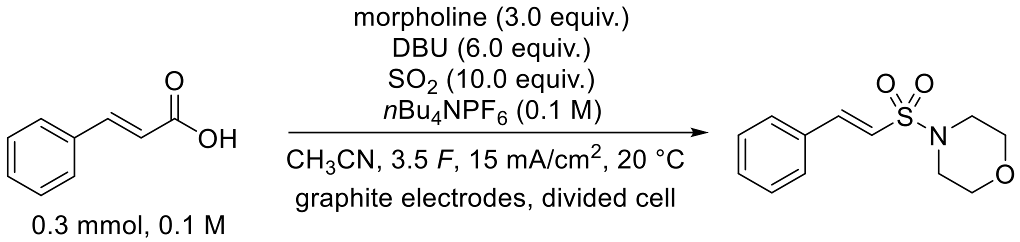

Entry	Deviation from the standard conditions	Yield (%)[a]	Cinnamic acid (%)[a]
**1**	**None**	**76 (73)**[b]	**0**
2	BDD electrodes	0	47
3	Glassy carbon electrodes	10	44
4	Pt foil electrodes	9	52
5	Graphite anode; Pt foil cathode	51	17
6	3.0 *F*	60	0
7	3.2 *F*	68	0
8	3.8 *F*	63	0
9	10 mA/cm^2	65	0
10	12.5 mA/cm^2	68	0
11	17.5 mA/cm^2	67	0
12	20 mA/cm^2	55	0
13	2,6-Lutidine	31	12
14	Pyridine	13	46
15	DBN	51	0
16	DIPEA	0	0
17	nBu$_4$NBF$_4$	56	0
18	DMF	24	14

[a] ^{1}H-NMR yield with the use of $CHPh_3$ as the internal standard. [b] Isolated yield.

Table S2: Screening of the stoichiometry of morpholine, DBU, SO_2, and other parameters.

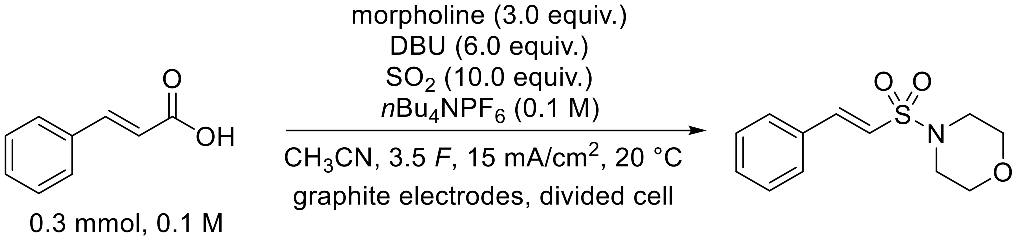

Entry	Deviation from the standard conditions	Yield (%)[a]	Cinnamic acid (%)[a]
**1**	**None**	**76 (73)**[b]	**0**
2	nBu$_4$NPF$_6$ (0.05 M)	57	0
3	nBu$_4$NPF$_6$ (0.2 M)	58	0
4	Morpholine (2.0 equiv.)	51	0
5	Morpholine (4.0 equiv.)	54	0
6	DBU (4.0 equiv.)	44	0
7	DBU (8.0 equiv.)	59	0
8	SO_2 (7.5 equiv.)	56	0
9	SO_2 (12.5 equiv.)	73	0
10	Styrene (0.3 mmol) instead of cinnamic acid	Traces	0
11	An undivided cell was used	32	47

[a] ^{1}H-NMR yield with the use of $CHPh_3$ as the internal standard. [b] Isolated yield.

5 Synthesis of Alkyl Vinyl Sulfonamides [Scope of Cinnamic Acids and Related Substrates]

5.1 Typical Procedure for the Preparation of Alkyl Vinyl Sulfonamides [Scope of Cinnamic Acids and Related Substrates] (TP-4)

The reactions were carried out using IKA Pro-Divide cells with a P4 glass frit.

Anolyte: An oven-dried 10.0 mL pear-shaped flask was charged with morpholine (78.5 µL, 3.0 equiv.), nBu$_4$NPF$_6$ (116.3 mg, 1.0 equiv.), and anhydrous acetonitrile (2.0 mL). The mixture was cooled to 0 °C in an ice bath, followed by the addition of SO_2 stock solution (0.65 mL, 4.6 M in acetonitrile, 10.0 equiv.) and DBU (0.27 mL, 6.0 equiv.) so that a total volume of 3.0 mL was achieved.

Catholyte: An oven-dried 10.0 mL pear-shaped flask was charged with nBu$_4$NPF$_6$ (116.3 mg, 1.0 equiv.), acetic acid (0.09 mL, 5.0 equiv.), and anhydrous acetonitrile (2.9 mL) so that a total volume of 3.0 mL was achieved as well.

The reaction mixtures were transferred with syringes to their respective compartment simultaneously, and the anolyte was additionally loaded with the respective acid (0.3 mmol). The amperage was set accordingly so that a current density of 15 mA×cm^{-2} was reached (13.2 mA with the setup described herein), and the amount of applied charge was set to 3.5 *F*. (For the synthesis of compound **7j**, 3.0 *F* was used to minimize product degradation under the reaction conditions.) The electrolysis was conducted at room temperature under constant stirring (400 rpm) for ca. 2 h 8 min.

After completion of the electrolysis, the anolyte was transferred to a separatory funnel, and the anode compartment was rinsed with additional EtOAc (2 x 3.0 mL). Distilled water (10.0 mL) was added, and the obtained mixture was extracted with EtOAc (2 x 10.0 mL). The organic layer was dried over anhydrous Na_2SO_4 and concentrated in *vacuo*. The crude residue was subjected to flash column chromatography on silica gel to obtain the product.

Compounds **7a**, **7b**, **7f**, **7g**, **7h**, **7i**, **7k**, **7l**, **7m**, **7n**, **7o**, **7p**, and **7q** contained some unidentified impurities after column chromatography. Additional recrystallization was necessary to remove these impurities. Therefore, the product obtained after chromatography was dissolved in a minimal amount of EtOAc (typically 4-6 mL) and heated in an oil bath at 80 ˚C. When the solution became turbid, *n*-hexane (2 mL) was added. After cooling the solution, the solid was filtered to obtain the desired product.

5.2 Analytical Data for Compounds of Type 7

(*E*)-4-(Styrylsulfonyl)morpholine (7a)

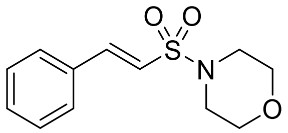

Following the **TP-4**, cinnamic acid (44.5 mg, 0.3 mmol), morpholine (78.5 µL, 3.0 equiv.), and SO_2 stock solution (0.65 mL, 4.6 M in acetonitrile, 10.0 equiv.) were used as substrates. The residue was purified by flash chromatography over silica gel (*n*-hexane/EtOAc = 80:20) and recrystallized with EtOAc/*n*-hexane to obtain **7a** as a colorless solid (55.3 mg, 73% yield).

mp: 110.5–110.8 °C. R_f = 0.33 (*n*-hexane/EtOAc = 7:3).

^{1}H NMR (400 MHz, $CDCl_3$): δ 7.55–7.38 (m, 6H), 6.68 (d, *J* = 15.6 Hz, 1H), 3.83–3.73 (m, 4H), 3.23–3.16 (m, 4H).

^{13}C{^{1}H}-NMR (101 MHz, $CDCl_3$): δ 144.4, 132.5, 131.2, 129.2, 128.4, 120.6, 66.4, 45.8.

IR (ATR) $\tilde{\nu}$ (cm^{-1}): 2967, 1342, 1148, 1111, 936, 749.

MS (APCI): *m/z* calcd. for $C_{12}H_{16}NO_3S$ ($[M+H]^+$) 254.1, found 254.1.

HRMS (APCI): *m/z* calcd. for $C_{12}H_{16}NO_3S$ ($[M+H]^+$) 254.0845, found 254.0838.

Additional information on the chemical synthesis is available *via* Chemotion repository:
https://doi.org/10.14272/reaction/SA-FUHFF-UHFFFADPSC-RDTYOSMNVS-UHFFFADPSC-NUHFF-NXEWS-NUHFF-ZZZ

Additional information on the analysis of the target compound is available *via* Chemotion repository:
https://doi.org/10.14272/RDTYOSMNVSOCAV-IZZDOVSWSA-N.1

(*E*)-4-((4-Methylstyryl)sulfonyl)morpholine (7b)

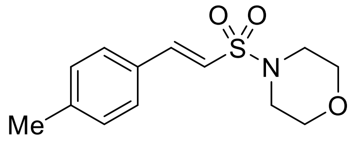

Following the **TP-4**, (*E*)-3-(*p*-tolyl)acrylic acid (48.7 mg, 0.3 mmol), morpholine (78.5 µL, 3.0 equiv.), and SO_2 stock solution (0.65 mL, 4.6 M in acetonitrile, 10.0 equiv.) were used as substrates. The residue was purified by flash chromatography over silica gel (*n*-hexane/EtOAc = 80:20) and recrystallized with EtOAc/*n*-hexane to obtain **7b** as a colorless solid (46.5 mg, 58% yield).

mp: 144.9–145.2 °C. R_f = 0.25 (*n*-hexane/EtOAc = 8:2).

^{1}H NMR (400 MHz, $CDCl_3$): δ 7.45 (d, *J* = 15.6 Hz, 1H), 7.42–7.37 (m, 2H), 7.25–7.20 (m, 2H), 6.62 (d, *J* = 15.6 Hz, 1H), 3.81–3.75 (m, 4H), 3.23–3.15 (m, 4H), 2.39 (s, 3H).

^{13}C{^{1}H}-NMR (101 MHz, $CDCl_3$): δ 144.5, 141.9, 123.0, 129.7, 128.4, 119.2, 66.4, 45.8, 21.6.

IR (ATR) $\tilde{\nu}$ (cm^{-1}): 3052, 2922, 1342, 1145, 1109, 939, 798.

MS (APCI): *m/z* calcd. for $C_{13}H_{18}NO_3S$ ($[M+H]^+$) 268.1, found 268.1.

HRMS (APCI): *m/z* calcd. for $C_{13}H_{18}NO_3S$ ($[M+H]^+$) 268.1002, found 268.0995.

Additional information on the chemical synthesis is available *via* Chemotion repository:
https://doi.org/10.14272/reaction/SA-FUHFF-UHFFFADPSC-NYIYZYBSNG-UHFFFADPSC-NUHFF-NXEWS-NUHFF-ZZZ

Additional information on the analysis of the target compound is available *via* Chemotion repository:
https://doi.org/10.14272/NYIYZYBSNGRSHY-IZZDOVSWSA-N.1

(*E*)-4-((4-Methoxystyryl)sulfonyl)morpholine (7c)

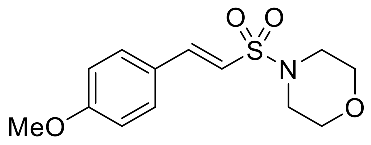

Following the **TP-4**, (*E*)-3-(4-methoxyphenyl)acrylic acid (53.5 mg, 0.3 mmol), morpholine (78.5 µL, 3.0 equiv.), and SO_2 stock solution (0.65 mL, 4.6 M in acetonitrile, 10.0 equiv.) were used as substrates. The residue was purified by flash chromatography over silica gel (*n*-hexane/EtOAc =

80:20) to obtain **7c** as a colorless solid (49.2 mg, 58% yield).

mp: 149.0–149.3 °C. R_f = 0.13 (*n*-hexane/EtOAc = 8:2).

^{1}H NMR (400 MHz, $CDCl_3$): δ 7.50–7.44 (m, 2H), 7.42 (d, *J* = 15.4 Hz, 1H), 6.98–6.88 (m, 2H), 6.53 (d, *J* = 15.4 Hz, 1H), 3.85 (s, 3H), 3.82–3.72 (m, 4H), 3.22–3.12 (m, 4H).

$^{13}C\{^1H\}$-NMR (101 MHz, $CDCl_3$): δ 162.1, 144.1, 130.2, 125.1, 117.5, 114.6, 66.3, 55.5, 45.8.

IR (ATR) $\tilde{\nu}$ (cm^{-1}): 3060, 2923, 1324, 1141, 1108, 941, 803.

MS (APCI): *m/z* calcd. for $C_{13}H_{18}NO_4S$ ($[M+H]^+$) 284.1, found 284.1.

HRMS (APCI): *m/z* calcd. for $C_{13}H_{18}NO_4S$ ($[M+H]^+$) 284.0951, found 284.0949.

Additional information on the chemical synthesis is available *via* Chemotion repository:
https://doi.org/10.14272/reaction/SA-FUHFF-UHFFFADPSC-FJRTYDMRXB-UHFFFADPSC-NUHFF-NXEWS-NUHFF-ZZZ

Additional information on the analysis of the target compound is available *via* Chemotion repository:
https://doi.org/10.14272/FJRTYDMRXBMGDN-IZZDOVSWSA-N.1

(*E*)-4-((4-Nitrostyryl)sulfonyl)morpholine (7d)

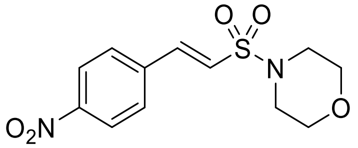

Following the **TP-4**, (*E*)-3-(4-nitrophenyl)acrylic acid (58.0 mg, 0.3 mmol), morpholine (78.5 µL, 3.0 equiv.), and SO_2 stock solution (0.65 mL, 4.6 M in acetonitrile, 10.0 equiv.) were used as substrates. The residue was purified by flash chromatography over silica gel (*n*-hexane/EtOAc = 80:20) to obtain **7d** as a colorless solid (41.6 mg, 46% yield).

mp: 210.2–210.5 °C. R_f = 0.13 (*n*-hexane/EtOAc = 8:2).

^{1}H NMR (400 MHz, DMSO-d_6): δ 8.31–8.25 (m, 2H), 8.09–8.04 (m, 2H), 7.64–7.54 (m, 2H), 3.71–3.64 (m, 4H), 3.15–3.08 (m, 4H).

$^{13}C\{^1H\}$-NMR (101 MHz, DMSO-d_6): δ 148.3, 140.5, 139.1, 129.9, 126.5, 123.9, 65.5, 45.4.

IR (ATR) $\tilde{\nu}$ (cm^{-1}): 3046, 2966, 1511, 1344, 1151, 1111, 938, 800.

MS (APCI): *m/z* calcd. for $C_{12}H_{15}N_2O_5S$ ($[M+H]^+$) 299.1, found 299.1.

HRMS (APCI): *m/z* calcd. for $C_{12}H_{15}N_2O_5S$ ($[M+H]^+$) 299.0696, found 299.0691.

Additional information on the chemical synthesis is available *via* Chemotion repository:
https://doi.org/10.14272/reaction/SA-FUHFF-UHFFFADPSC-JALYXQMJMK-UHFFFADPSC-NUHFF-NOICR-NUHFF-ZZZ

Additional information on the analysis of the target compound is available *via* Chemotion repository:
https://doi.org/10.14272/JALYXQMJMKUPGC-BJMVGYQFSA-N.1

(*E*)-4-((4-Fluorostyryl)sulfonyl)morpholine (7e)

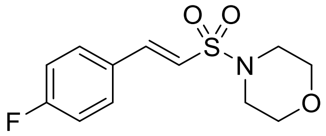

Following the **TP-4**, (*E*)-3-(4-fluorophenyl)acrylic acid (49.8 mg, 0.3 mmol), morpholine (78.5 µL, 3.0 equiv.), and SO_2 stock solution (0.65 mL, 4.6 M in acetonitrile, 10.0 equiv.) were used as substrates. The residue was purified by flash chromatography over silica gel (*n*-hexane/EtOAc = 80:20) to obtain **7e** as a colorless solid (40.9 mg, 50% yield).

mp: 106.2–106.5 °C. R_f = 0.15 (*n*-hexane/EtOAc = 8:2).

^{1}H NMR (400 MHz, $CDCl_3$): δ 7.55–7.48 (m, 2H), 7.45 (d, *J* = 15.6 Hz, 1H), 7.17–7.08 (m, 2H), 6.62 (d, *J* = 15.6 Hz, 1H), 3.83–3.74 (m, 4H), 3.25–3.15 (m, 4H).

^{13}C{^{1}H}-NMR (101 MHz, $CDCl_3$): δ 164.4 (d, $^1J_{C-F}$ = 253.0 Hz, 1C), 143.0, 130.4 (d, $^3J_{C-F}$ = 8.6 Hz, 2C), 128.7 (d, $^4J_{C-F}$ = 3.4 Hz, 1C), 120.3 (d, $^5J_{C-F}$ = 2.2 Hz, 1C), 116.5 (d, $^2J_{C-F}$ = 22.0 Hz, 2C), 66.3, 45.8.

^{19}F{^{1}H}-NMR (376 MHz, $CDCl_3$): δ -107.9.

IR (ATR) $\tilde{\nu}$ (cm^{-1}): 3053, 2922, 1342, 1325, 1145, 1109, 938, 800.

MS (APCI): *m/z* calcd. for $C_{12}H_{15}NO_3FS$ ($[M+H]^+$) 272.1, found 272.0.

HRMS (APCI): *m/z* calcd. for $C_{12}H_{15}NO_3FS$ ($[M+H]^+$) 272.0751, found 272.0746.

Additional information on the chemical synthesis is available *via* Chemotion repository:
https://doi.org/10.14272/reaction/SA-FUHFF-UHFFFADPSC-GTZHODGUUK-

UHFFFADPSC-NUHFF-NOICR-NUHFF-ZZZ

Additional information on the analysis of the target compound is available *via* Chemotion repository:

https://doi.org/10.14272/GTZHODGUUKKNIW-BJMVGYQFSA-N.1

(*E*)-4-((4-Chlorostyryl)sulfonyl)morpholine (7f)

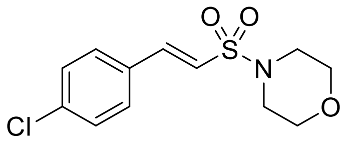

Following the **TP-4**, (*E*)-3-(4-chlorophenyl)acrylic acid (54.8 mg, 0.3 mmol), morpholine (78.5 µL, 3.0 equiv.), and SO_2 stock solution (0.65 mL, 4.6 M in acetonitrile, 10.0 equiv.) were used as substrates. The residue was purified by flash chromatography over silica gel (*n*-hexane/EtOAc = 80:20) and recrystallized with EtOAc/*n*-hexane to obtain **7f** as a colorless solid (42.6 mg, 49% yield).

mp: 145.6–146.0 °C. R_f = 0.25 (*n*-hexane/EtOAc = 8:2).

^{1}H NMR (400 MHz, $CDCl_3$): δ 7.50–7.36 (m, 5H), 6.66 (d, *J* = 15.6 Hz, 1H), 3.83–3.75 (m, 4H), 3.24–3.16 (m, 4H).

^{13}C{^{1}H}-NMR (101 MHz, $CDCl_3$): δ 142.9, 137.3, 131.0, 129.62, 129.61, 121.3, 66.4, 45.8.

IR (ATR) $\tilde{\nu}$ (cm^{-1}): 3049, 2923, 1345, 1149, 1112, 936, 802, 728.

MS (APCI): *m/z* calcd. for $C_{12}H_{15}NO_3SCl$ ($[M+H]^+$) 288.0, found 288.0.

HRMS (APCI): *m/z* calcd. for $C_{12}H_{15}NO_3SCl$ ($[M+H]^+$) 288.0456, found 288.0451.

Additional information on the chemical synthesis is available *via* Chemotion repository:

https://doi.org/10.14272/reaction/SA-FUHFF-UHFFFADPSC-KKKZCTOWMI-UHFFFADPSC-NUHFF-NOICR-NUHFF-ZZZ

Additional information on the analysis of the target compound is available *via* Chemotion repository:

https://doi.org/10.14272/KKKZCTOWMICLBA-BJMVGYQFSA-N.1

(*E*)-4-((4-Bromostyryl)sulfonyl)morpholine (7g)

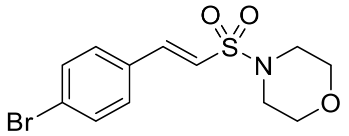

Following the **TP-4**, (*E*)-3-(4-bromophenyl)acrylic acid (68.1 mg, 0.3 mmol), morpholine (78.5 µL, 3.0 equiv.), and SO_2 stock solution (0.65 mL, 4.6 M in acetonitrile, 10.0 equiv.) were used as substrates. The residue was purified by flash chromatography over silica gel (*n*-hexane/EtOAc = 80:20) and recrystallized with EtOAc/*n*-hexane to obtain **7g** as a colorless solid (48.1 mg, 48% yield).

mp: 162.0–162.3 °C. R_f = 0.25 (*n*-hexane/EtOAc = 8:2).

^{1}H NMR (400 MHz, $CDCl_3$): δ 7.59–7.53 (m, 2H), 7.42 (d, *J* = 15.6 Hz, 1H), 7.39–7.34 (m, 2H), 6.68 (d, *J* = 15.6 Hz, 1H), 3.82–3.75 (m, 4H), 3.24–3.16 (m, 4H).

^{13}C{^{1}H}-NMR (101 MHz, $CDCl_3$): δ 142.9, 132.6, 131.4, 129.8, 125.7, 121.4, 66.4, 45.8.

IR (ATR) $\tilde{\nu}$ (cm^{-1}): 3056, 2920, 1328, 1146, 1108, 942, 793, 553.

MS (APCI): *m/z* calcd. for $C_{12}H_{15}NO_3SBr$ ($[M+H]^+$) 332.0, found 332.0.

HRMS (APCI): *m/z* calcd. for $C_{12}H_{15}NO_3SBr$ ($[M+H]^+$) 331.9951, found 331.9942.

Additional information on the chemical synthesis is available *via* Chemotion repository:
https://doi.org/10.14272/reaction/SA-FUHFF-UHFFFADPSC-RTNWQNPYGC-UHFFFADPSC-NUHFF-NOICR-NUHFF-ZZZ

Additional information on the analysis of the target compound is available *via* Chemotion repository:
https://doi.org/10.14272/RTNWQNPYGCNCPV-BJMVGYQFSA-N.1

(*E*)-4-((3-Bromostyryl)sulfonyl)morpholine (7h)

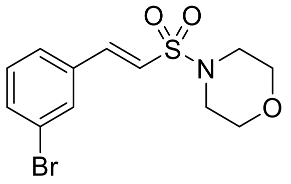

Following the **TP-4**, (*E*)-3-(3-bromophenyl)acrylic acid (68.1 mg, 0.3 mmol), morpholine (78.5 µL, 3.0 equiv.), and SO_2 stock solution (0.65 mL, 4.6 M in acetonitrile, 10.0 equiv.) were used as substrates. The residue was purified by flash chromatography over silica gel (*n*-hexane/EtOAc = 80:20) and recrystallized with EtOAc/*n*-hexane to obtain **7h** as a colorless solid (64.1 mg, 64% yield).

mp: 152.0–152.5 °C. R_f = 0.20 (*n*-hexane/EtOAc = 8:2).

^{1}H NMR (400 MHz, $CDCl_3$): δ 7.65 (s, 1H), 7.57 (d, *J* = 8.0 Hz, 1H), 7.42 (d, *J* = 8.0 Hz, 1H), 7.41 (d, *J* = 15.6 Hz, 1H), 7.31 (t, *J* = 8.0 Hz, 1H), 6.69 (d, *J* = 15.6 Hz, 1H), 3.84–3.74 (m, 4H), 3.26–3.16 (m, 4H).

^{13}C{^{1}H}-NMR (101 MHz, $CDCl_3$): δ 142.5, 134.6, 134.0, 131.0, 130.8, 127.1, 123.4, 122.4, 66.4, 45.8.

IR (ATR) $\tilde{\nu}$ (cm^{-1}): 3055, 2922, 1329, 1145, 1108, 938, 776, 527.

MS (APCI): *m/z* calcd. for $C_{12}H_{15}NO_3SBr$ ($[M+H]^+$) 332.0, found 332.0.

HRMS (APCI): *m/z* calcd. for $C_{12}H_{15}NO_3SBr$ ($[M+H]^+$) 331.9951, found 331.9942.

Additional information on the chemical synthesis is available *via* Chemotion repository:
https://doi.org/10.14272/reaction/SA-FUHFF-UHFFFADPSC-IFHKDQTWRN-UHFFFADPSC-NUHFF-NJZOK-NUHFF-ZZZ

Additional information on the analysis of the target compound is available *via* Chemotion repository:
https://doi.org/10.14272/IFHKDQTWRNRUPN-RUDMXATFSA-N.1

(*E*)-4-((2-Bromostyryl)sulfonyl)morpholine (7i)

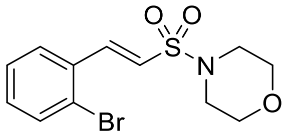

Following the **TP-4**, (*E*)-3-(2-bromophenyl)acrylic acid (68.1 mg, 0.3 mmol), morpholine (78.5 µL, 3.0 equiv.), and SO_2 stock solution (0.65 mL, 4.6 M in acetonitrile, 10.0 equiv.) were used as substrates. The residue was purified by flash chromatography over silica gel (*n*-hexane/EtOAc = 80:20) and recrystallized with EtOAc/*n*-hexane to obtain **7i** as a colorless solid (53.3 mg, 53% yield).

mp: 130.2–130.3 °C. R_f = 0.18 (*n*-hexane/EtOAc = 8:2).

^{1}H NMR (400 MHz, $CDCl_3$): δ 7.81 (d, J = 15.6 Hz, 1H), 7.65 (d, J = 7.8 Hz, 1H), 7.54 (dd, J = 7.8, 1.4 Hz, 1H), 7.37 (t, J = 7.8 Hz, 1H), 7.29 (td, J = 7.8, 1.4 Hz, 1H), 6.65 (d, J = 15.6 Hz, 1H), 3.83–3.75 (m, 4H), 3.26–3.18 (m, 4H).

^{13}C{^{1}H}-NMR (101 MHz, $CDCl_3$): δ 142.8, 133.7, 132.9, 132.0, 128.3, 128.0, 125.2, 124.0, 66.4, 45.8.

IR (ATR) $\tilde{\nu}$ (cm^{-1}): 3050, 2917, 1342, 1145, 1112, 945, 742, 527.

MS (APCI): *m/z* calcd. for $C_{12}H_{15}NO_3SBr$ ($[M+H]^+$) 332.0, found 332.0.

HRMS (APCI): *m/z* calcd. for $C_{12}H_{15}NO_3SBr$ ($[M+H]^+$) 331.9951, found 331.9943.

Additional information on the chemical synthesis is available *via* Chemotion repository:
https://doi.org/10.14272/reaction/SA-FUHFF-UHFFFADPSC-OBDSTLGDPF-UHFFFADPSC-NUHFF-NOICR-NUHFF-ZZZ

Additional information on the analysis of the target compound is available *via* Chemotion repository:
https://doi.org/10.14272/OBDSTLGDPFJPFI-BJMVGYQFSA-N.1

(*E*)-4-(2-(Morpholinosulfonyl)vinyl)phenyl acetate (7j)

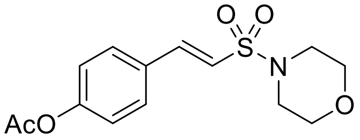

Following the **TP-4**, (*E*)-3-(4-acetoxyphenyl)acrylic acid (61.9 mg, 0.3 mmol), morpholine (78.5 µL, 3.0 equiv.), and SO_2 stock solution (0.65 mL, 4.6 M in acetonitrile, 10.0 equiv.) were used as substrates. The residue was purified by flash chromatography over silica gel (*n*-hexane/EtOAc = 75:25) to obtain **7j** as a colorless solid (33.7 mg, 36% yield).

mp: 153.0–153.5 °C. R_f = 0.50 (*n*-hexane/EtOAc = 5:5).

^{1}**H NMR** (400 MHz, $CDCl_3$): δ 7.56–7.49 (m, 2H), 7.46 (d, *J* = 15.6 Hz, 1H), 7.21–7.14 (m, 2H), 6.64 (d, *J* = 15.6 Hz, 1H), 3.82–3.74 (m, 4H), 3.23–3.15 (m, 4H), 2.32 (s, 3H).

^{13}**C{^{1}H}-NMR** (101 MHz, $CDCl_3$): δ 169.1, 152.8, 143.2, 130.2, 129.6, 122.6, 120.8, 66.4, 45.8, 21.2.

IR (ATR) $\tilde{v}$ (cm^{-1}): 3063, 2920, 1764, 1338, 1139, 1113, 945, 799.

MS (APCI): *m/z* calcd. for $C_{14}H_{18}NO_5S$ ($[M+H]^+$) 312.1, found 312.1.

HRMS (APCI): *m/z* calcd. for $C_{14}H_{18}NO_5S$ ($[M+H]^+$) 312.0900, found 312.0895.

Additional information on the chemical synthesis is available *via* Chemotion repository:
https://doi.org/10.14272/reaction/SA-FUHFF-UHFFFADPSC-HOQLEZAFYZ-UHFFFADPSC-NUHFF-NXEWS-NUHFF-ZZZ

Additional information on the analysis of the target compound is available *via* Chemotion repository:
https://doi.org/10.14272/HOQLEZAFYZRYDA-IZZDOVSWSA-N.1

(*E*)-2-Methoxy-4-(2-(morpholinosulfonyl)vinyl)phenyl acetate (7k)

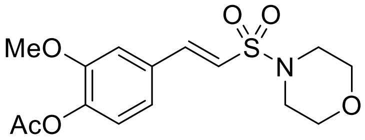

Following the **TP-4**, (*E*)-3-(4-acetoxy-3-methoxyphenyl)acrylic acid (70.9 mg, 0.3 mmol), morpholine (78.5 µL, 3.0 equiv.), and SO_2 stock solution (0.65 mL, 4.6 M in acetonitrile, 10.0

equiv.) were used as substrates. The residue was purified by flash chromatography over silica gel (*n*-hexane/EtOAc = 70:30) and recrystallized with EtOAc/*n*-hexane to obtain **7k** as a colorless solid (39.7 mg, 39% yield).

mp: 158.0–158.5 °C. R_f = 0.45 (*n*-hexane/EtOAc = 5:5).

^{1}H NMR (400 MHz, $CDCl_3$): δ 7.43 (d, *J* = 15.6 Hz, 1H), 7.14–7.04 (m, 3H), 6.64 (d, *J* = 15.6 Hz, 1H), 3.87 (s, 3H), 3.81–3.75 (m, 4H), 3.23–3.15 (m, 4H), 2.33 (s, 3H).

^{13}C{^{1}H}-NMR (101 MHz, $CDCl_3$): δ 168.8, 151.7, 143.7, 142.2, 131.4, 123.7, 121.5, 120.8, 111.7, 66.4, 56.1, 45.8, 20.8.

IR (ATR) $\tilde{\nu}$ (cm^{-1}): 3049, 2933, 1760, 1346, 1148, 1109, 938, 793.

MS (APCI): *m/z* calcd. for $C_{15}H_{20}NO_6S$ ($[M+H]^+$) 342.1, found 342.2.

HRMS (APCI): *m/z* calcd. for $C_{15}H_{20}NO_6S$ ($[M+H]^+$) 342.1006, found 342.1003.

Additional information on the chemical synthesis is available *via* Chemotion repository:
https://doi.org/10.14272/reaction/SA-FUHFF-UHFFFADPSC-NBWPABGIFD-UHFFFADPSC-NUHFF-NOICR-NUHFF-ZZZ

Additional information on the analysis of the target compound is available *via* Chemotion repository:
https://doi.org/10.14272/NBWPABGIFDZLSE-BJMVGYQFSA-N.1

(*E*)-4-((3,4,5-Trimethoxystyryl)sulfonyl)morpholine (7l)

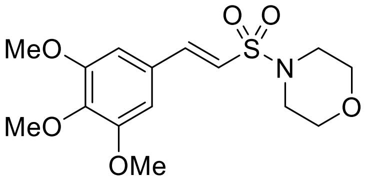

Following the **TP-4**, (*E*)-3-(3,4,5-trimethoxyphenyl)acrylic acid (71.5 mg, 0.3 mmol), morpholine (78.5 µL, 3.0 equiv.), and SO_2 stock solution (0.65 mL, 4.6 M in acetonitrile, 10.0 equiv.) were used as substrates. The residue was purified by flash chromatography over silica gel (*n*-hexane/EtOAc = 80:20) and recrystallized with EtOAc/*n*-hexane to obtain **7l** as a colorless solid (64.5 mg, 63% yield).

mp: 104.8–105.2 °C. R_f = 0.10 (*n*-hexane/EtOAc = 8:2).

^{1}H NMR (400 MHz, $CDCl_3$): δ 7.39 (d, *J* = 15.4 Hz, 1H), 6.73 (s, 2H), 6.61 (d, *J* = 15.4 Hz, 1H),

3.90 (s, 9H), 3.82–3.75 (m, 4H), 3.23–3.17 (m, 4H).

^{13}C{^{1}H}-NMR (101 MHz, $CDCl_3$): δ 153.7, 144.5, 140.9, 127.8, 119.6, 105.7, 66.4, 61.1, 56.4, 45.8.

IR (ATR) $\tilde{\nu}$ (cm^{-1}): 3049, 2927, 1322, 1142, 1112, 942, 785.

MS (APCI): *m/z* calcd. for $C_{15}H_{22}NO_6S$ ($[M+H]^+$) 344.1, found 344.2.

HRMS (APCI): *m/z* calcd. for $C_{15}H_{22}NO_6S$ ($[M+H]^+$) 344.1162, found 344.1151.

Additional information on the chemical synthesis is available *via* Chemotion repository: https://doi.org/10.14272/reaction/SA-FUHFF-UHFFFADPSC-SXFSBPUXSG-UHFFFADPSC-NUHFF-NJZOK-NUHFF-ZZZ

Additional information on the analysis of the target compound is available *via* Chemotion repository:
https://doi.org/10.14272/SXFSBPUXSGAUHG-RUDMXATFSA-N.1

(*E*)-4-((2-(Naphthalen-1-yl)vinyl)sulfonyl)morpholine (7m)

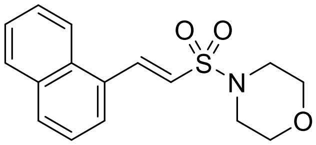

Following the **TP-4**, (*E*)-3-(naphthalen-1-yl)acrylic acid (59.5 mg, 0.3 mmol), morpholine (78.5 µL, 3.0 equiv.), and SO_2 stock solution (0.65 mL, 4.6 M in acetonitrile, 10.0 equiv.) were used as substrates. The residue was purified by flash chromatography over silica gel (*n*-hexane/EtOAc = 80:20) and recrystallized with EtOAc/*n*-hexane to obtain **7m** as a colorless solid (36.2 mg, 40% yield).

mp: 127.2–127.6 °C. R_f = 0.25 (*n*-hexane/EtOAc = 8:2).

^{1}H NMR (400 MHz, $CDCl_3$): δ 8.30 (d, *J* = 15.4 Hz, 1H), 8.10 (d, *J* = 8.2 Hz, 1H), 7.95 (d, *J* = 8.2 Hz, 1H), 7.90 (d, *J* = 7.6 Hz, 1H), 7.71 (d, *J* = 7.6 Hz, 1H), 7.64–7.59 (m, 1H), 7.59–7.54 (m, 1H), 7.51 (t, *J* = 7.6 Hz, 1H), 6.77 (d, *J* = 15.4 Hz, 1H), 3.85–3.77 (m, 4H), 3.30–3.23 (m, 4H).

^{13}C{^{1}H}-NMR (101 MHz, $CDCl_3$): δ 141.7, 133.8, 131.5, 131.3, 129.9, 129.0, 127.5, 126.7, 125.6, 125.5, 123.2, 123.1, 66.4, 45.9.

IR (ATR) $\tilde{\nu}$ (cm^{-1}): 3053, 2975, 1326, 1142, 1109, 946, 799.

MS (APCI): *m/z* calcd. for $C_{16}H_{18}NO_3S$ ($[M+H]^+$) 304.1, found 304.1.

HRMS (APCI): *m/z* calcd. for $C_{16}H_{18}NO_3S$ ($[M+H]^+$) 304.1002, found 304.0994.

Additional information on the chemical synthesis is available *via* Chemotion repository:
https://doi.org/10.14272/reaction/SA-FUHFF-UHFFFADPSC-MEOOGTHDWP-UHFFFADPSC-NUHFF-NRSGL-NUHFF-ZZZ

Additional information on the analysis of the target compound is available *via* Chemotion repository:
https://doi.org/10.14272/MEOOGTHDWPUJOE-MDWZMJQESA-N.1

(*E*)-4-((2-(Naphthalen-2-yl)vinyl)sulfonyl)morpholine (7n)

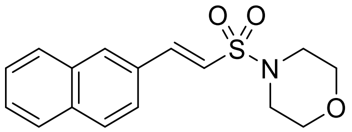

Following the **TP-4**, (*E*)-3-(naphthalen-2-yl)acrylic acid (59.5 mg, 0.3 mmol), morpholine (78.5 µL, 3.0 equiv.), and SO_2 stock solution (0.65 mL, 4.6 M in acetonitrile, 10.0 equiv.) were used as substrates. The residue was purified by flash chromatography over silica gel (*n*-hexane/EtOAc = 80:20) and recrystallized with EtOAc/*n*-hexane to obtain **7n** as a colorless solid (58.1 mg, 64% yield).

mp: 144.0–144.6 °C. R_f = 0.25 (*n*-hexane/EtOAc = 8:2).

^{1}H NMR (400 MHz, $CDCl_3$): δ 7.94 (s, 1H), 7.91–7.82 (m, 3H), 7.64 (d, *J* = 15.4 Hz, 1H), 7.62–7.52 (m, 3H), 6.78 (d, *J* = 15.4 Hz, 1H), 3.84–3.76 (m, 4H), 3.28–3.19 (m, 4H).

^{13}C{^{1}H}-NMR (101 MHz, $CDCl_3$): δ 144.5, 134.6, 133.3, 130.8, 129.9, 129.2, 128.8, 128.0, 127.9, 127.2, 123.3, 120.6, 66.4, 45.9.

IR (ATR) $\tilde{\nu}$ (cm^{-1}): 3053, 2972, 1326, 1144, 1108, 946, 800.

MS (APCI): *m/z* calcd. for $C_{16}H_{18}NO_3S$ ($[M+H]^+$) 304.1, found 304.1.

HRMS (APCI): *m/z* calcd. for $C_{16}H_{18}NO_3S$ ($[M+H]^+$) 304.1002, found 304.0993.

Additional information on the chemical synthesis is available *via* Chemotion repository:
https://doi.org/10.14272/reaction/SA-FUHFF-UHFFFADPSC-IDDPOLFDDL-UHFFFADPSC-NUHFF-NIDTW-NUHFF-ZZZ

Additional information on the analysis of the target compound is available *via* Chemotion repository:
https://doi.org/10.14272/IDDPOLFDDLUGNO-KPKJPENVSA-N.1

(*E*)-4-((2-(Furan-2-yl)vinyl)sulfonyl)morpholine (7o)

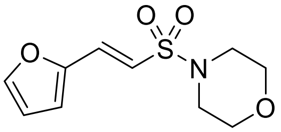

Following the **TP-4**, (*E*)-3-(furan-2-yl)acrylic acid (41.4 mg, 0.3 mmol), morpholine (78.5 µL, 3.0 equiv.), and SO_2 stock solution (0.65 mL, 4.6 M in acetonitrile, 10.0 equiv.) were used as substrates. The residue was purified by flash chromatography over silica gel (*n*-hexane/EtOAc = 85:15) and recrystallized with EtOAc/*n*-hexane to obtain **7o** as a colorless solid (33.6 mg, 46%).
mp: 101.8–102.1 °C. R_f = 0.20 (*n*-hexane/EtOAc = 8:2).
^{1}H NMR (400 MHz, $CDCl_3$): δ 7.55–7.50 (m, 1H), 7.23 (d, *J* = 15.2 Hz, 1H), 6.68 (d, *J* = 3.4 Hz, 1H), 6.54 (d, *J* = 15.2 Hz, 1H), 6.51 (dd, *J* = 3.4, 1.8 Hz, 1H), 3.81–3.75 (m, 4H), 3.21–3.15 (m, 4H).

^{13}C{^{1}H}-NMR (101 MHz, $CDCl_3$): δ 148.7, 145.5, 130.6, 117.9, 116.6, 112.7, 66.4, 45.8.

IR (ATR) $\tilde{v}$ (cm^{-1}): 3055, 2977, 1328, 1145, 1111, 941, 752.

MS (APCI): *m/z* calcd. for $C_{10}H_{14}NO_4S$ ($[M+H]^+$) 244.1, found 244.1.

HRMS (APCI): *m/z* calcd. for $C_{10}H_{14}NO_4S$ ($[M+H]^+$) 244.0638, found 244.0630.

Additional information on the chemical synthesis is available *via* Chemotion repository:
https://doi.org/10.14272/reaction/SA-FUHFF-UHFFFADPSC-JLTAKVTUDU-UHFFFADPSC-NUHFF-NCWIO-NUHFF-ZZZ
Additional information on the analysis of the target compound is available *via* Chemotion repository:
https://doi.org/10.14272/JLTAKVTUDUBOHD-YCRREMRBSA-N.1

(*E*)-4-((2-(Thiophen-2-yl)vinyl)sulfonyl)morpholine (7p)

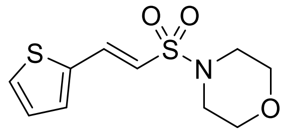

Following the **TP-4**, (*E*)-3-(thiophen-2-yl)acrylic acid (46.3 mg, 0.3 mmol), morpholine (78.5 µL, 3.0 equiv.), and SO_2 stock solution (0.65 mL, 4.6 M in acetonitrile, 10.0 equiv.) were used as substrates. The residue was purified by flash chromatography over silica gel (*n*-hexane/EtOAc = 85:15) and recrystallized with EtOAc/*n*-hexane to obtain **7p** as a colorless solid (38.8 mg, 50% yield).

mp: 141.0–141.4 °C. R_f = 0.20 (*n*-hexane/EtOAc = 8:2).

^{1}H NMR (400 MHz, $CDCl_3$): δ 7.58 (d, *J* = 15.2 Hz, 1H), 7.46 (d, *J* = 5.0 Hz, 1H), 7.30 (d, *J* = 3.6 Hz, 1H), 7.09 (dd, *J* = 5.0, 3.6 Hz, 1H), 6.46 (d, *J* = 15.2 Hz, 1H), 3.82–3.75 (m, 4H), 3.23–3.15 (m, 4H).

^{13}C{^{1}H}-NMR (101 MHz, $CDCl_3$): δ 137.0, 136.9, 132.3, 129.7, 128.5, 118.7, 66.4, 45.8.

IR (ATR) $\tilde{\nu}$ (cm^{-1}): 3040, 2975, 1326, 1144, 1111, 938, 712.

MS (APCI): *m/z* calcd. for $C_{10}H_{14}NO_3S_2$ ([M+H]$^+$) 260.0, found 260.0.

HRMS (APCI): *m/z* calcd. for $C_{10}H_{14}NO_3S_2$ ([M+H]$^+$) 260.0410, found 260.0406.

Additional information on the chemical synthesis is available *via* Chemotion repository:
https://doi.org/10.14272/reaction/SA-FUHFF-UHFFFADPSC-XBOHXHPLSM-UHFFFADPSC-NUHFF-NCWIO-NUHFF-ZZZ

Additional information on the analysis of the target compound is available *via* Chemotion repository:
https://doi.org/10.14272/XBOHXHPLSMXRNE-YCRREMRBSA-N.1

(*E*)-4-((2-Phenylprop-1-en-1-yl)sulfonyl)morpholine (7q)

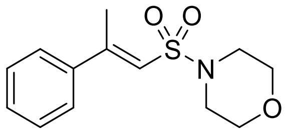

Following the **TP-4**, (*E*)-3-phenylbut-2-enoic acid (48.7 mg, 0.3 mmol), morpholine (78.5 µL, 3.0 equiv.), and SO_2 stock solution (0.65 mL, 4.6 M in acetonitrile, 10.0 equiv.) were used as substrates. The residue was purified by flash chromatography over silica gel (*n*-hexane/EtOAc = 80:20) and recrystallized with EtOAc/*n*-hexane to obtain **7q** as a colorless solid (48.3 mg, 60% yield).

mp: 99.3–99.8 °C. R_f = 0.25 (*n*-hexane/EtOAc = 8:2).

^{1}H NMR (400 MHz, $CDCl_3$): δ 7.48–7.37 (m, 5H), 6.31 (d, *J* = 1.2 Hz, 1H), 3.82–3.76 (m, 4H), 3.28–3.19 (m, 4H), 2.54 (d, *J* = 1.2 Hz, 3H).

^{13}C{^{1}H}-NMR (101 MHz, $CDCl_3$): δ 154.5, 140.8, 130.0, 129.0, 126.5, 120.4, 66.4, 45.9, 17.9.

IR (ATR) $\tilde{\nu}$ (cm^{-1}): 3065, 2917, 1341, 1152, 1111, 939, 763.

MS (APCI): *m/z* calcd. for $C_{13}H_{18}NO_3S$ ($[M+H]^+$) 268.1, found 268.0.

HRMS (APCI): *m/z* calcd. for $C_{13}H_{18}NO_3S$ ($[M+H]^+$) 268.1002, found 268.0992.

Additional information on the chemical synthesis is available *via* Chemotion repository: https://doi.org/10.14272/reaction/SA-FUHFF-UHFFFADPSC-VMTYEZAIFA-UHFFFADPSC-NUHFF-NZJBK-NUHFF-ZZZ

Additional information on the analysis of the target compound is available *via* Chemotion repository:

https://doi.org/10.14272/VMTYEZAIFAUVHJ-VAWYXSNFSA-N.1

5.3 Scale-up Reaction

The reaction was carried out using the divided glass cell with a P4 frit as described in section **1.2**.

Anolyte: In a 100 mL flask, a mixture of morpholine (2.62 mL, 30.0 mmol, 3.0 equiv.), $n\text{Bu}_4\text{NPF}_6$ (3.8745 g, 10.0 mmol, 1.0 equiv.), and DBU (6.43 g, 8.96 mL, 60.0 mmol, 6.0 equiv.) was prepared. The mixture was chilled to 0 °C in an ice bath, and SO_2 stock solution (18.4 mL, 5.44 M in acetonitrile, 100.0 mmol, 10.0 equiv.) was added, resulting in a yellow color solution. The mixture was diluted with acetonitrile (70.0 mL) so that a total volume of 100.0 mL was achieved.
Catholyte: A solution of $n\text{Bu}_4\text{NPF}_6$ (3.8745 g, 10.0 mmol, 1.0 equiv.) and acetic acid (2.86 mL, 50.0 mmol, 5.0 equiv.) in acetonitrile was prepared so that a total volume of 100.0 mL was reached as well.

The anodic compartment was charged with cinnamic acid (1.4820 g, 10.0 mmol), and the prepared electrolyte mixtures above were transferred to their respective compartments. The cell was equipped with a graphite anode and a graphite cathode. The current was set accordingly so that a current density of 15 $\text{mA}\times\text{cm}^{-2}$ was reached (45 mA with the setup described herein), and the amount of applied charge was set to 3377 C (corresponding to 3.5 *F*). The electrolysis was conducted at room temperature under constant stirring for 20 h 51 min.

After completion of the electrolysis, both the anolyte and catholyte were combined into a beaker and the compartments were rinsed with EtOAc (3 x 15 mL each). Water (200 mL) was added. Then the mixture was extracted with EtOAc (3 x 100 mL). The combined organic fractions were dried over $MgSO_4$, and the solvent was removed under reduced pressure. The crude residue was subjected to flash column chromatography on silica gel (*n*-hexane/EtOAc = 80:20) to obtain the impure product. The impure product was dissolved in EtOAc (100 mL) and heated in an oil bath at 90 ˚C. When the solution became turbid, *n*-hexane (20 mL) was added. After cooling the solution, the solid was filtered, and the desired product, (*E*)-4-(styrylsulfonyl)morpholine (**7a**, 1.917 g, 76%) was obtained as a colorless solid.

Spectroscopic data of the isolated compound matched the one obtained from the small-scale reaction described in section **5.2**.

Note: A ^{1}H NMR yield of 82% was calculated by the addition of triphenylmethane as an internal standard to the crude product before column chromatography.

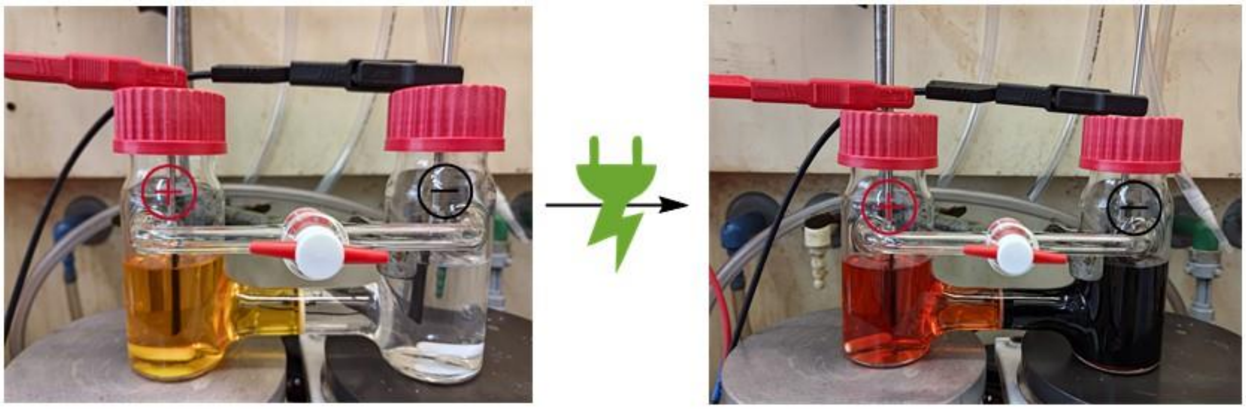

Figure S5: Scale-up experiment in a 2 x 100 mL glass cell before (left) and after (right) the electrolysis.

5.4 Reusability Tests

The reactions were carried out using IKA Pro-Divide cells with a P4 glass frit as described in the section before.

Anolyte: An oven-dried 10.0 mL pear-shaped flask was charged with cinnamic acid (44.5 mg, 0.3 mmol), morpholine (78.5 µL, 3.0 equiv.), nBu$_4$NPF$_6$ (116.3 mg, 1.0 equiv.), and anhydrous acetonitrile (2.0 mL). The mixture was cooled to 0 °C in an ice bath, followed by the addition of SO_2 stock solution (0.65 mL, 4.60 M in acetonitrile, 10.0 equiv.) and DBU (0.27 mL, 6.0 equiv.) so that a total volume of 3.0 mL was achieved.
Catholyte: An oven-dried 10.0 mL pear-shaped flask was charged with nBu$_4$NPF$_6$ (116.3 mg, 1.0 equiv.), acetic acid (0.09 mL, 5.0 equiv.), and anhydrous acetonitrile (2.9 mL) so that a total volume of 3.0 mL was achieved as well.

The reaction mixtures were transferred with syringes to their respective compartment simultaneously. The amperage was set accordingly so that a current density of 15 mA×cm^{-2} was reached (13.2 mA with the setup described herein), and the amount of applied charge was set to 3.5 *F*. The electrolysis was conducted at room temperature under constant stirring (400 rpm) for ca. 2 h 8 min. After completion of the electrolysis, the anolyte was transferred to a round-bottom

flask, and the anode compartment was rinsed with acetonitrile (2 x 3.0 mL). ^{1}H NMR yields were calculated by the addition of triphenylmethane (73.2 mg, 1.0 equiv.) as the internal standard to this mixture. After 5 min of stirring, 1.0 mL of the mixture was taken, and the solvent was removed under reduced pressure.

For the subsequent runs, the anolyte was prepared as described and transferred to the respective compartment. The graphite electrode, glass frit membrane, and catholyte (an additional amount of 0.07 mL acetic acid (4.0 equiv.) was added for each subsequent run to compensate for the loss from H_2 evolution from the previous experiment) were reused in the following experiments. The electrolysis and subsequent workup for determining ^{1}H NMR yield were conducted as described above. The complete procedure was conducted four times in total (**Figure S6**).

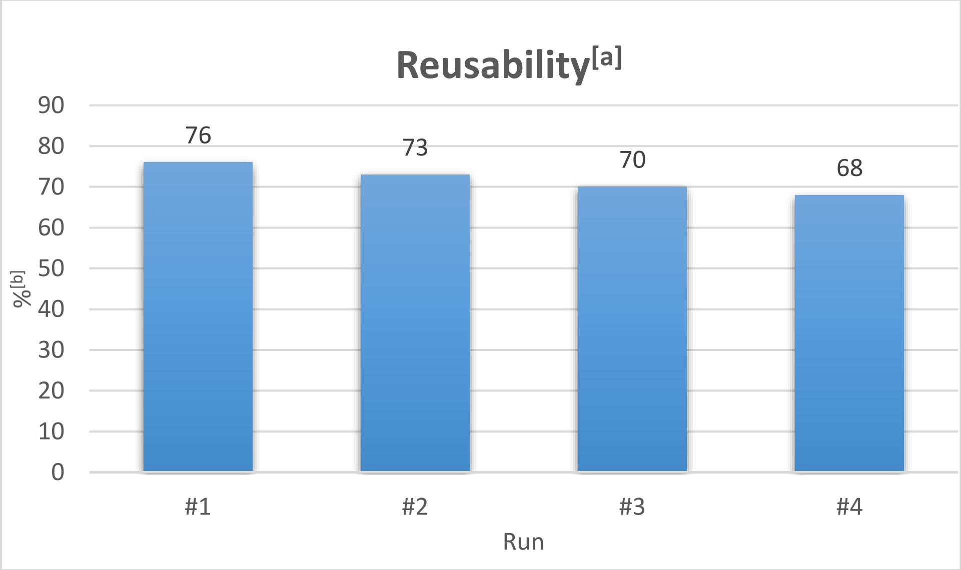

Figure S6: Tests for reusing the electrodes, glass frit membrane, and catholyte. [a]Conditions described above. [b]^{1}H-NMR yield with the use of $CHPh_3$ as the internal standard.

6 Synthesis of Alkyl Vinyl Sulfonamides [Scope of Amines]

6.1 Typical Procedure for the Preparation of Alkyl Vinyl Sulfonamides [Scope of Amines] (TP-5)

The reactions were carried out using IKA Pro-Divide cells with a P4 glass frit.

Anolyte: An oven-dried 10.0 mL pear-shaped flask was charged with cinnamic acid (44.5 mg, 0.3 mmol), amine (3.0 equiv.), *n*Bu_4NPF_6 (116.3 mg, 1.0 equiv.), and anhydrous acetonitrile. The mixture was cooled to 0 °C in an ice bath, followed by the addition of SO_2 stock solution (0.65 mL, 4.6 M in acetonitrile, 10.0 equiv.) and DBU (0.27 mL, 6.0 equiv.) so that a total volume of 3.0 mL was achieved. (For the synthesis of compound **8e**, DBU (9.0 equiv.) was used.)
Catholyte: An oven-dried 10.0 mL pear-shaped flask was charged with *n*Bu_4NPF_6 (116.3 mg, 1.0 equiv.), acetic acid (0.09 mL, 5.0 equiv.), and anhydrous acetonitrile (2.9 mL) so that a total volume of 3.0 mL was achieved as well.

The reaction mixtures were transferred with syringes to their respective compartment simultaneously. The amperage was set accordingly so that a current density of 15 $mA \times cm^{-2}$ was reached (13.2 mA with the setup described herein), and the amount of applied charge was set to 3.5 *F*. The electrolysis was conducted at room temperature under constant stirring (400 rpm) for ca. 2 h 8 min.

After completion of the electrolysis, the anolyte was transferred to a separatory funnel, and the anode compartment was rinsed with additional EtOAc (2 x 3.0 mL). Distilled water (10.0 mL) was added, and the obtained mixture was extracted with EtOAc (2 x 10.0 mL). The organic layer was dried over anhydrous Na_2SO_4 and concentrated in vacuo. The crude residue was subjected to flash column chromatography on silica gel to obtain the product.

For the synthesis of compounds **8k** and **8l**, amines (0.3 mmol) were the limiting reagents, and cinnamic acid (88.9 mg, 2.0 equiv.) was used.

6.2 Analytical Data for Compounds of Type 8 and 9

(*E*)-1-(Styrylsulfonyl)pyrrolidine (8a)

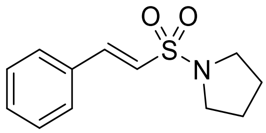

Following the **TP-5**, cinnamic acid (44.5 mg, 0.3 mmol), pyrrolidine (73.9 µL, 3.0 equiv.), and SO_2 stock solution (0.65 mL, 4.6 M in acetonitrile, 10.0 equiv.) were used as substrates. The residue was purified by flash chromatography over silica gel (*n*-hexane/EtOAc = 90:10) to obtain **8a** as a colorless solid (37.8 mg, 53% yield).

mp: 83.9–84.0 °C. R_f = 0.30 (*n*-hexane/EtOAc = 8:2).

^{1}H NMR (400 MHz, $CDCl_3$): δ 7.53–7.47 (m, 3H), 7.45–7.38 (m, 3H), 6.74 (d, *J* = 15.4 Hz, 1H), 3.37–3.30 (m, 4H), 1.94–1.88 (m, 4H).

^{13}C{^{1}H}-NMR (101 MHz, $CDCl_3$): δ 142.7, 132.9, 130.9, 129.2, 128.3, 120.9, 48.0, 25.8.

IR (ATR) $\tilde{\nu}$ (cm^{-1}): 3052, 2966, 1325, 1139, 963, 742.

MS (APCI): *m/z* calcd. for $C_{12}H_{16}NO_2S$ ($[M+H]^+$) 238.1, found 238.1.

HRMS (APCI): *m/z* calcd. for $C_{12}H_{16}NO_2S$ ($[M+H]^+$) 238.0896, found 238.0889.

Additional information on the chemical synthesis is available *via* Chemotion repository:
https://doi.org/10.14272/reaction/SA-FUHFF-UHFFFADPSC-VACRAHZRGQ-UHFFFADPSC-NUHFF-NFMKG-NUHFF-ZZZ

Additional information on the analysis of the target compound is available *via* Chemotion repository:
https://doi.org/10.14272/VACRAHZRGQJRFS-DHZHZOJOSA-N.1

(*E*)-1-(Styrylsulfonyl)piperidine (8b)

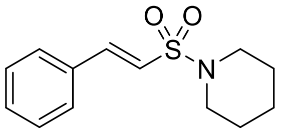

Following the **TP-5**, cinnamic acid (44.5 mg, 0.3 mmol), piperidine (88.9 µL, 3.0 equiv.), and SO_2 stock solution (0.65 mL, 4.6 M in acetonitrile, 10.0 equiv.) were used as substrates. The residue was purified by flash chromatography over silica gel (*n*-hexane/EtOAc = 90:10) to obtain **8b** as a colorless solid (40.9 mg, 54% yield).

mp: 89.1–89.2 °C. R_f = 0.40 (*n*-hexane/EtOAc = 8:2).

^{1}H NMR (400 MHz, $CDCl_3$): δ 7.53–7.48 (m, 2H), 7.48–7.38 (m, 4H), 6.69 (d, *J* = 15.4 Hz, 1H), 3.23–3.13 (m, 4H), 1.73–1.63 (m, 4H), 1.57–1.48 (m, 2H).

^{13}C{^{1}H}-NMR (101 MHz, $CDCl_3$): δ 142.9, 132.8, 130.9, 129.2, 128.3, 121.9, 46.7, 25.5, 23.8.

IR (ATR) $\tilde{\nu}$ (cm^{-1}): 3045, 2955, 1334, 1138, 933, 746.

MS (APCI): *m/z* calcd. for $C_{13}H_{18}NO_2S$ ($[M+H]^+$) 252.1, found 252.1.

HRMS (APCI): *m/z* calcd. for $C_{13}H_{18}NO_2S$ ($[M+H]^+$) 252.1053, found 252.1047.

Additional information on the chemical synthesis is available *via* Chemotion repository:
https://doi.org/10.14272/reaction/SA-FUHFF-UHFFFADPSC-FUDCYNIZRW-UHFFFADPSC-NUHFF-NODHV-NUHFF-ZZZ

Additional information on the analysis of the target compound is available *via* Chemotion repository:
https://doi.org/10.14272/FUDCYNIZRWWITL-FMIVXFBMSA-N.1

(*E*)-1-(Styrylsulfonyl)azepane (8c)

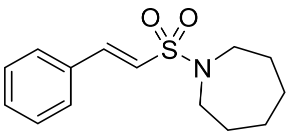

Following the **TP-5**, cinnamic acid (44.5 mg, 0.3 mmol), azepane (0.10 mL, 3.0 equiv.), and SO_2 stock solution (0.65 mL, 4.6 M in acetonitrile, 10.0 equiv.) were used as substrates. The residue was purified by flash chromatography over silica gel (*n*-hexane/EtOAc = 90:10) to obtain **8c** as a colorless solid (48.6 mg, 61% yield).

mp: 91.2–91.5 °C. R_f = 0.40 (*n*-hexane/EtOAc = 8:2).

^{1}H NMR (400 MHz, $CDCl_3$): δ 7.51–7.45 (m, 2H), 7.45–7.38 (m, 4H), 6.69 (d, *J* = 15.4 Hz, 1H), 3.39–3.31 (m, 4H), 1.82–1.70 (m, 4H), 1.68–1.59 (m, 4H).

^{13}C{^{1}H}-NMR (101 MHz, $CDCl_3$): δ 141.0, 133.0, 130.7, 129.1, 128.2, 123.8, 48.1, 29.5, 27.1.

IR (ATR) $\tilde{\nu}$ (cm^{-1}): 3045, 2953, 1334, 1138, 933, 745.

MS (APCI): *m/z* calcd. for $C_{14}H_{20}NO_2S$ ($[M+H]^+$) 266.1, found 266.1.

HRMS (APCI): *m/z* calcd. for $C_{14}H_{20}NO_2S$ ($[M+H]^+$) 266.1209, found 266.1205.

Additional information on the chemical synthesis is available *via* Chemotion repository:
https://doi.org/10.14272/reaction/SA-FUHFF-UHFFFADPSC-NRKAJPIDQI-UHFFFADPSC-NUHFF-NTYZU-NUHFF-ZZZ

Additional information on the analysis of the target compound is available *via* Chemotion repository:
https://doi.org/10.14272/NRKAJPIDQIITCE-JLHYYAGUSA-N.1

(*E*)-2-(Styrylsulfonyl)-1,2,3,4-tetrahydroisoquinoline (8d)

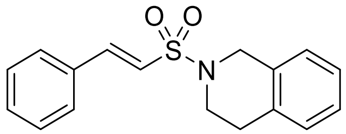

Following the **TP-5**, cinnamic acid (44.5 mg, 0.3 mmol), 1,2,3,4-tetrahydroisoquinoline (0.11 mL, 3.0 equiv.), and SO_2 stock solution (0.65 mL, 4.6 M in acetonitrile, 10.0 equiv.) were used as substrates. The residue was purified by flash chromatography over silica gel (*n*-hexane/EtOAc = 92:8) to obtain **8d** as a colorless solid (40.7 mg, 45% yield).

mp: 134.0–134.4 °C. R_f = 0.13 (*n*-hexane/EtOAc = 9:1).

^{1}H NMR (400 MHz, $CDCl_3$): δ 7.52 (d, *J* = 15.4 Hz, 1H), 7.47–7.35 (m, 5H), 7.20–7.14 (m, 2H), 7.14–7.04 (m, 2H), 6.67 (d, *J* = 15.4 Hz, 1H), 4.44 (s, 2H), 3.55 (t, *J* = 6.0 Hz, 2H), 2.96 (t, *J* = 6.0 Hz, 2H).

^{13}C{^{1}H}-NMR (101 MHz, $CDCl_3$): δ 143.2, 133.3, 132.7, 132.0, 131.0, 129.2, 129.1, 128.3, 127.0, 126.6, 126.4, 122.0, 47.3, 43.5, 28.8.

IR (ATR) $\tilde{\nu}$ (cm^{-1}): 3045, 2953, 1334, 1139, 933, 746.

MS (APCI): *m/z* calcd. for $C_{17}H_{18}NO_2S$ ($[M+H]^+$) 300.1, found 300.1.

HRMS (APCI): *m/z* calcd. for $C_{17}H_{18}NO_2S$ ($[M+H]^+$) 300.1053, found 300.1049.

Additional information on the chemical synthesis is available *via* Chemotion repository:
https://doi.org/10.14272/reaction/SA-FUHFF-UHFFFADPSC-SFHZTRWOSX-UHFFFADPSC-NUHFF-NCDQC-NUHFF-ZZZ

Additional information on the analysis of the target compound is available *via* Chemotion repository:
https://doi.org/10.14272/SFHZTRWOSXQCHP-ACCUITESSA-N.1

Methyl (*E*)-(styrylsulfonyl)-*L*-prolinate (8e)

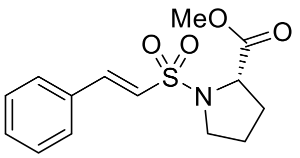

Following the **TP-5**, cinnamic acid (44.5 mg, 0.3 mmol), methyl *L*-prolinate hydrochloride (149.1 mg, 3.0 equiv.), and SO_2 stock solution (0.65 mL, 4.6 M in acetonitrile, 10.0 equiv.) were used as substrates. The residue was purified by flash chromatography over silica gel (*n*-hexane/EtOAc = 80:20) to obtain **8e** as a colorless liquid (35.0 mg, 40% yield).
R_f = 0.33 (*n*-hexane/EtOAc = 7:3).

^{1}H NMR (400 MHz, $CDCl_3$): δ 7.56–7.47 (m, 3H), 7.45–7.38 (m, 3H), 6.88 (d, *J* = 15.4 Hz, 1H), 4.45 (dd, *J* = 8.6, 3.2 Hz, 1H), 3.75 (s, 3H), 3.51–3.41 (m, 2H), 2.32–2.20 (m, 1H), 2.13–1.91 (m, 3H).
^{13}C{^{1}H}-NMR (101 MHz, $CDCl_3$): δ 173.0, 142.2, 132.9, 130.8, 129.2, 128.4, 123.8, 60.6, 52.6, 47.9, 31.2, 24.9.

IR (ATR) $\tilde{\nu}$ (cm^{-1}): 3053, 2955, 1740, 1332, 1141, 976, 745.

MS (APCI): *m/z* calcd. for $C_{14}H_{18}NO_4S$ ($[M+H]^+$) 296.1, found 296.1.

HRMS (APCI): *m/z* calcd. for $C_{14}H_{18}NO_4S$ ($[M+H]^+$) 296.0951, found 296.0947.

Additional information on the chemical synthesis is available *via* Chemotion repository:
https://doi.org/10.14272/reaction/SA-FUHFF-UHFFFADPSC-IZUNNGXPNJ-UHFFFADPSC-NUHFF-NGFUR-NUHFF-ZZZ

Additional information on the analysis of the target compound is available *via* Chemotion repository:
https://doi.org/10.14272/IZUNNGXPNJQHQH-STRFDMGBSA-N.1

tert-Butyl (E)-4-(styrylsulfonyl)piperazine-1-carboxylate (8f)

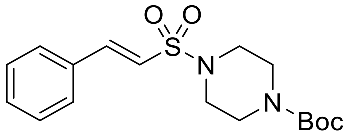

Following the **TP-5**, cinnamic acid (44.5 mg, 0.3 mmol), *tert*-butyl piperazine-1-carboxylate (167.6 mg, 3.0 equiv.), and SO_2 stock solution (0.65 mL, 4.6 M in acetonitrile, 10.0 equiv.) were used as substrates. The residue was purified by flash chromatography over silica gel (*n*-hexane/EtOAc = 85:15) to obtain **8f** as a colorless solid (65.3 mg, 62% yield).
mp: 118.8–119.0 °C. R_f = 0.17 (*n*-hexane/EtOAc = 8:2).

^{1}H NMR (400 MHz, $CDCl_3$): δ 7.54–7.38 (m, 6H), 6.67 (d, *J* = 15.4 Hz, 1H), 3.58–3.50 (m, *J* = 3.8 Hz, 4H), 3.22–3.12 (m, 4H), 1.44 (s, 9H).
^{13}C{^{1}H}-NMR (101 MHz, $CDCl_3$): δ 154.3, 144.2, 132.5, 131.3, 129.3, 128.4, 120.9, 80.6, 45.7, 43.3, 28.4.

IR (ATR) $\tilde{\nu}$ (cm^{-1}): 2976, 1684, 1322, 1172, 1142, 942, 743.

MS (APCI): *m/z* calcd. for $C_{17}H_{25}N_2O_4S$ ($[M+H]^+$) 353.1, found 353.2.

HRMS (ESI^+): *m/z* calcd. for $C_{17}H_{24}N_2O_4SNa$ ($[M+Na]^+$) 375.1349, found 375.1341.

Additional information on the chemical synthesis is available *via* Chemotion repository:
https://doi.org/10.14272/reaction/SA-FUHFF-UHFFFADPSC-UOORUKALEH-UHFFFADPSC-NUHFF-NUEKX-NUHFF-ZZZ
Additional information on the analysis of the target compound is available *via* Chemotion repository:
https://doi.org/10.14272/UOORUKALEHQNON-NTEUORMPSA-N.1

(*E*)-2-Phenyl-*N*,*N*-dipropylethene-1-sulfonamide (8g)

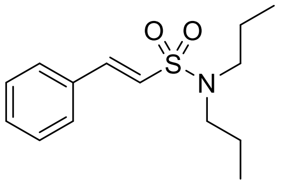

Following the **TP-5**, cinnamic acid (44.5 mg, 0.3 mmol), *N*-propylpropan-1-amine (0.12 mL, 3.0 equiv.), and SO_2 stock solution (0.65 mL, 4.6 M in acetonitrile, 10.0 equiv.) were used as substrates. The residue was purified by flash chromatography over silica gel (*n*-hexane/EtOAc = 94:6) to obtain **8g** as a colorless liquid (30.7 mg, 38% yield).

R_f = 0.23 (*n*-hexane/EtOAc = 9:1).

^{1}H NMR (400 MHz, $CDCl_3$): δ 7.51–7.46 (m, 2H), 7.46–7.38 (m, 4H), 6.67 (d, *J* = 15.4 Hz, 1H), 3.17–3.09 (m, 4H), 1.70–1.58 (m, 4H), 0.92 (t, *J* = 7.4 Hz, 6H).

^{13}C{^{1}H}-NMR (101 MHz, $CDCl_3$): δ 140.9, 133.1, 130.7, 129.2, 128.2, 124.6, 49.8, 22.4, 11.3.

IR (ATR) $\tilde{\nu}$ (cm^{-1}): 3062, 2965, 1334, 1138, 975, 745.

MS (APCI): *m/z* calcd. for $C_{14}H_{22}NO_2S$ ($[M+H]^+$) 268.1, found 268.1.

HRMS (APCI): *m/z* calcd. for $C_{14}H_{22}NO_2S$ ($[M+H]^+$) 268.1366, found 268.1358.

Additional information on the chemical synthesis is available *via* Chemotion repository:
https://doi.org/10.14272/reaction/SA-FUHFF-UHFFFADPSC-VKDODRXIDY-UHFFFADPSC-NUHFF-NTYZU-NUHFF-ZZZ

Additional information on the analysis of the target compound is available *via* Chemotion repository:
https://doi.org/10.14272/VKDODRXIDYKVIT-JLHYYAGUSA-N.1

(*E*)-*N*,*N*-Diisobutyl-2-phenylethene-1-sulfonamide (8h)

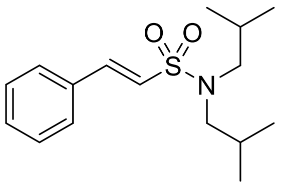

Following the **TP-5**, cinnamic acid (44.5 mg, 0.3 mmol), diisobutylamine (0.16 mL, 3.0 equiv.), and SO_2 stock solution (0.65 mL, 4.6 M in acetonitrile, 10.0 equiv.) were used as substrates. The residue was purified by flash chromatography over silica gel (*n*-hexane/EtOAc = 96:4) to obtain **8h** as a colorless solid (27.9 mg, 31% yield).

mp: 85.1–85.4 °C. R_f = 0.30 (*n*-hexane/EtOAc = 9:1).

^{1}H NMR (400 MHz, $CDCl_3$): δ 7.50–7.46 (m, 2H), 7.46–7.38 (m, 4H), 6.69 (d, *J* = 15.4 Hz, 1H), 2.96 (d, *J* = 7.6 Hz, 4H), 2.03–1.87 (m, 2H), 0.95 (d, *J* = 6.6 Hz, 12H).

^{13}C{^{1}H}-NMR (101 MHz, $CDCl_3$): δ 141.1, 133.1, 130.7, 129.2, 128.2, 124.3, 56.6, 27.4, 20.3.

IR (ATR) $\tilde{\nu}$ (cm^{-1}): 3057, 2957, 1338, 1148, 976, 746.

MS (APCI): *m/z* calcd. for $C_{16}H_{26}NO_2S$ ([M+H]$^+$) 296.2, found 296.2.

HRMS (APCI): *m/z* calcd. for $C_{16}H_{26}NO_2S$ ([M+H]$^+$) 296.1679, found 296.1671.

Additional information on the chemical synthesis is available *via* Chemotion repository:
https://doi.org/10.14272/reaction/SA-FUHFF-UHFFFADPSC-HPKBZGHOZY-UHFFFADPSC-NUHFF-NZAEC-NUHFF-ZZZ

Additional information on the analysis of the target compound is available *via* Chemotion repository:
https://doi.org/10.14272/HPKBZGHOZYVBOU-ZHACJKMWSA-N.1

(*E*)-*N*,*N*-Dibenzyl-2-phenylethene-1-sulfonamide (8i)

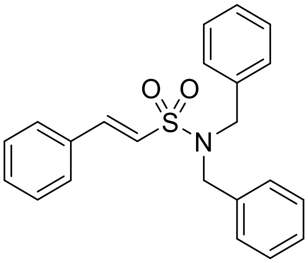

Following the **TP-5**, cinnamic acid (44.5 mg, 0.3 mmol), dibenzylamine (0.17 mL, 3.0 equiv.), and SO_2 stock solution (0.65 mL, 4.6 M in acetonitrile, 10.0 equiv.) were used as substrates. The residue was purified by flash chromatography over silica gel (*n*-hexane/EtOAc = 97:3) to obtain **8i** as a colorless solid (51.6 mg, 47% yield).

mp: 103.0–103.5 °C. R_f = 0.38 (*n*-hexane/EtOAc = 9:1).

^{1}H NMR (400 MHz, $CDCl_3$): δ 7.42 (d, *J* = 15.4 Hz, 1H), 7.40–7.26 (m, 15H), 6.50 (d, *J* = 15.4 Hz, 1H), 4.33 (s, 4H).

^{13}C{^{1}H}-NMR (101 MHz, $CDCl_3$): δ 141.2, 135.8, 132.8, 130.8, 129.1, 128.9, 128.8, 128.2, 128.1, 125.5, 50.0.

IR (ATR) $\tilde{\nu}$ (cm^{-1}): 3055, 1322, 1142, 978, 738.

MS (APCI): *m/z* calcd. for $C_{22}H_{22}NO_2S$ ($[M+H]^+$) 364.1, found 364.2.

HRMS (APCI): *m/z* calcd. for $C_{22}H_{22}NO_2S$ ($[M+H]^+$) 364.1366, found 364.1359.

Additional information on the chemical synthesis is available *via* Chemotion repository:
https://doi.org/10.14272/reaction/SA-FUHFF-UHFFFADPSC-QJUZBZYFIG-UHFFFADPSC-NUHFF-NAKRX-NUHFF-ZZZ

Additional information on the analysis of the target compound is available *via* Chemotion repository:
https://doi.org/10.14272/QJUZBZYFIGLQCL-WUKNDPDISA-N.1

(*E*)-5-(Styrylsulfonyl)-4,5,6,7-tetrahydrothieno[3,2-*c*]pyridine (8j)

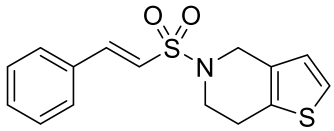

Following the **TP-5**, cinnamic acid (44.5 mg, 0.3 mmol), 4,5,6,7-tetrahydrothieno[3,2-*c*]pyridine hydrochloride (158.1 mg, 3.0 equiv.), and SO_2 stock solution (0.65 mL, 4.6 M in acetonitrile, 10.0 equiv.) were used as substrates. The residue was purified by flash chromatography over silica gel (*n*-hexane/EtOAc = 90:10) to obtain **8j** as a colorless solid (8.8 mg, 10% yield).

mp: 97.5–98.1 °C. R_f = 0.43 (*n*-hexane/EtOAc = 8:2).

^{1}H NMR (400 MHz, $CDCl_3$): δ 7.52 (d, *J* = 15.4 Hz, 1H), 7.47–7.36 (m, 5H), 7.14 (d, *J* = 5.2 Hz, 1H), 6.77 (d, *J* = 5.2 Hz, 1H), 6.66 (d, *J* = 15.4 Hz, 1H), 4.42–4.36 (m, 2H), 3.63 (t, *J* = 5.8 Hz, 2H), 2.97 (t, *J* = 5.8 Hz, 2H).

^{13}C{^{1}H}-NMR (101 MHz, $CDCl_3$): δ 143.0, 132.9, 132.7, 131.09, 131.05, 129.2, 128.4, 124.8, 123.9, 122.4, 45.7, 43.7, 25.2.

IR (ATR) $\tilde{\nu}$ (cm^{-1}): 3063, 2916, 1321, 1142, 985, 740.

MS (APCI): *m/z* calcd. for $C_{15}H_{16}NO_2S_2$ ($[M+H]^+$) 306.1, found 305.9.

HRMS (APCI): *m/z* calcd. for $C_{15}H_{16}NO_2S_2$ ($[M+H]^+$) 306.0617, found 306.0612.

Additional information on the chemical synthesis is available *via* Chemotion repository:
https://doi.org/10.14272/reaction/SA-FUHFF-UHFFFADPSC-BYSFXXZWOR-UHFFFADPSC-NUHFF-NFMKG-NUHFF-ZZZ

Additional information on the analysis of the target compound is available *via* Chemotion repository:
https://doi.org/10.14272/BYSFXXZWORGIDO-DHZHZOJOSA-N.1

(*R*,*E*)-*N*-(1-(Naphthalen-1-yl)ethyl)-2-phenyl-*N*-(3-(3-(trifluoromethyl)phenyl)propyl)ethene-1-sulfonamide (8k)

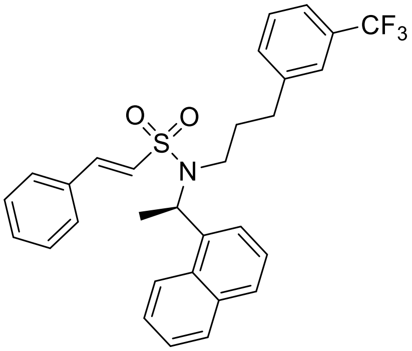

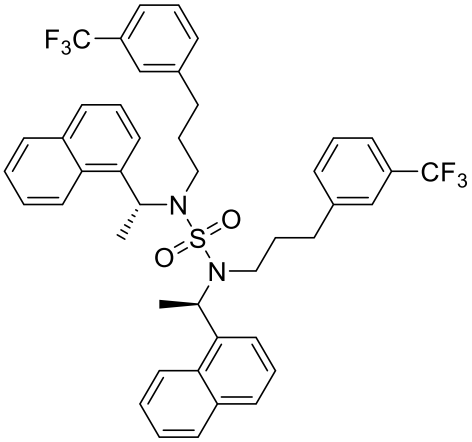

Following the **TP-5**, (*R*)-*N*-(1-(naphthalen-1-yl)ethyl)-3-(3-(trifluoromethyl)phenyl)propan-1-amine (107.2 mg, 0.3 mmol), cinnamic acid (88.9 mg, 2.0 equiv.), and SO_2 stock solution (0.65 mL, 4.6 M in acetonitrile, 10.0 equiv.) were used as substrates. The residue was purified by flash chromatography over silica gel (*n*-hexane/EtOAc = 95:5) to obtain **8k** as a colorless solid (47.4 mg, 30% yield) together with the side product **9k** (33.4 mg, 0.04 mmol, colorless solid).

Analytica data for **8k**:

mp: 85.7–86.1 °C. R_f = 0.30 (*n*-hexane/EtOAc = 9:1).

^{1}H NMR (400 MHz, $CDCl_3$): δ 8.57 (d, *J* = 8.4 Hz, 1H), 7.87 (d, *J* = 8.0 Hz, 1H), 7.83 (d, *J* = 7.6 Hz, 1H), 7.64–7.58 (m, 1H), 7.55–7.51 (m, 1H), 7.50 (d, *J* = 15.4 Hz, 1H), 7.47–7.31 (m, 8H), 7.20 (t, *J* = 7.6 Hz, 1H), 6.84 (s, 1H), 6.79 (d, *J* = 7.6 Hz, 1H), 6.72 (d, *J* = 15.4 Hz, 1H), 6.09 (q, *J* = 6.8 Hz, 1H), 3.06–2.83 (m, 2H), 2.11 (t, *J* = 7.8 Hz, 2H), 1.71 (d, *J* = 6.8 Hz, 3H), 1.37–1.21 (m, 1H), 0.82–0.65 (m, 1H).

^{13}C{^{1}H}-NMR (101 MHz, $CDCl_3$): δ 142.1, 141.6, 135.1, 133.9, 132.9, 132.1, 131.6, 130.9, 130.4 (q, $^2J_{C\text{-}F}$ = 32.0 Hz, 1C), 129.5, 129.2, 128.9, 128.6, 128.3, 127.1, 126.3, 125.3, 124.93, 124.88 (q, $^3J_{C\text{-}F}$ = 3.8 Hz, 1C), 124.5, 124.3 (q, $^1J_{C\text{-}F}$ = 272.6 Hz, 1C), 124.2, 122.6 (q, $^3J_{C\text{-}F}$ = 4.0 Hz, 1C), 52.4, 43.0, 32.8, 31.5, 17.5.

^{19}F{^{1}H}-NMR (376 MHz, $CDCl_3$): δ -62.4.

IR (ATR) $\tilde{\nu}$ (cm^{-1}): 3053, 2937, 1325, 1118, 975, 780

MS (APCI): *m/z* calcd. for $C_{30}H_{29}NO_2F_3S$ ($[M+H]^+$) 524.2, found 524.2.

HRMS (APCI): *m/z* calcd. for $C_{30}H_{29}NO_2F_3S$ ($[M+H]^+$) 524.1866, found 524.1857.

Analytica data for **9k**:

mp: 130.0–130.6 °C. R_f = 0.40 (*n*-hexane/EtOAc = 9:1).

^{1}H NMR (400 MHz, $CDCl_3$): δ 8.61 (d, *J* = 8.6 Hz, 2H), 7.89 (t, *J* = 7.4 Hz, 4H), 7.72–7.64 (m, 2H), 7.59–7.51 (m, 4H), 7.41 (t, *J* = 7.8 Hz, 2H), 7.31 (d, *J* = 7.8 Hz, 2H), 7.18 (t, *J* = 7.8 Hz, 2H), 6.78–6.69 (m, 4H), 6.24 (q, *J* = 6.8 Hz, 2H), 3.61–3.45 (m, 2H), 3.22–3.09 (m, 2H), 2.15–2.03 (m, 4H), 2.01 (d, *J* = 6.8 Hz, 6H), 1.18–1.03 (m, 2H), 0.44–0.28 (m, 2H).

^{13}C{^{1}H}-NMR (101 MHz, $CDCl_3$): δ 141.9, 134.1, 133.9, 132.1, 131.6, 130.5 (q, $^{2}J_{C\text{-}F}$ = 32.0 Hz, 2C), 130.1, 129.1, 128.6, 127.5, 126.5, 125.1, 125.0, 124.8 (q, $^{3}J_{C\text{-}F}$ = 3.6 Hz, 2C), 124.3 (q, $^{1}J_{C\text{-}F}$ = 272.8 Hz, 2C), 123.7, 122.7 (q, $^{3}J_{C\text{-}F}$ = 3.8 Hz, 2C), 54.8, 45.1, 32.7, 31.5, 17.0.

^{19}F{^{1}H}-NMR (376 MHz, $CDCl_3$): δ -62.3.

IR (ATR) $\tilde{\nu}$ (cm^{-1}): 3059, 2947, 1329, 1154, 780.

MS (APCI): *m/z* calcd. for $C_{44}H_{43}N_2O_2F_6S$ ($[M+H]^+$) 777.3, found 777.3.

HRMS (APCI): *m/z* calcd. for $C_{44}H_{43}N_2O_2F_6S$ ($[M+H]^+$) 777.2944, found 777.2928.

Additional information on the chemical synthesis is available *via* Chemotion repository:
https://doi.org/10.14272/reaction/SA-FUHFF-UHFFFADPSC-ZJZFIUBSVU-UHFFFADPSC-NUHFF-NGXNO-NUHFF-ZZZ

Additional information on the analysis of the target compound is available *via* Chemotion repository:
https://doi.org/10.14272/NTKCJMMFIJZYOO-LBMBTHBCSA-N.1
https://doi.org/10.14272/PYXXEMGUOSMROK-UHFFFAOYSA-N.1

(3*S*,4*R*)-3-((Benzo[*d*][1,3]dioxol-5-yloxy)methyl)-4-(4-fluorophenyl)-1-(((*E*)-styryl)sulfonyl)piperidine (8l)

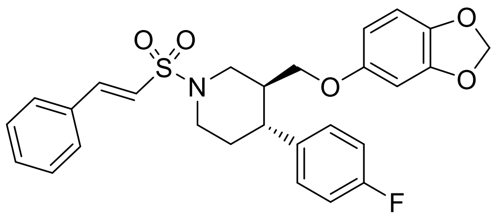

Following the **TP-5**, (3*S*,4*R*)-3-((benzo[*d*][1,3]dioxol-5-yloxy)methyl)-4-(4-fluorophenyl)piperidine (98.8 mg, 0.3 mmol), cinnamic acid (88.9 mg, 2.0 equiv.), and SO_2 stock solution (0.65 mL, 4.6 M in acetonitrile, 10.0 equiv.) were used as substrates. The residue was purified by flash chromatography over silica gel (*n*-hexane/EtOAc = 90:10) to obtain **8l** as a colorless solid (7.5 mg, 5% yield).

mp: 138.3–138.8 °C. R_f = 0.40 (*n*-hexane/EtOAc = 8:2).

^{1}H NMR (400 MHz, $CDCl_3$): δ 7.56–7.48 (m, 3H), 7.48–7.40 (m, 3H), 7.17–7.10 (m, 2H), 7.03–6.95 (m, 2H), 6.74 (d, *J* = 15.4 Hz, 1H), 6.62 (d, *J* = 8.4 Hz, 1H), 6.34 (d, *J* = 2.6 Hz, 1H), 6.12 (dd, *J* = 8.4, 2.6 Hz, 1H), 5.91–5.85 (m, 2H), 4.16–4.08 (m, 1H), 4.00–3.90 (m, 1H), 3.67–3.58 (m, 1H), 3.50–3.40 (m, 1H), 2.83–2.69 (m, 2H), 2.69–2.57 (m, 1H), 2.30–2.19 (m, 1H), 2.03–1.89 (m, 2H).

^{13}C{^{1}H}-NMR (101 MHz, $CDCl_3$): δ 161.9 (d, $^1J_{C-F}$ = 245.2 Hz, 1C), 154.1, 148.3, 143.6, 142.0, 138.4 (d, $^4J_{C-F}$ = 3.2 Hz, 1C), 132.7, 131.1, 129.3, 128.9 (d, $^3J_{C-F}$ = 7.8 Hz, 2C), 128.5, 121.6, 115.8 (d, $^2J_{C-F}$ = 21.2 Hz, 2C), 108.0, 105.6, 101.3, 98.1, 68.6, 49.3, 46.5, 43.4, 42.1, 33.6.

^{19}F{^{1}H}-NMR (376 MHz, $CDCl_3$): δ -115.6.

IR (ATR) $\tilde{\nu}$ (cm^{-1}): 3050, 2920, 1487, 1335, 1181, 1146, 975, 783.

MS (APCI): *m/z* calcd. for $C_{27}H_{27}NO_5FS$ ($[M+H]^+$) 496.1, found 496.0.

HRMS (APCI): *m/z* calcd. for $C_{27}H_{27}NO_5FS$ ($[M+H]^+$) 496.1588, found 496.1574.

Additional information on the chemical synthesis is available *via* Chemotion repository: https://doi.org/10.14272/reaction/SA-FUHFF-UHFFFADPSC-LPWHFWMTRK-UHFFFADPSC-NUHFF-NISMK-NUHFF-ZZZ

Additional information on the analysis of the target compound is available *via* Chemotion

repository:

https://doi.org/10.14272/LPWHFWMTRKYSIY-XEJDZBDASA-N.1

7 Unsuccessful Substrates

Cinnamic acids and related substrates:

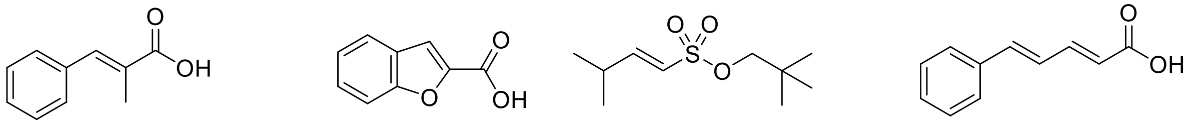

Traces amount of product was detected

No alkenesulfonamide product was detected

Amines:

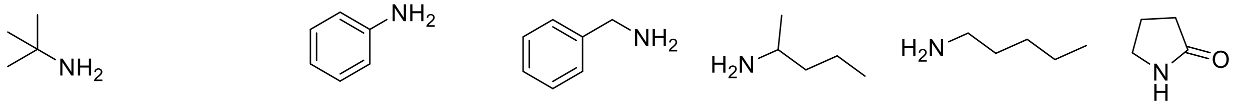

Too sterically demanding

Oxidation of aniline

No alkenesulfonamide product was detected

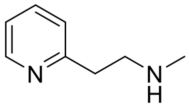

Traces amount of product was detected

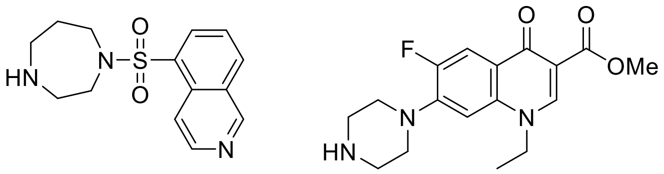

No alkenesulfonamide product was detected

Figure S7: Unsuccessful derivatives under the standard conditions.

8 Control Experiments

The control experiments were performed to elucidate the reaction mechanism. As shown in **Table S3**, only trace amounts of products were observed in the absence of a base, and no reaction took place without applying an electric current (Entries 2 and 3). Trace amounts of products were detected in the crude reaction mixture when the radical scavengers, such as 2,6-di-*tert*-butyl-4-methylphenol (BHT) or 2,2,6,6-tetramethylpiperidinyl-oxyl (TEMPO), were added. In both cases, only partial decomposition of cinnamic acid **5a** was observed.

Table S3: Control experiments.

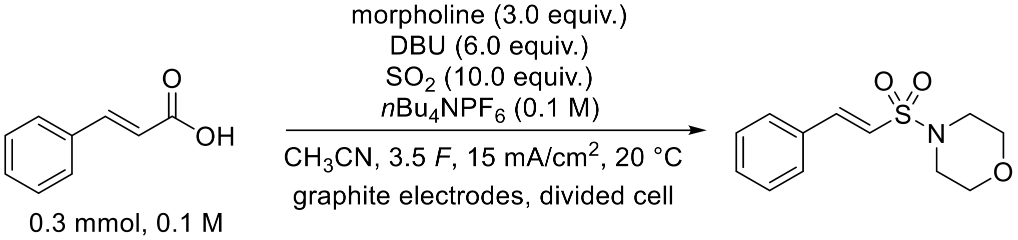

Entry	Deviation from the standard conditions	Yield (%)[a]	Cinnamic acid (%)[a]
**1**	**None**	**76 (73)**[b]	**0**
2	No DBU	Traces	12
3	No electric current	0	90
4	+BHT (3.0 equiv.)	5	63
5	+TEMPO (3.0 equiv.)	12	86

[a] ^{1}H-NMR yield with the use of $CHPh_3$ as the internal standard. [b] Isolated yield.

9 Cyclic Voltammetry Results

The following measurements were used for the postulation of the reaction mechanism: the oxidation of the cinnamate **II** should take place before the oxidation of the amidosulfinate intermediate **I** (**Figure S8**, Measurement conditions: 0.1 M *n*Bu$_4$NPF$_6$ in CH_3CN; *c*(cinnamic acid **5a**, morpholine, DBU, or **7a**) = 0.01 M; *c*(SO_2) = 0.05 M; *c*(DBU in **I** and **II**) = 0.011 M; v = 100 mV/s.).

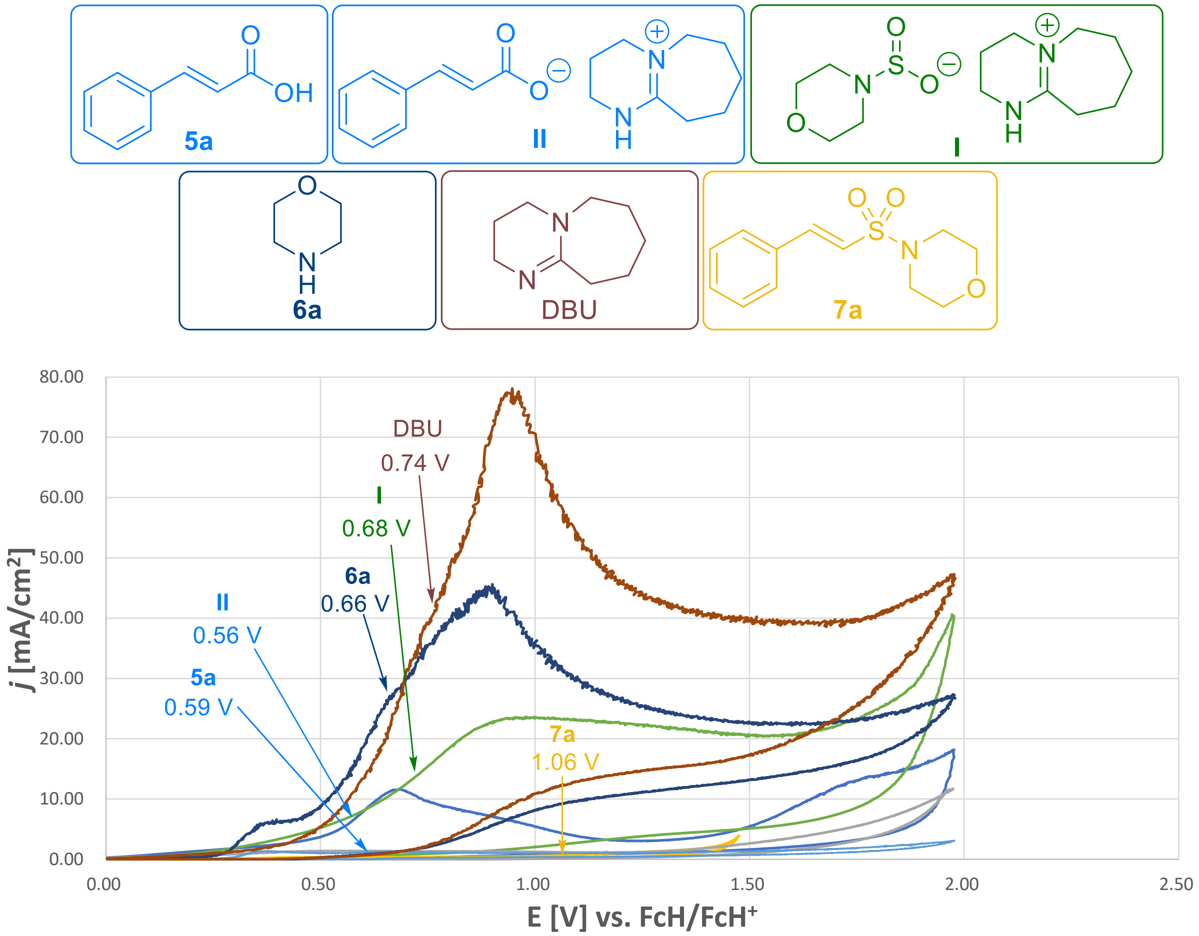

Figure S8: Cyclic voltammograms of cinnamate **II** (blue), cinnamic acid **5a** (blue), morpholine **6a** (dark blue), amidosulfinate intermediate **I** (green), DBU (brown), vinyl sulfonamide **7a** (yellow), and blank measurement (0.1 M *n*Bu$_4$NPF$_6$ in CH_3CN; grey).

Cinnamic acid **5a** and cinnamate **II** exhibit similar oxidation potentials. The addition of DBU exerts no noticeable effect on the oxidation potential of cinnamate **II** (**Figure S9**, Measurement conditions: 0.1 M nBu$_4$NPF$_6$ in CH$_3$CN; c(cinnamic acid **5a** and DBU) = 0.01 M; c(DBU in **II**) = 0.011 M; v = 100 mV/s.).

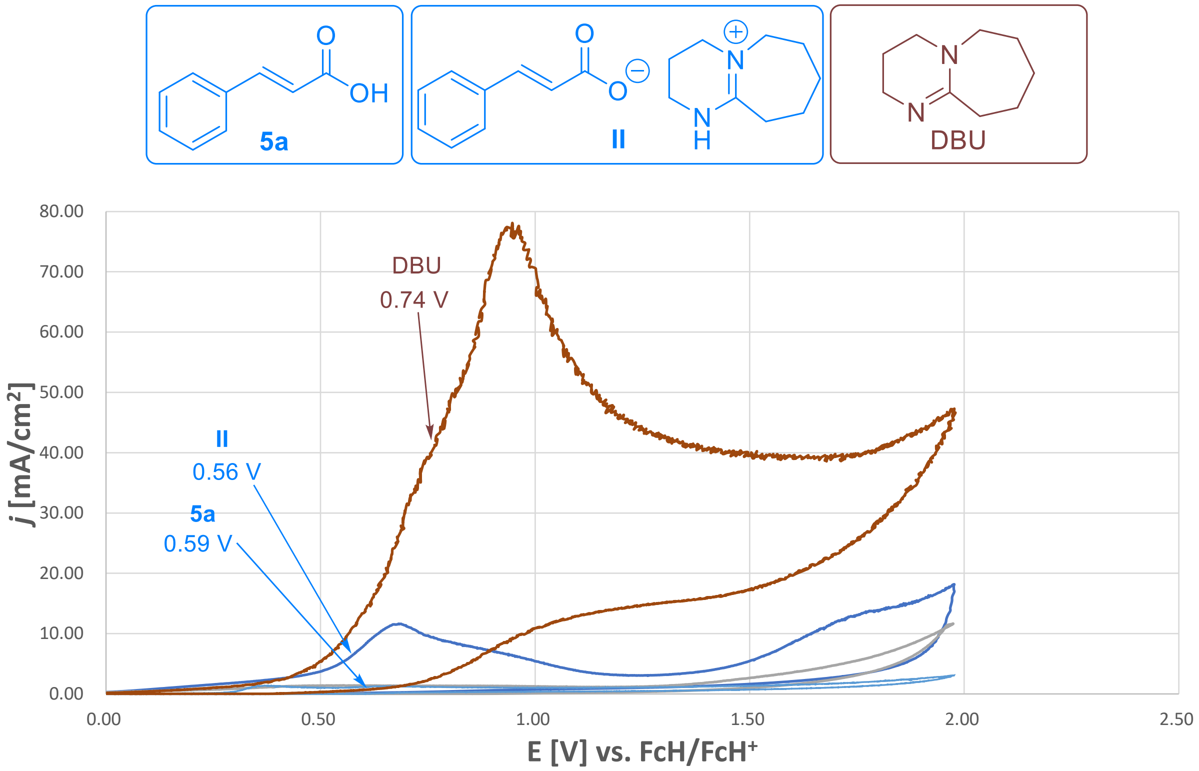

Figure S9: Cyclic voltammograms of cinnamate **II** (blue), cinnamic acid **5a** (blue), DBU (brown), and blank measurement (0.1 M nBu$_4$NPF$_6$ in CH$_3$CN; grey).

Sulfur dioxide reacts with morpholine **6a** to form Lewis acid–base adducts, which equilibrate to amidosulfinate intermediate **I** following deprotonation by DBU as an organic base.[35] Notably, the presence of DBU does not significantly influence the oxidation potential of the amidosulfinate intermediate **I** (**Figure S10**, Measurement conditions: 0.1 M $n\mathrm{Bu_4NPF_6}$ in CH_3CN; *c*(morpholine and DBU) = 0.01 M; *c*(SO_2) = 0.05 M; *c*(DBU in **I**) = 0.011 M; v = 100 mV/s.).

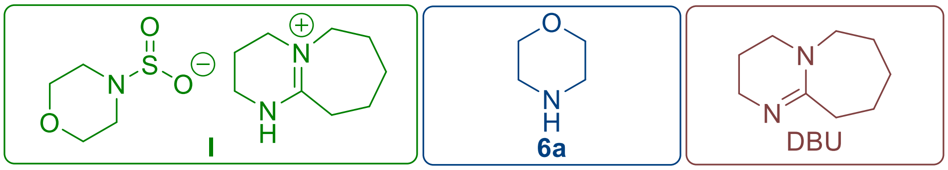

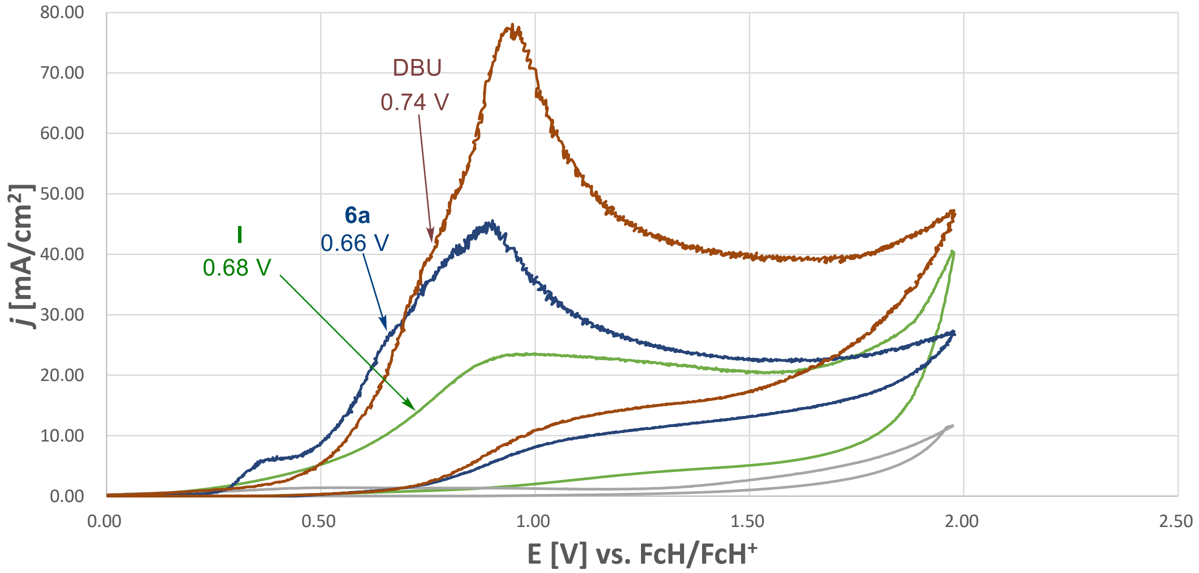

Figure S10: Cyclic voltammograms of morpholine **6a** (dark blue), amidosulfinate intermediate **I** (green), DBU (brown), and blank measurement (0.1 M $n\mathrm{Bu_4NPF_6}$ in CH_3CN; grey).

In the case of cinnamate **II**, the measured current decreases significantly with the number of CV cycles. This indicates the possible formation of a polymeric layer and adsorption on the surface of the WE.[30] (**Figure S11**, Measurement conditions: 0.1 M nBu$_4$NPF$_6$ in CH_3CN; c(cinnamic acid **5a**) = 0.01 M; c(DBU) = 0.011 M; v = 100 mV/s.).

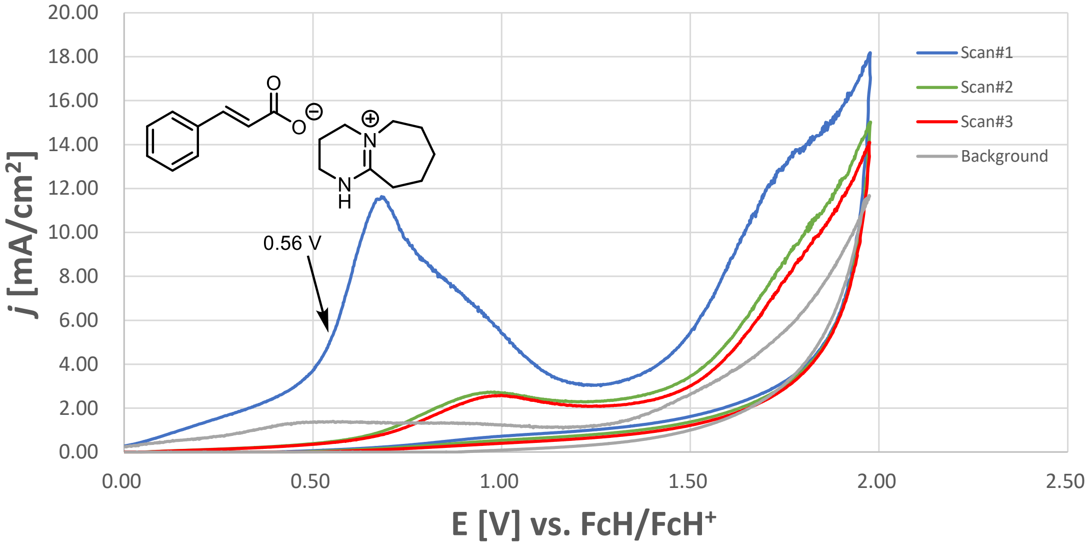

Figure S11: Cyclic voltammograms of cinnamate **II**.

There is no obvious current change in the number of scans when measuring the product **7a**. Thus, overoxidation of the product should be negligible. (**Figure S12**, Measurement conditions: 0.1 M nBu$_4$NPF$_6$ in CH_3CN; c(**7a**) = 0.01 M; v = 100 mV/s.).

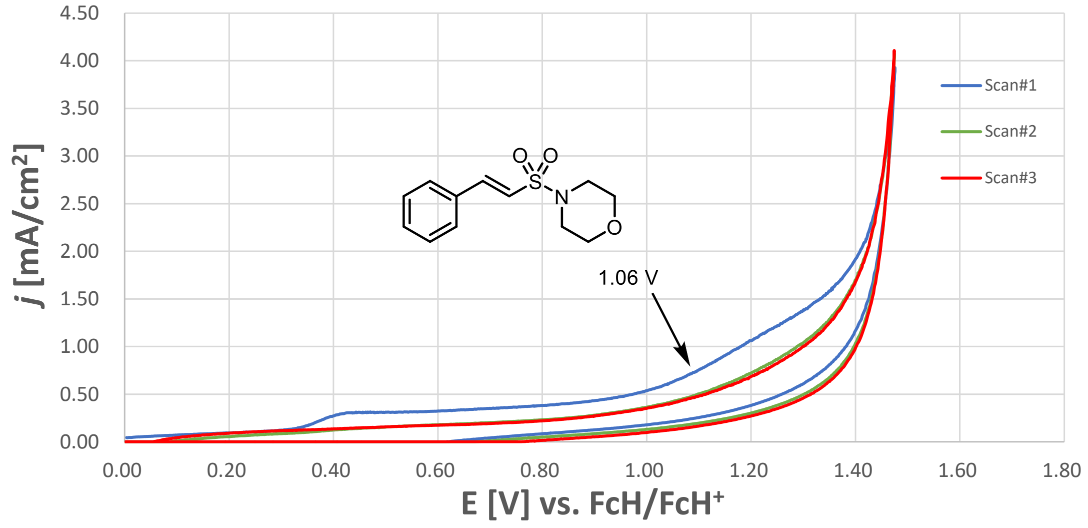

Figure S12: Cyclic voltammograms of (E)-4-(styrylsulfonyl)morpholine **7a**.

10 References

30 A. de A. Bartolomeu, F. A. Breitschaft, D. Schollmeyer, R. A. Pilli, S. R. Waldvogel, *Chem. Eur. J.* **2024**, *30*, e202400557.

35 S. P. Blum, T. Karakaya, D. Schollmeyer, A. Klapars, S. R. Waldvogel, *Angew. Chem. Int. Ed.* **2021**, *60*, 5056.

40 P.-C. Chien, F. A. Breitschaft, H. Kelm, S. R. Waldvogel, G. Manolikakes, *ChemSusChem* **2025**, *18*, e202500186.

55 S. Leong, S. Faudzi, F. Abas, M. Aluwi, K. Rullah, L. Wai, N. Lajis, *Molecules* **2014**, *19*, 16058.

56 S. Maki, A. Kojima, H. Tanba, JP2009184932 A, **2009**.

57 J. B. Ferguson, *J. Am. Chem. Soc.* **1917**, *39*, 364.

11 X-ray crystallographic data for selected compound

Table S4: Crystal data for **7a** (CCDC no. 2472612, the thermal ellipsoid drawn at 50% probability level).

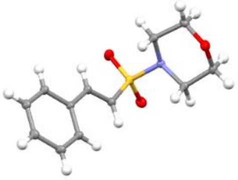

The purified compound **7a** was dissolved in a minimal amount of EtOAc in a glass vial. Hexanes were then added until the solution became turbid. The solution was allowed to slowly evaporate. After a few days, colorless crystals were obtained.

Empirical formula	C12 H15 N O3 S	
Formula weight	253.31	
Temperature	100(2) K	
Wavelength	0.71073 Å	
Crystal system	Monoclinic	
Space group	$P2_1/c$	
Unit cell dimensions	a = 13.5556(4) Å	α= 90°.
	b = 9.4267(3) Å	β= 99.3800(10)°.
	c = 9.4423(3) Å	γ = 90°.
Volume	1190.45(6) $Å^3$	
Z	4	
Density (calculated)	1.413 Mg/m^3	
Absorption coefficient	0.268 mm^{-1}	
F(000)	536	
Crystal size	0.226 x 0.176 x 0.125 mm^3	
Theta range for data collection	2.643 to 30.507°.	
Index ranges	-19<=h<=16, -13<=k<=13, -13<=l<=13	
Reflections collected	37726	
Independent reflections	3636 [R(int) = 0.0775]	
Completeness to theta = 25.242°	99.9 %	
Absorption correction	Semi-empirical from equivalents	
Max. and min. transmission	1.000 and 0.919	
Refinement method	Full-matrix least-squares on F^2	
Data / restraints / parameters	3636 / 0 / 157	
Goodness-of-fit on F^2	1.048	
Final R indices [I>2sigma(I)]	R1 = 0.0275, wR2 = 0.0683	
R indices (all data)	R1 = 0.0350, wR2 = 0.0711	
Extinction coefficient	n/a	
Largest diff. peak and hole	0.278 and -0.288 e.$Å^{-3}$	

12 ^{1}H, ^{13}C NMR, and ^{19}F NMR spectra for compounds 7, 8, and 9

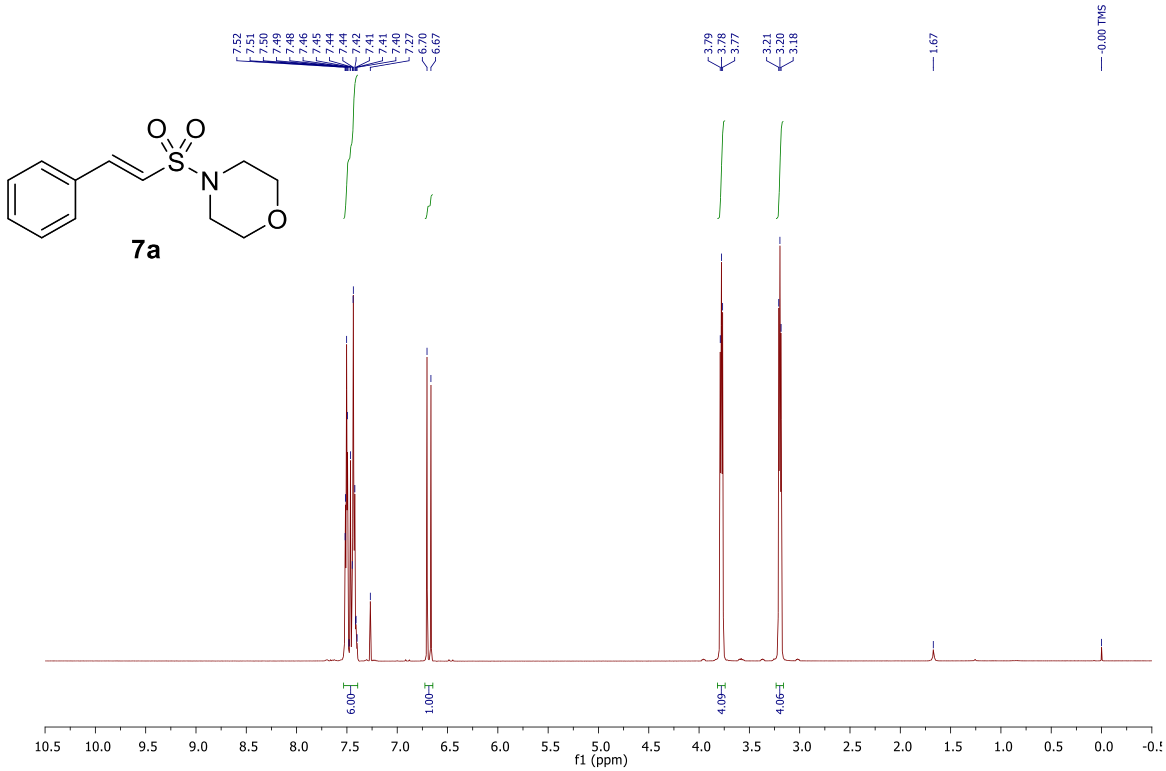

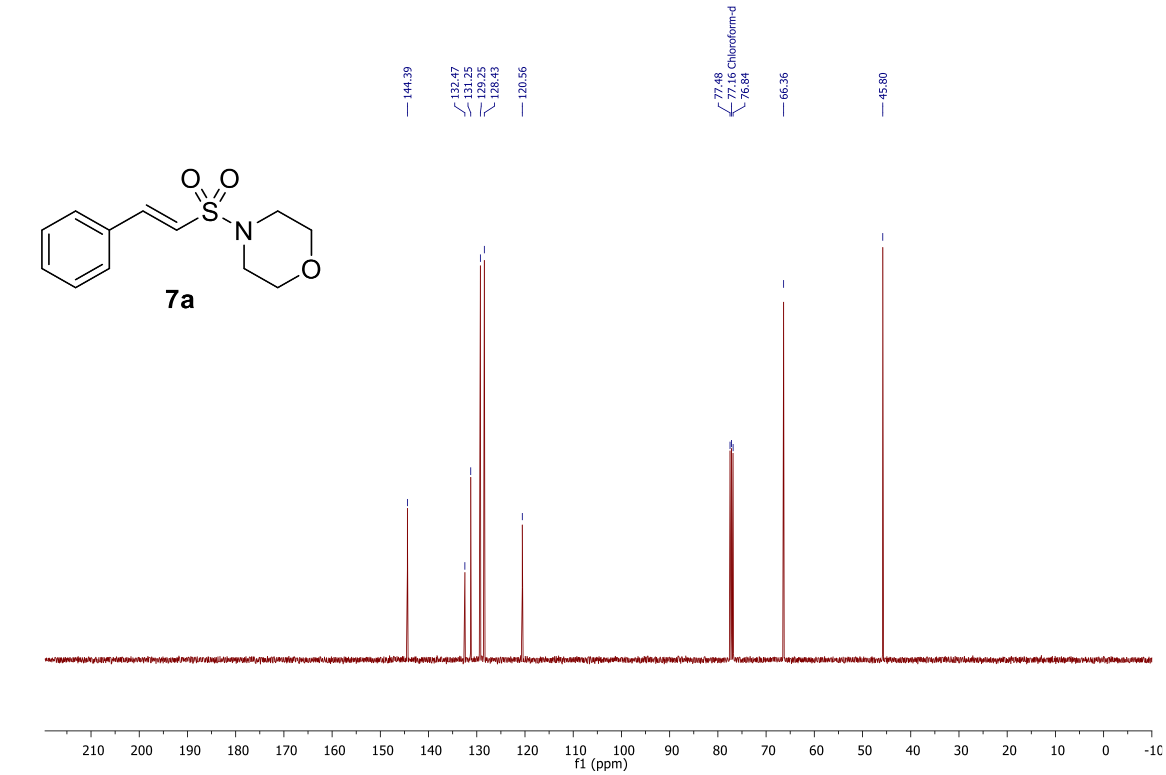

Figure S13: ^{1}H ($CDCl_3$, 400 MHz) and $^{13}C\{^{1}H\}$ ($CDCl_3$, 101 MHz) NMR Spectrum of **7a**.

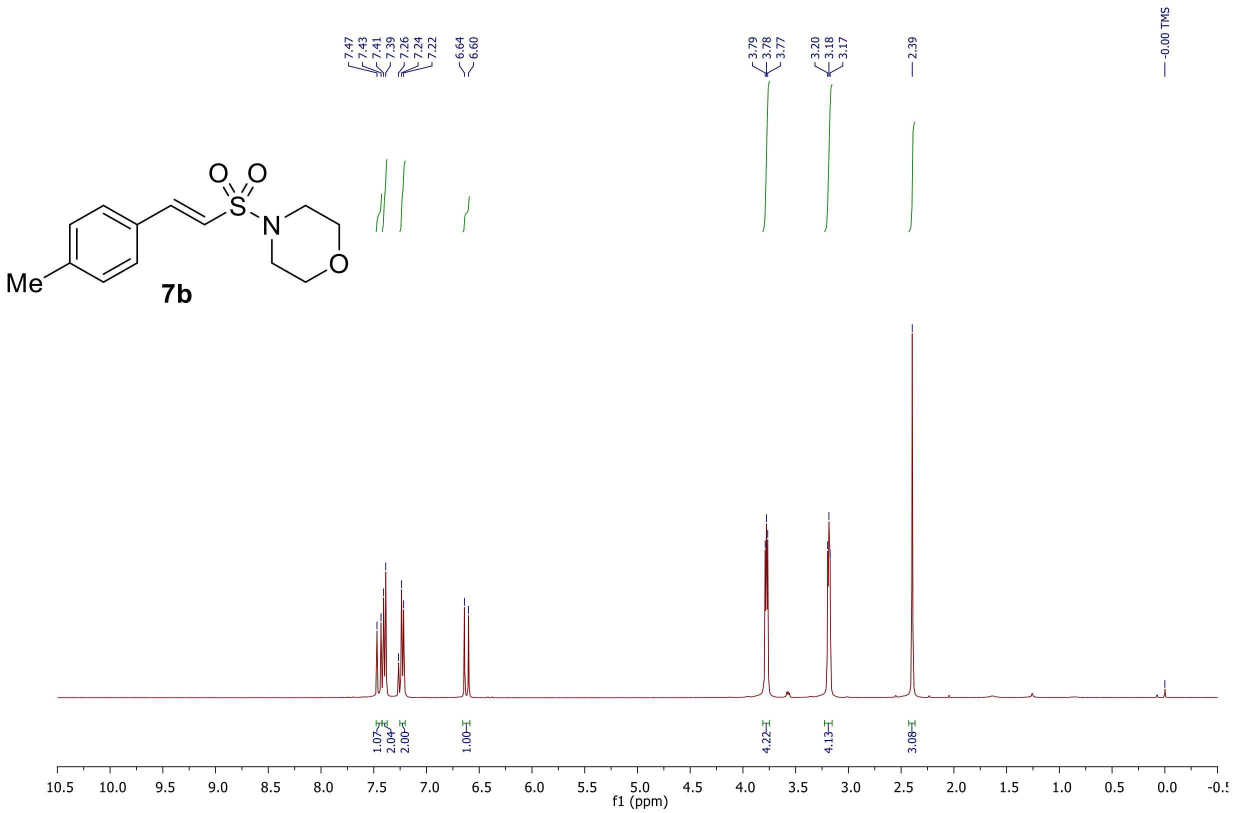

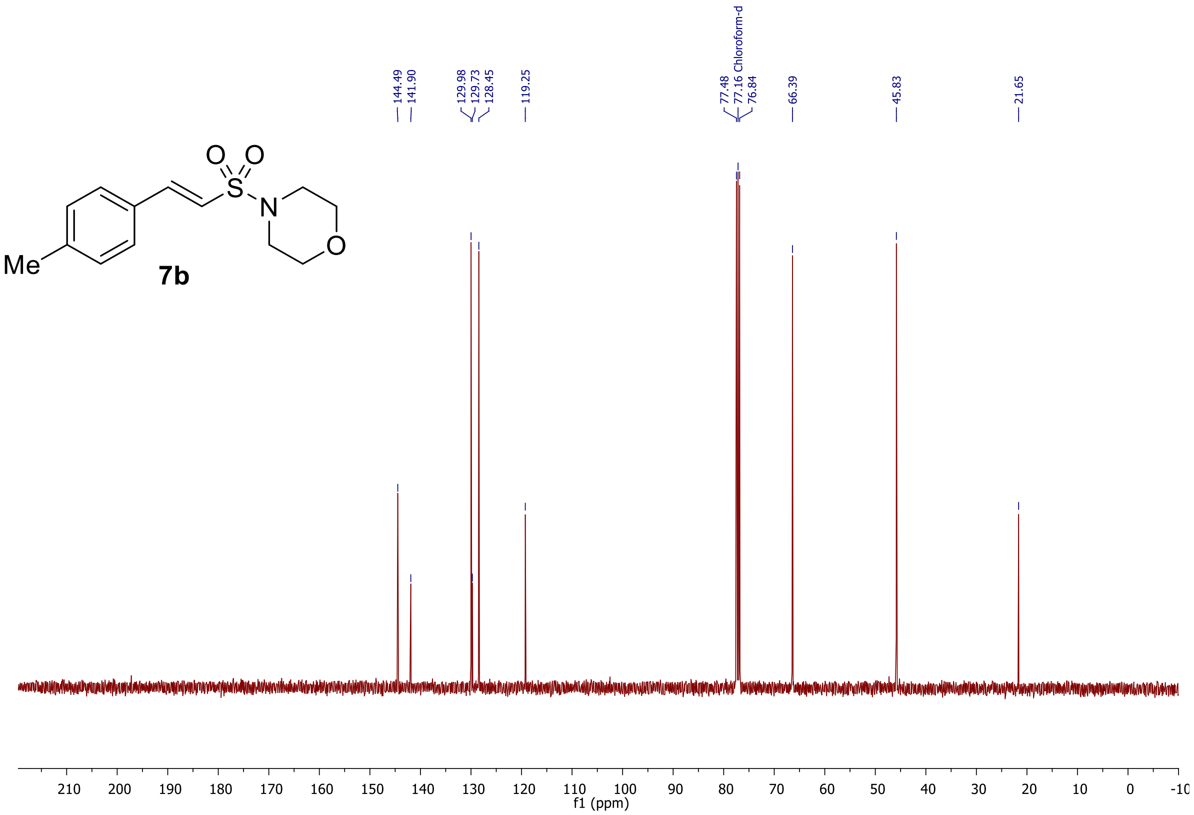

Figure S14: ^{1}H ($CDCl_3$, 400 MHz) and $^{13}C\{^{1}H\}$ ($CDCl_3$, 101 MHz) NMR Spectrum of **7b**.

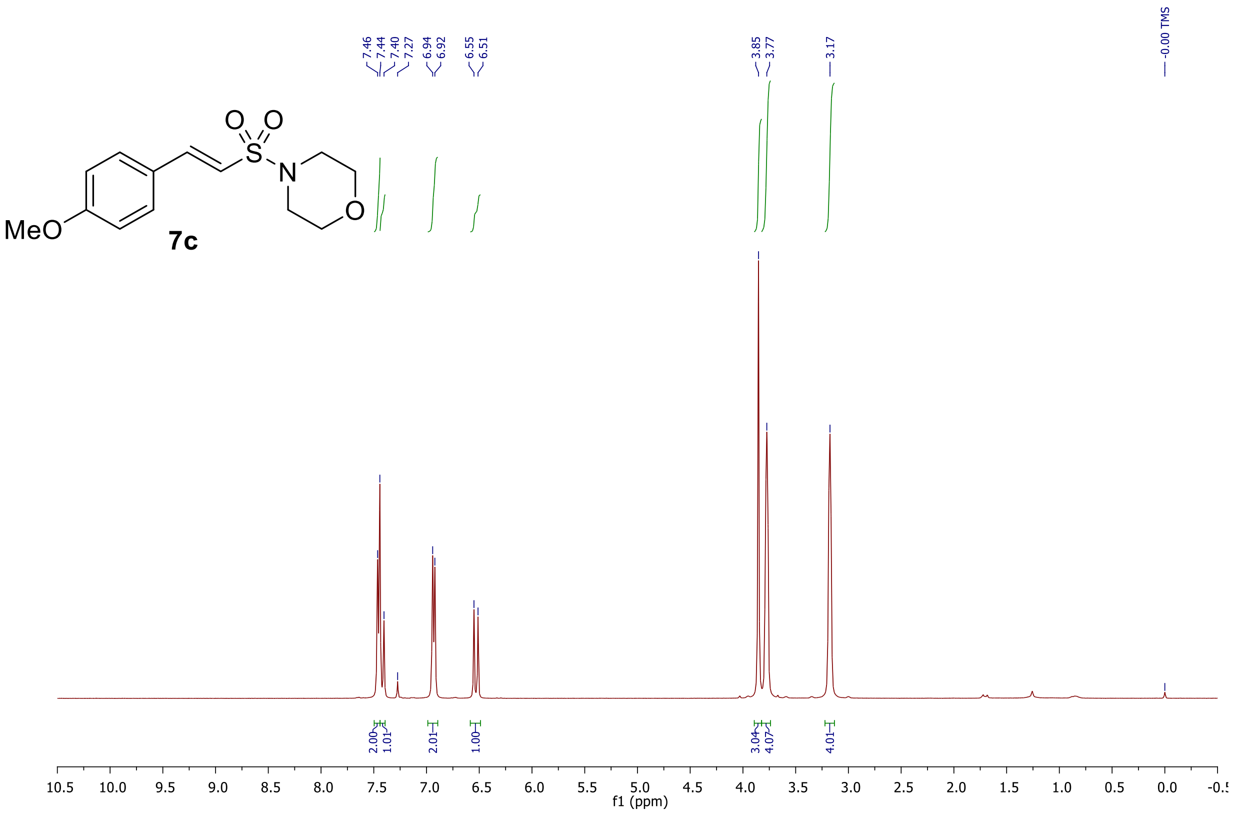

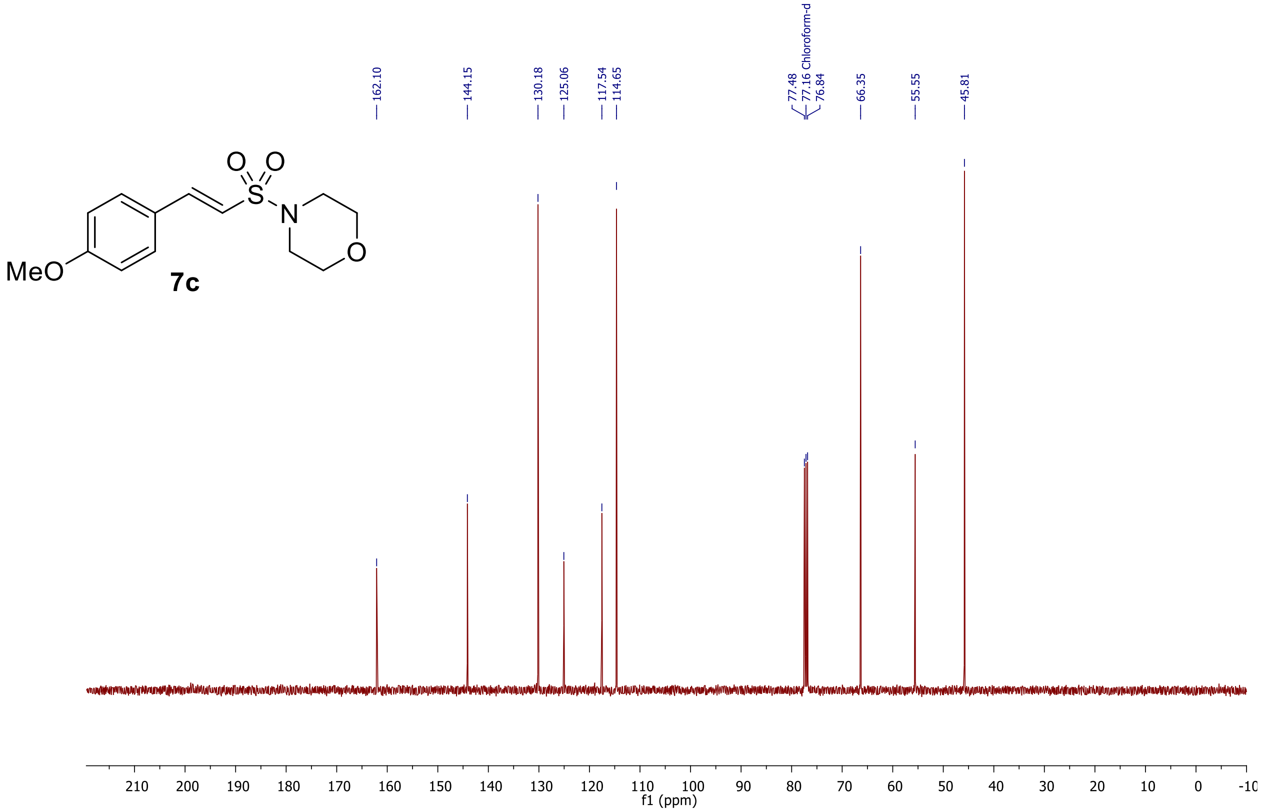

Figure S15: ^{1}H ($CDCl_3$, 400 MHz) and ^{13}C{^{1}H} ($CDCl_3$, 101 MHz) NMR Spectrum of **7c**.

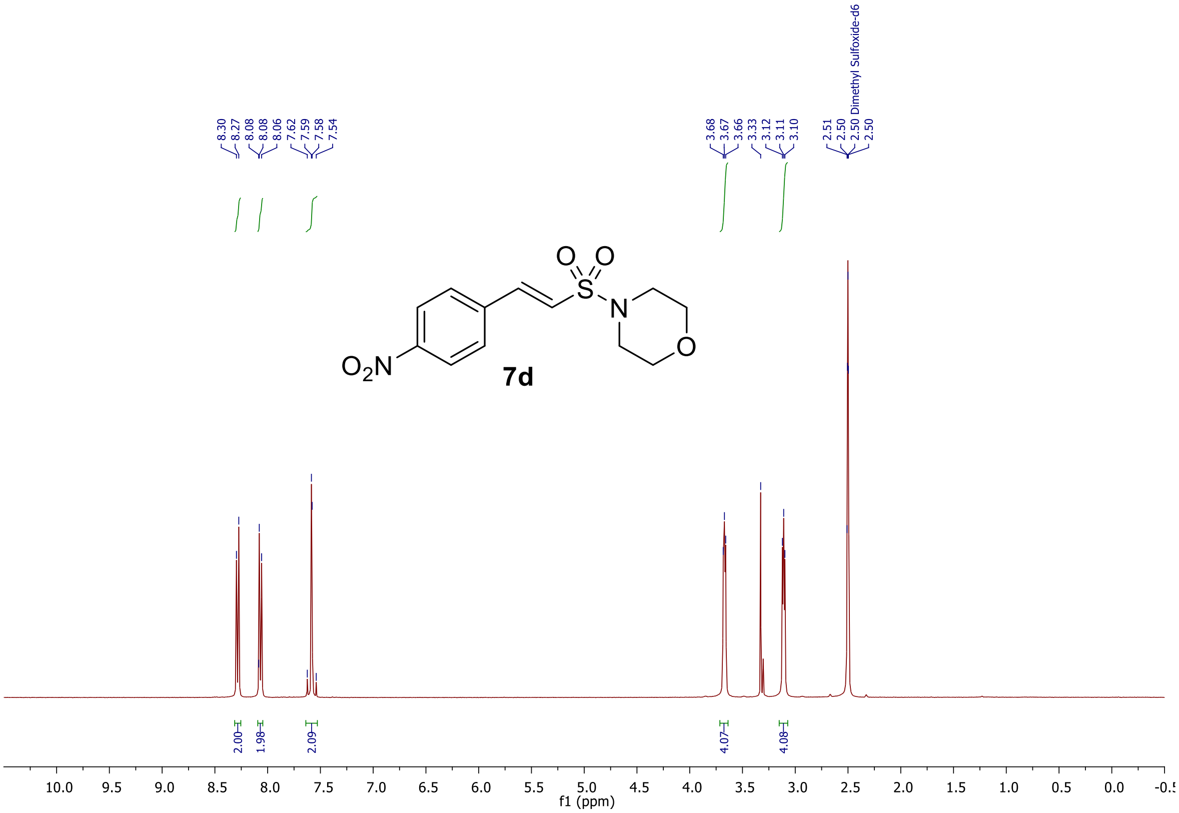

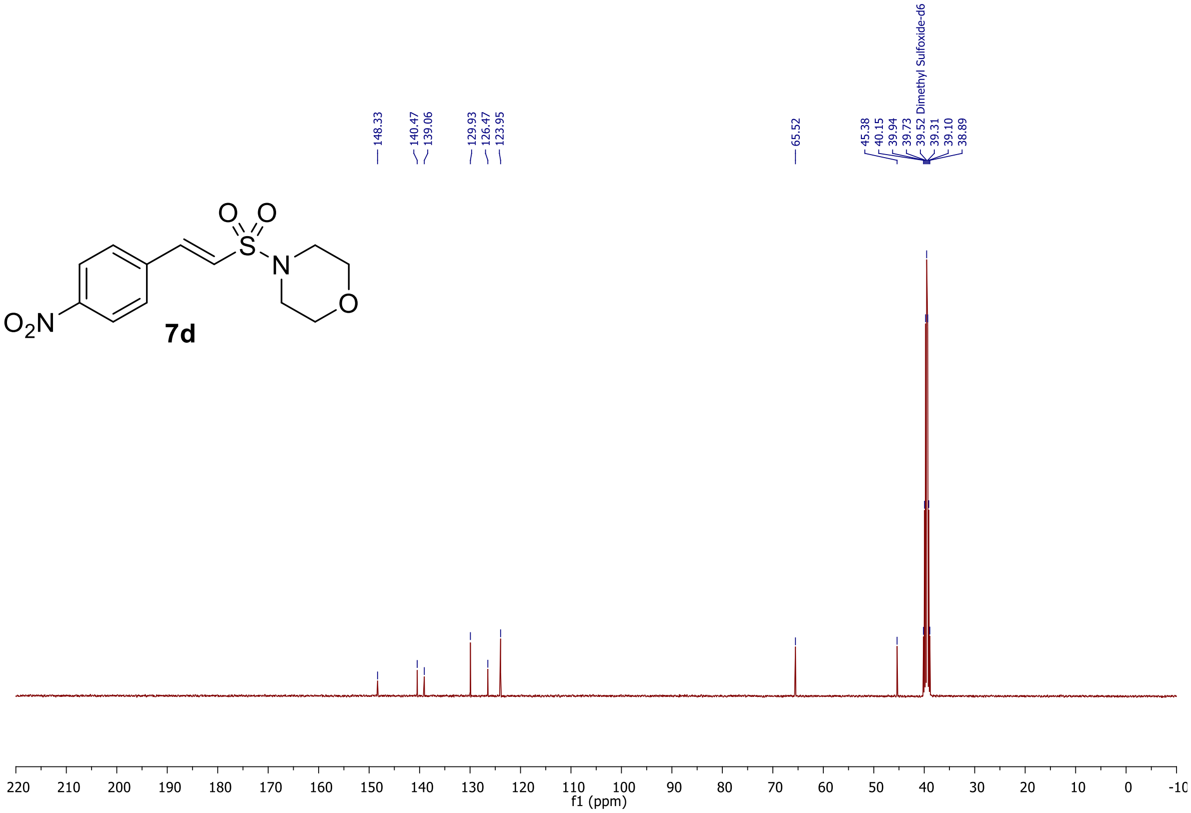

Figure S16: ^{1}H (DMSO-d_6, 400 MHz) and ^{13}C{^{1}H} (DMSO-d_6, 101 MHz) NMR Spectrum of **7d**.

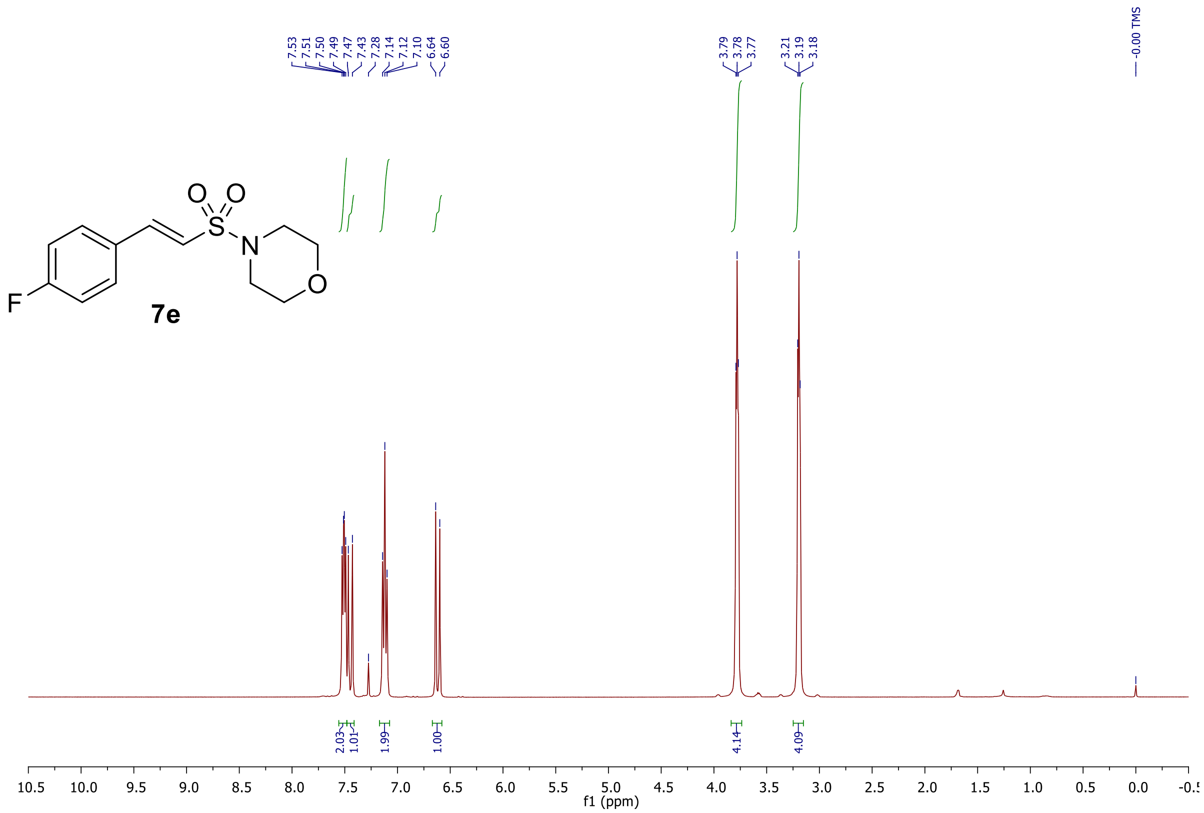
7.53
7.51
7.50
7.49
7.47
7.43
7.28
7.14
7.12
7.10
6.64
6.60
3.79
3.78
3.77
3.21
3.19
3.18
0.00 TMS
2.03
1.01
1.99
1.00
4.14
4.09
f1 (ppm)

7e

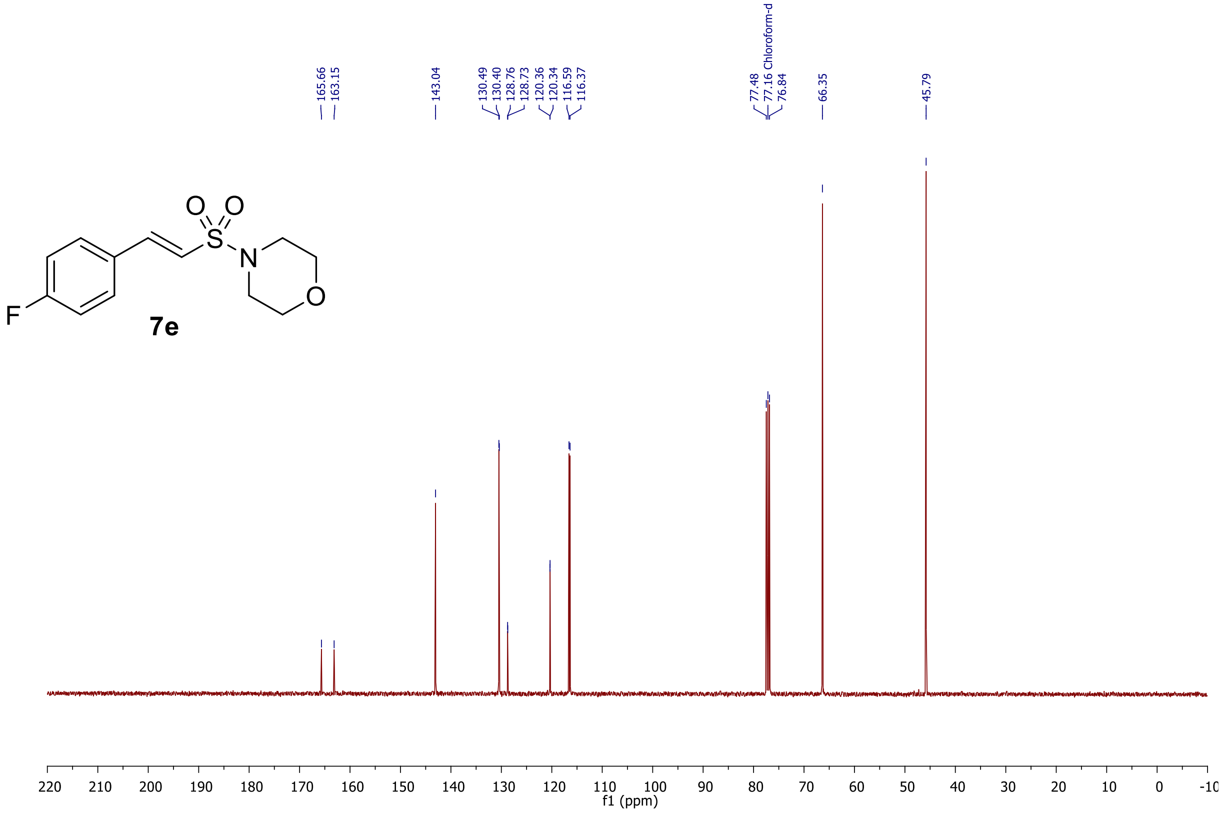
165.66
163.15
143.04
130.49
130.40
128.76
128.73
120.36
120.34
116.59
116.37
77.48
77.16 Chloroform-d
76.84
66.35
45.79
f1 (ppm)

7e

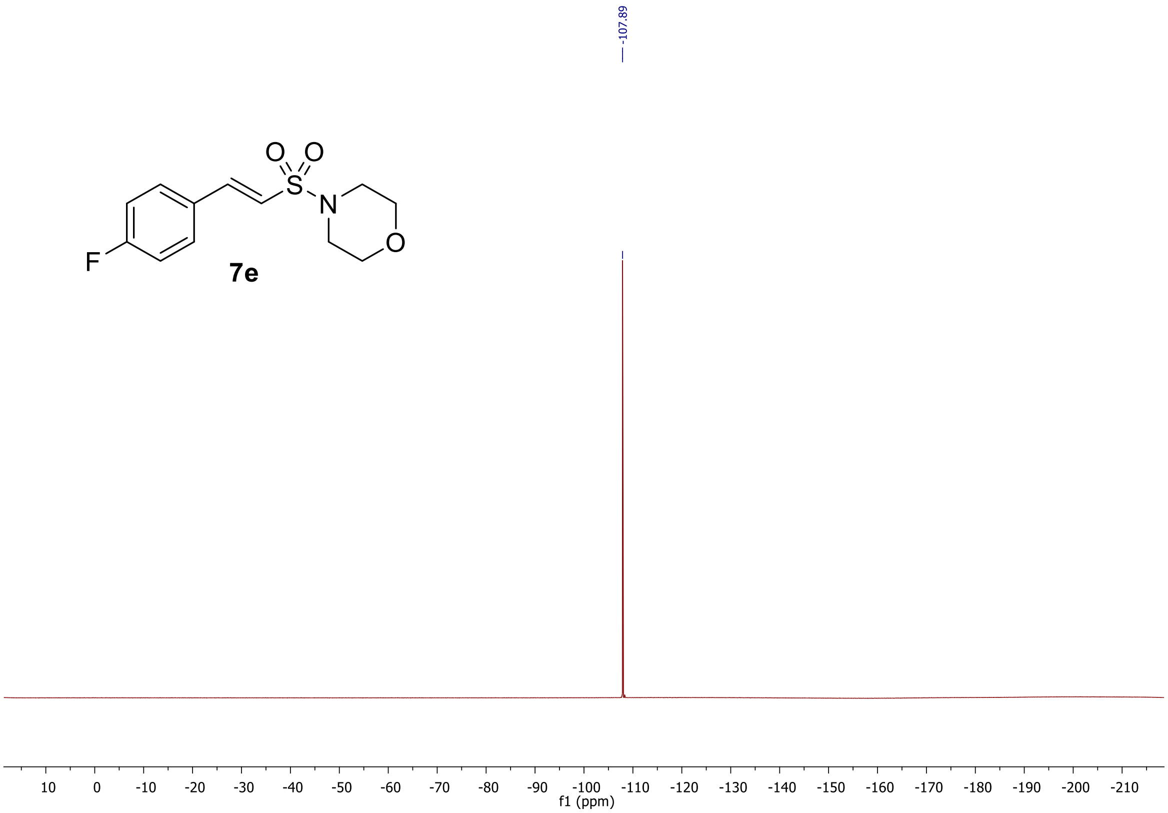

Figure S17: ^{1}H ($CDCl_3$, 400 MHz), $^{13}C\{^1H\}$ ($CDCl_3$, 101 MHz), and $^{19}F\{^1H\}$ ($CDCl_3$, 376 MHz) NMR Spectrum of **7e**.

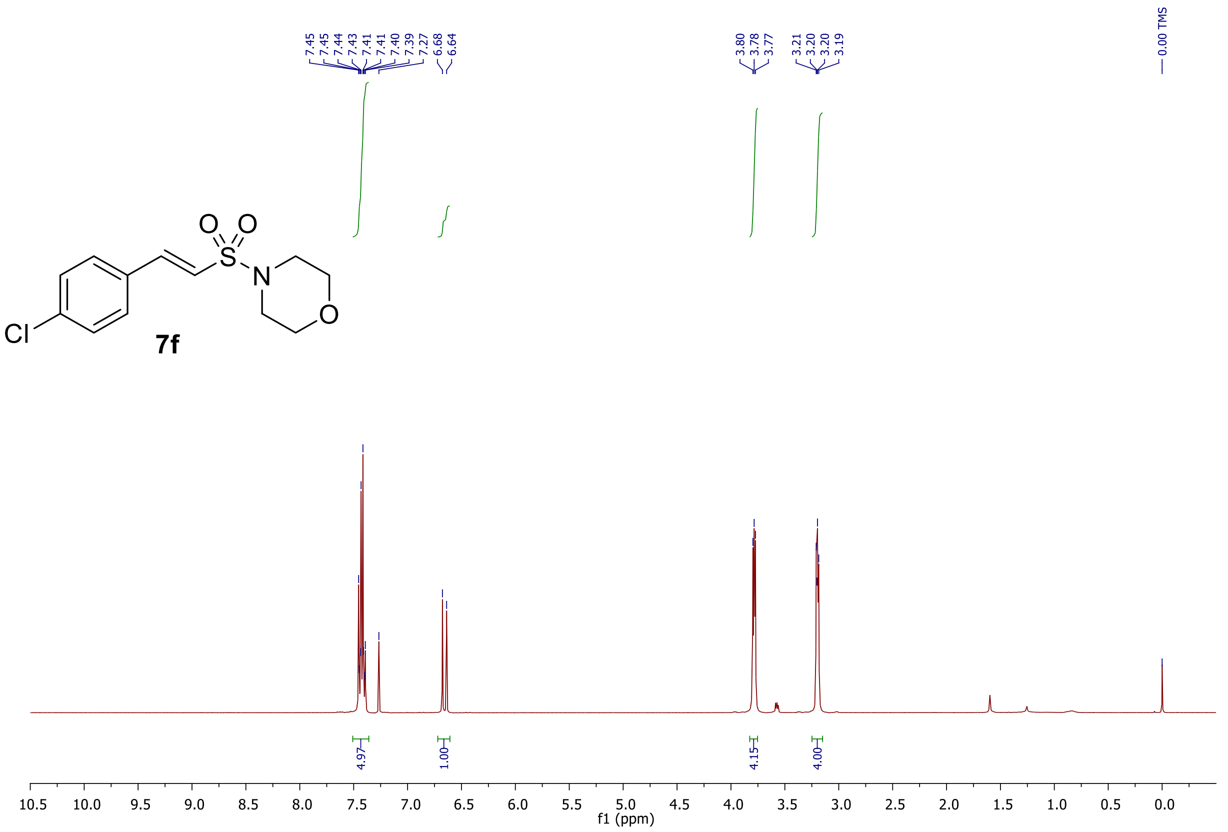

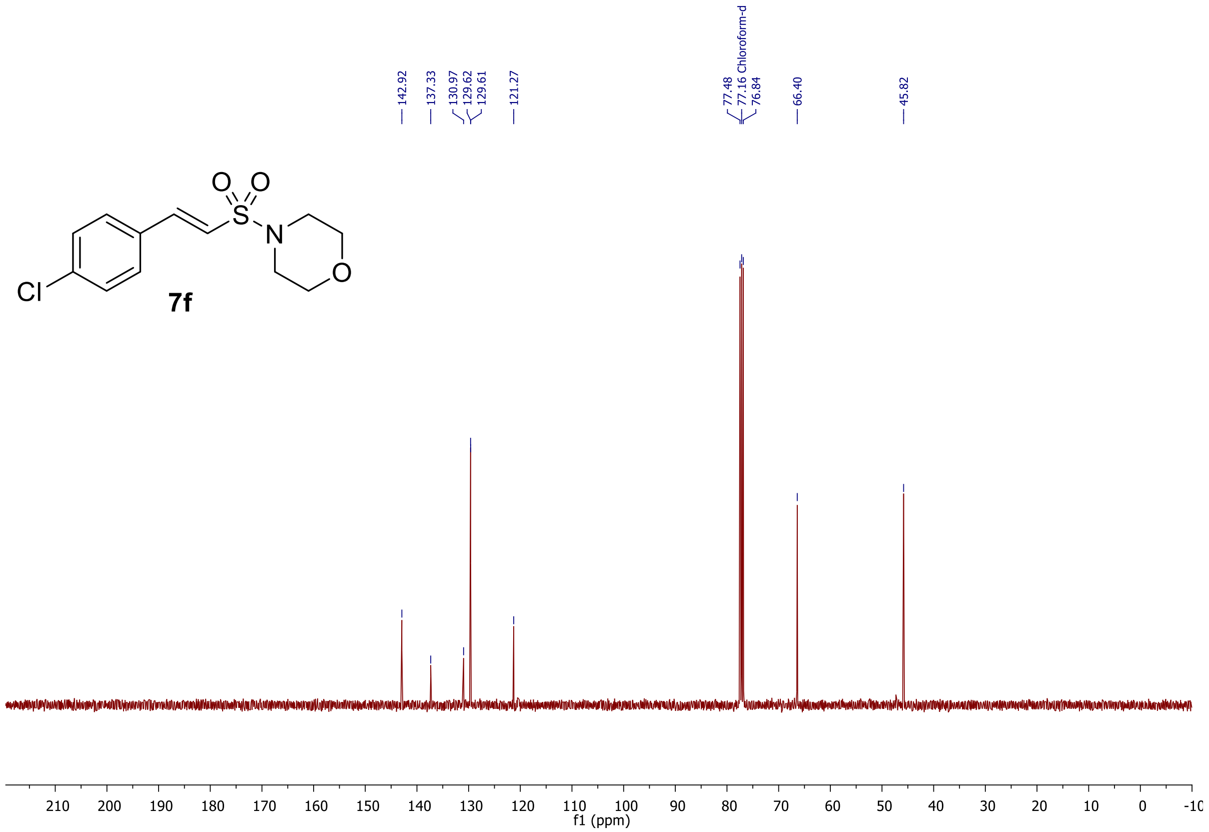

Figure S18: ^{1}H ($CDCl_3$, 400 MHz) and ^{13}C{^{1}H} ($CDCl_3$, 101 MHz) NMR Spectrum of **7f**.

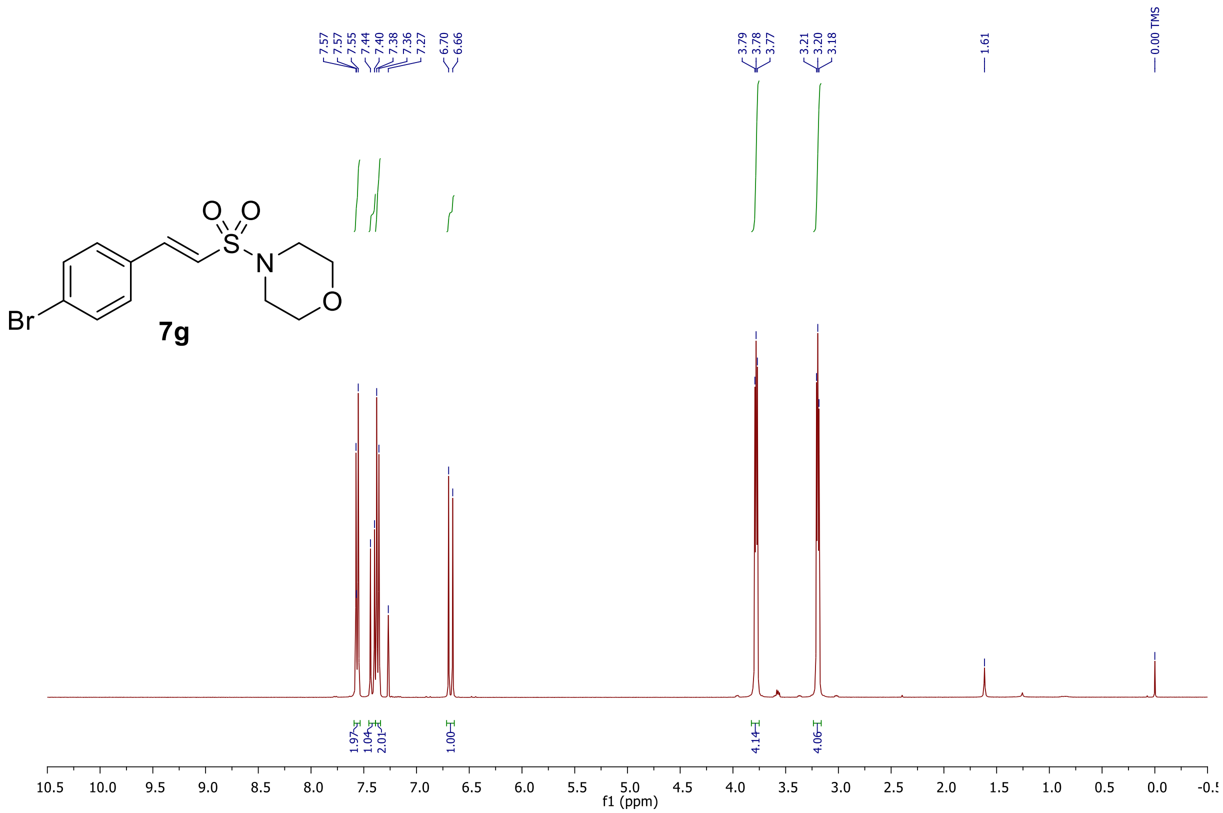

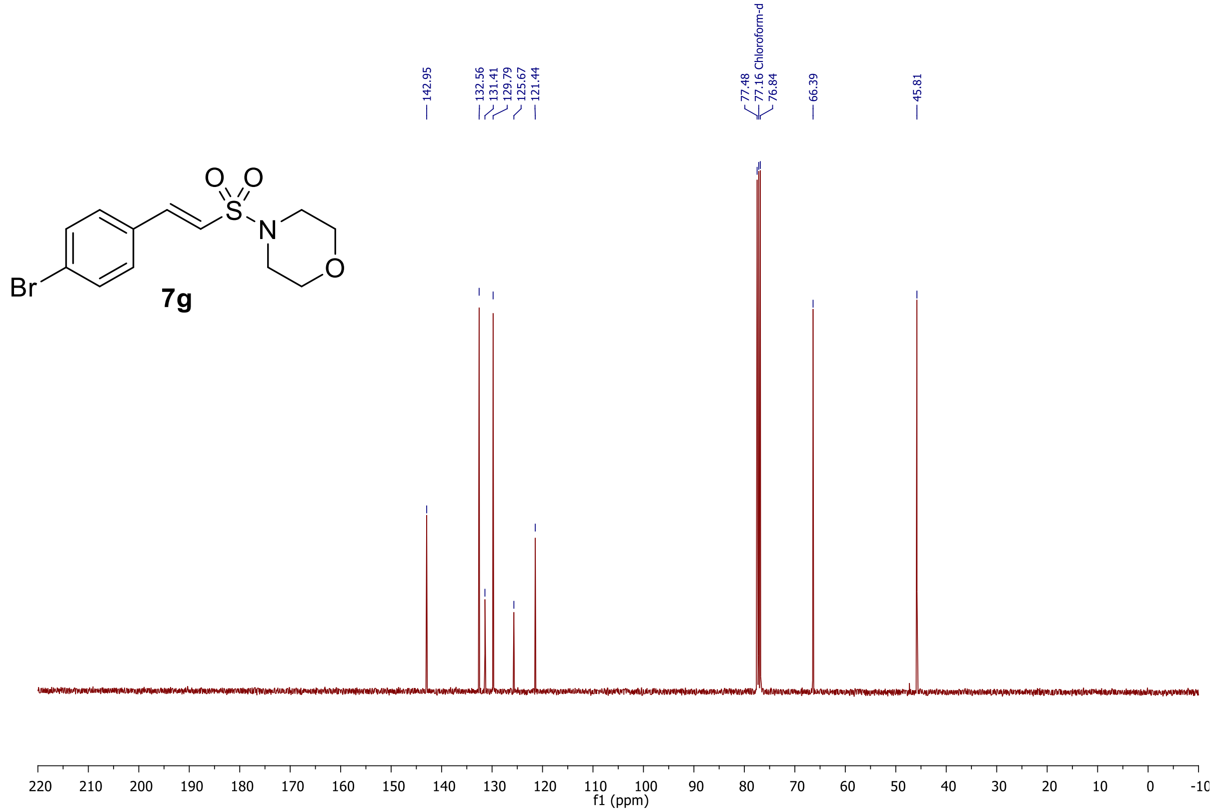

Figure S19: ^{1}H ($CDCl_3$, 400 MHz) and $^{13}C\{^{1}H\}$ ($CDCl_3$, 101 MHz) NMR Spectrum of **7g**.

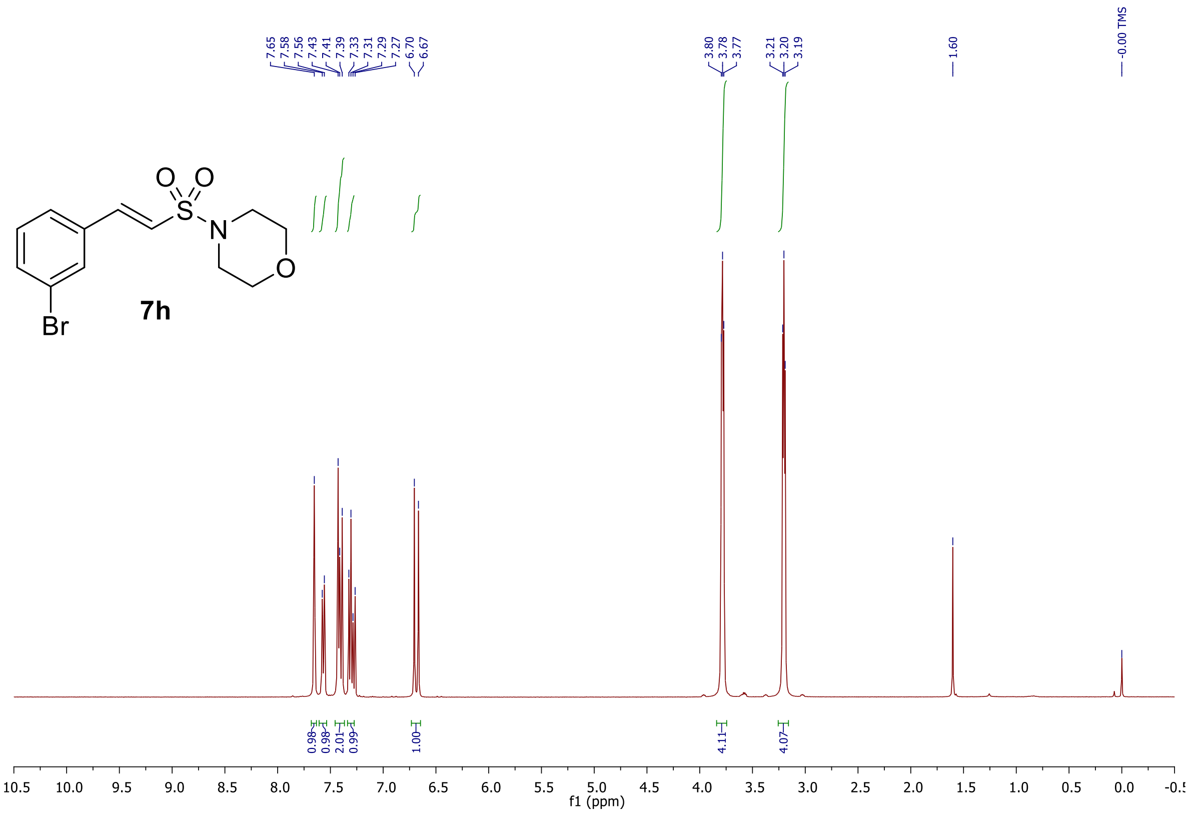

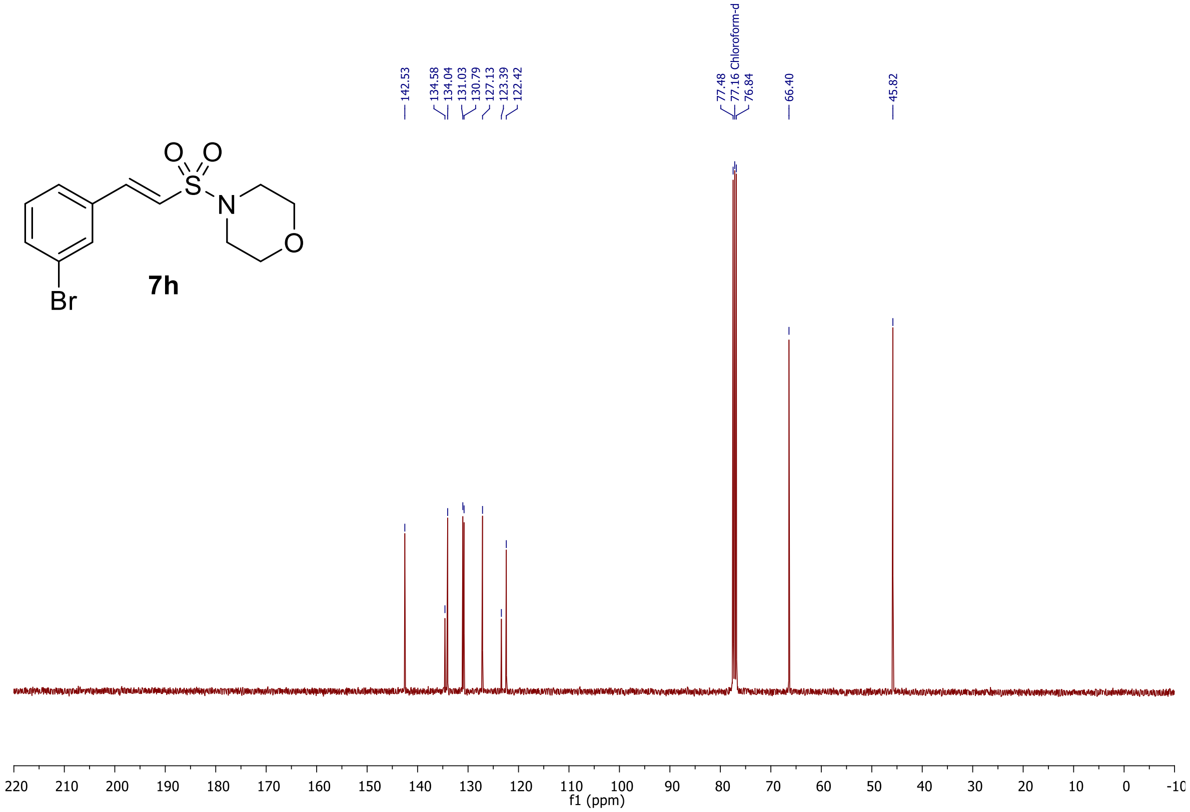

Figure S20: ^{1}H ($CDCl_3$, 400 MHz) and $^{13}C\{^{1}H\}$ ($CDCl_3$, 101 MHz) NMR Spectrum of **7h**.

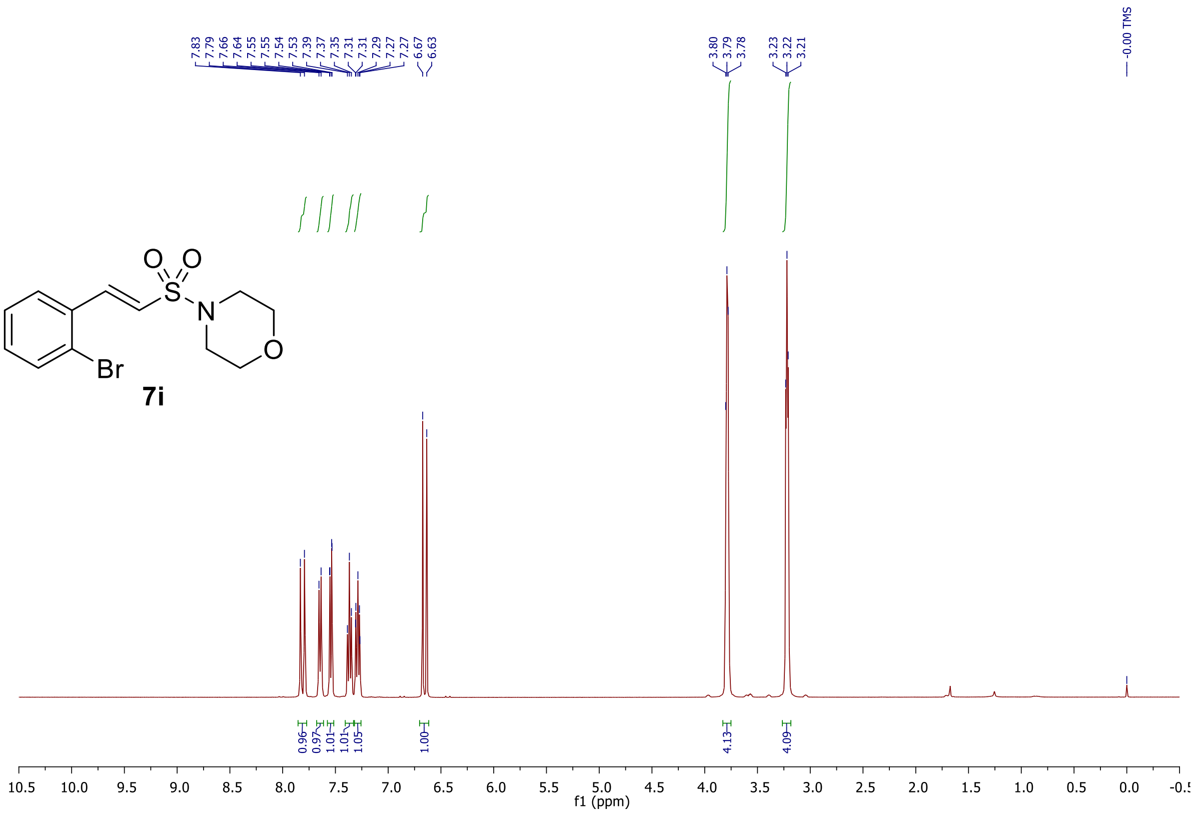

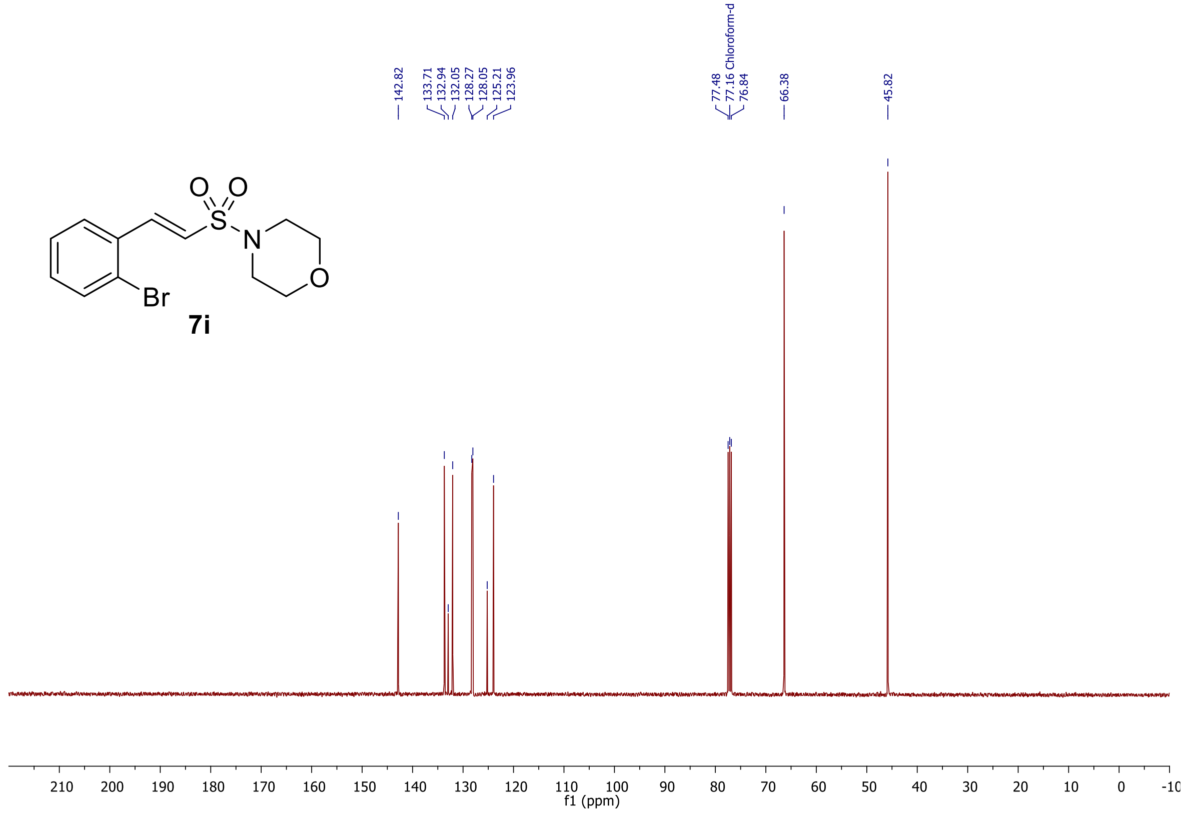

Figure S21: ^{1}H ($CDCl_3$, 400 MHz) and $^{13}C\{^{1}H\}$ ($CDCl_3$, 101 MHz) NMR Spectrum of **7i**.

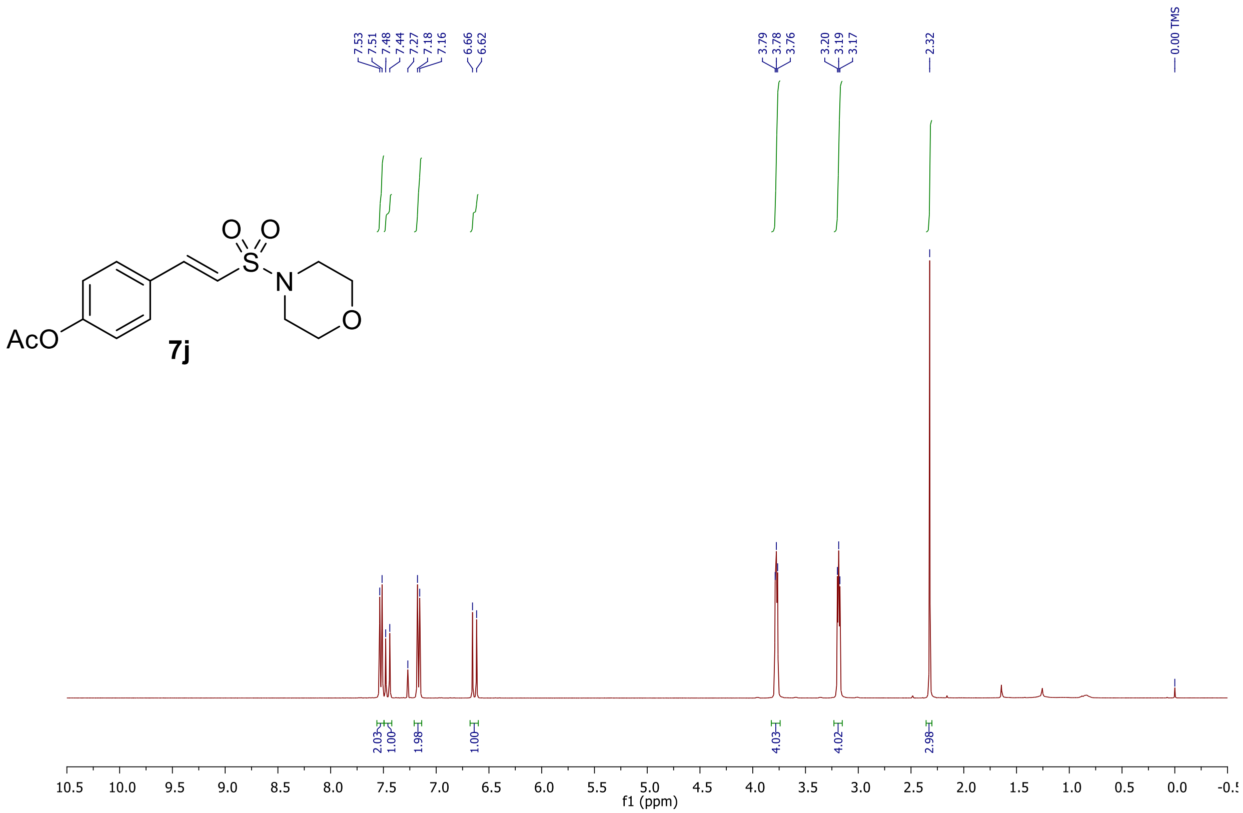

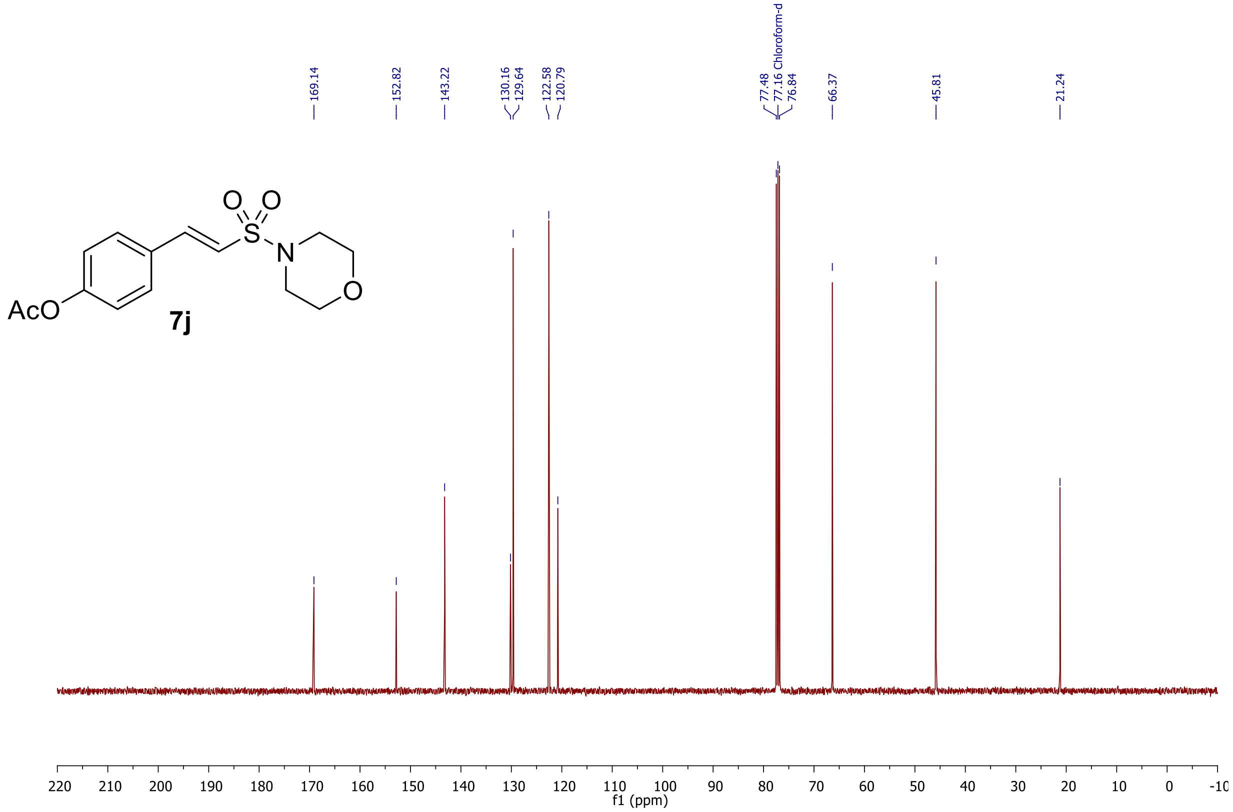

Figure S22: ^{1}H ($CDCl_3$, 400 MHz) and ^{13}C{^{1}H} ($CDCl_3$, 101 MHz) NMR Spectrum of **7j**.

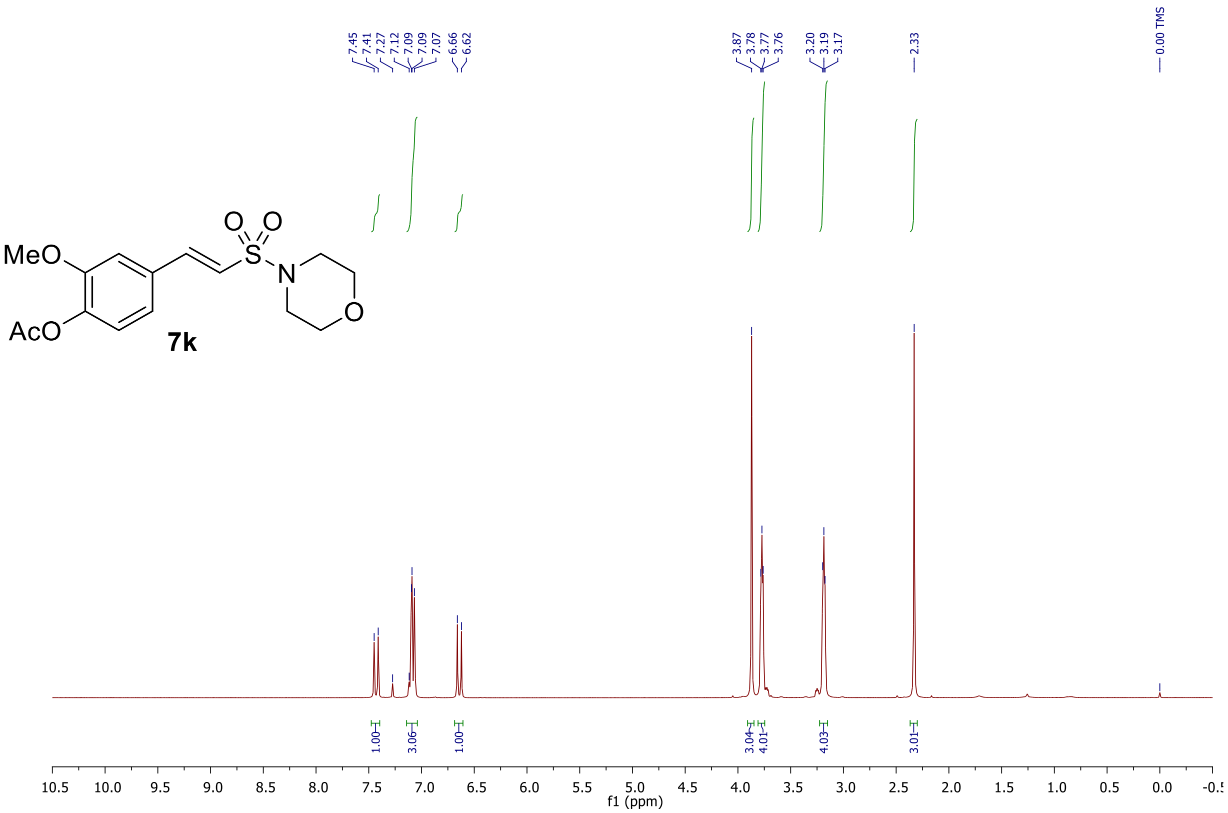

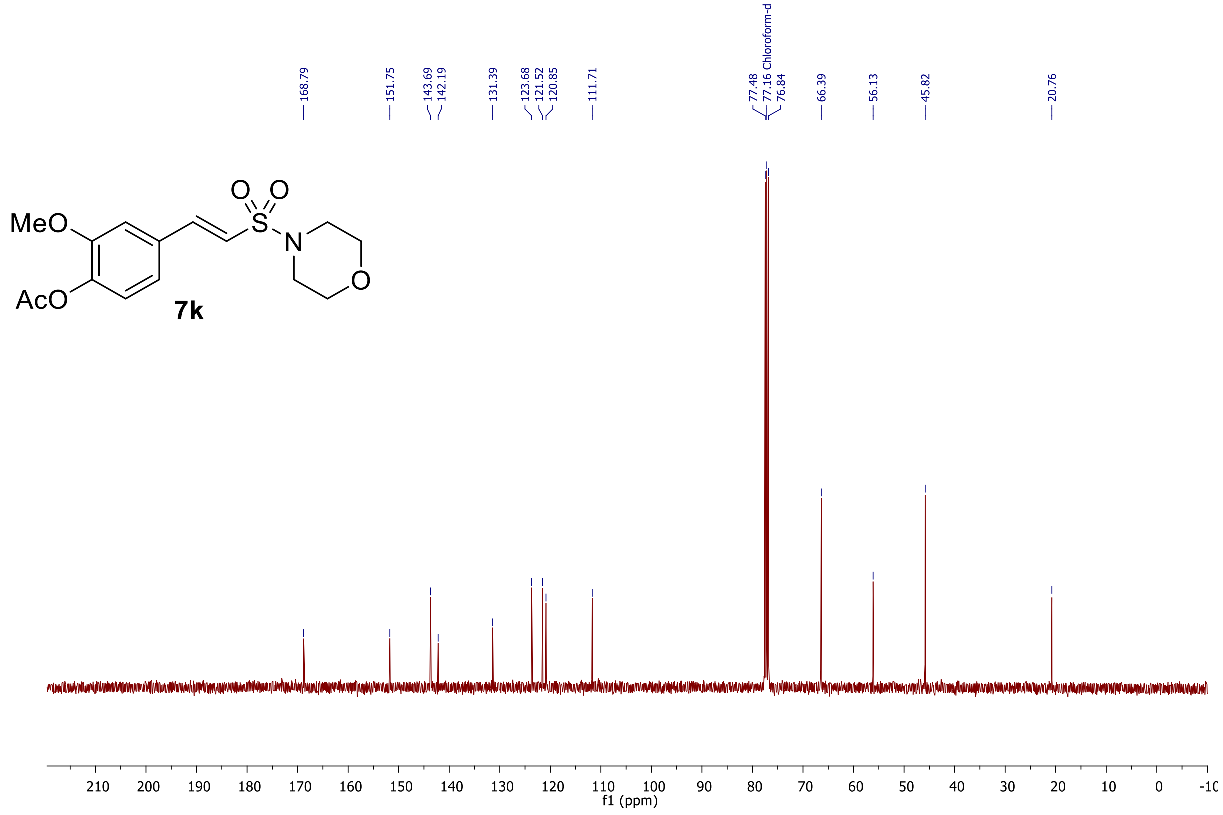

Figure S23: ^{1}H ($CDCl_3$, 400 MHz) and $^{13}C\{^{1}H\}$ ($CDCl_3$, 101 MHz) NMR Spectrum of **7k**.

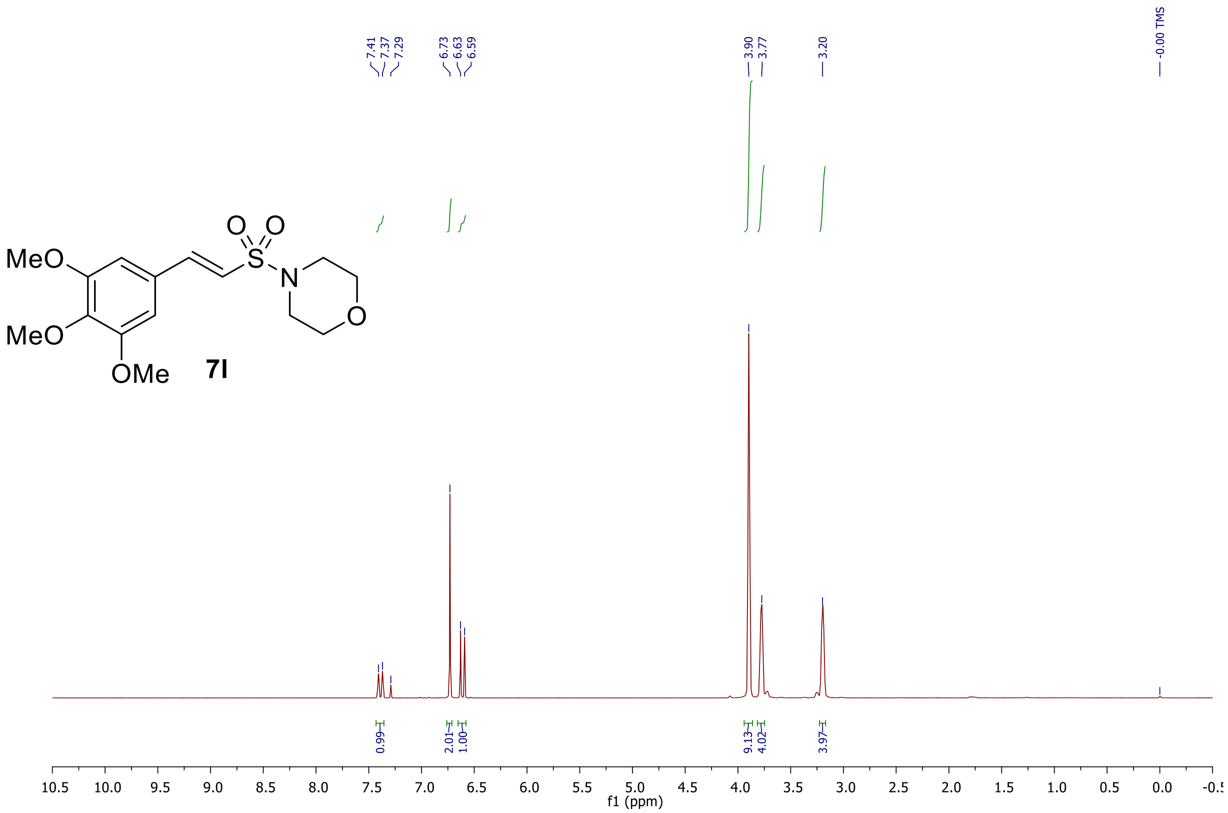

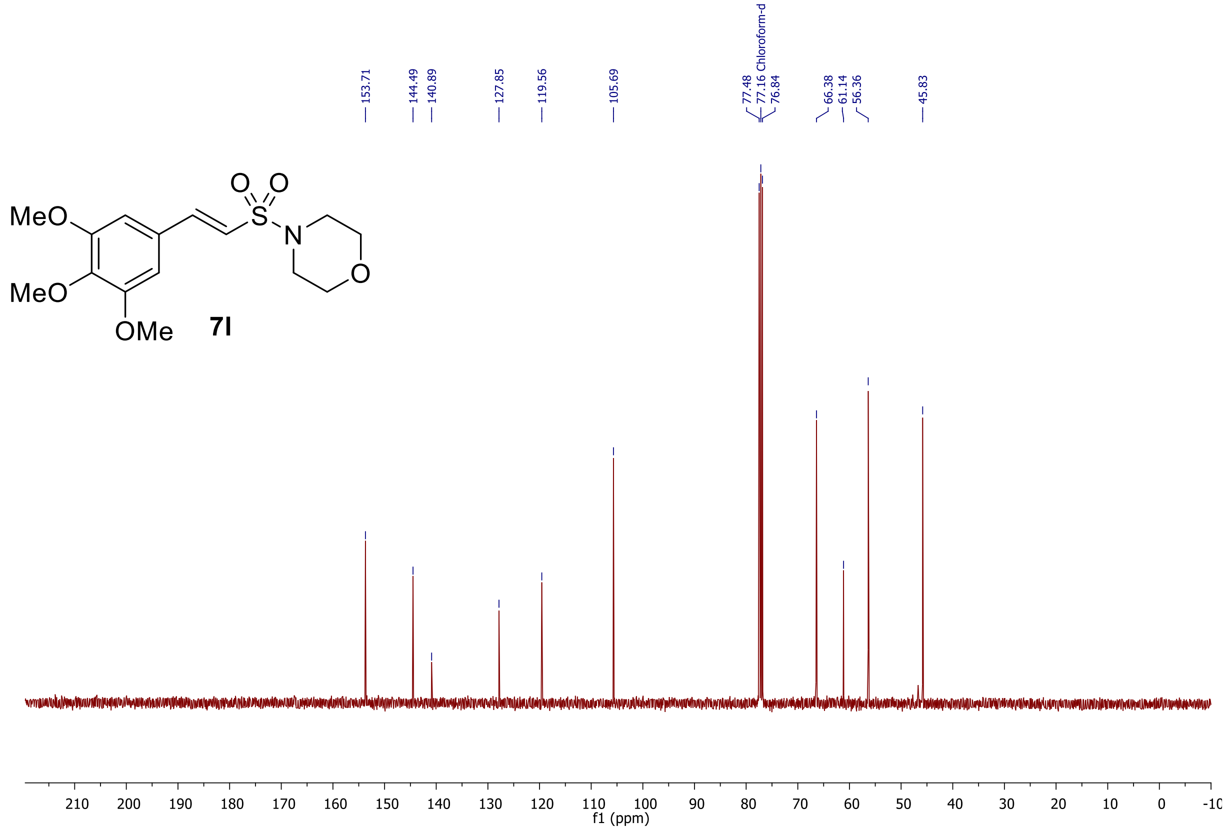

Figure S24: ^1H ($CDCl_3$, 400 MHz) and $^{13}C\{^1H\}$ ($CDCl_3$, 101 MHz) NMR Spectrum of **7l**.

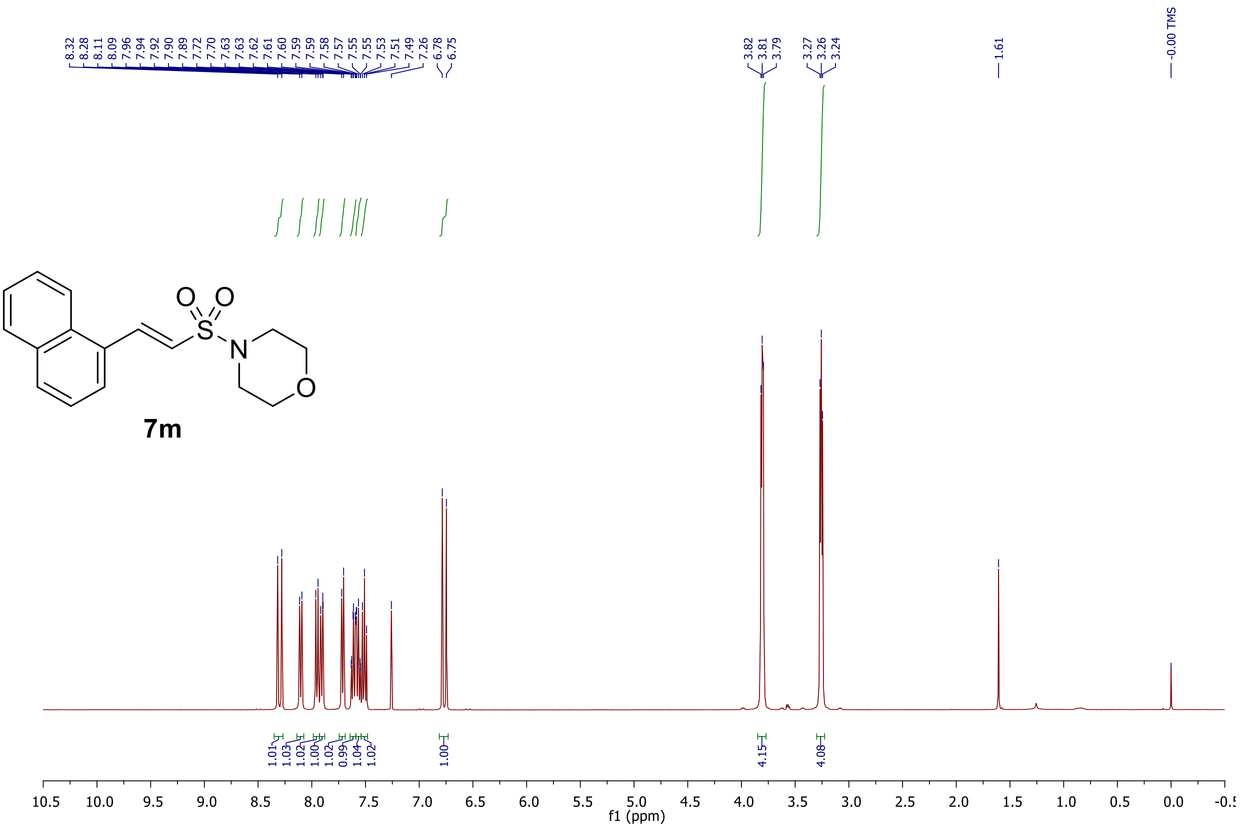

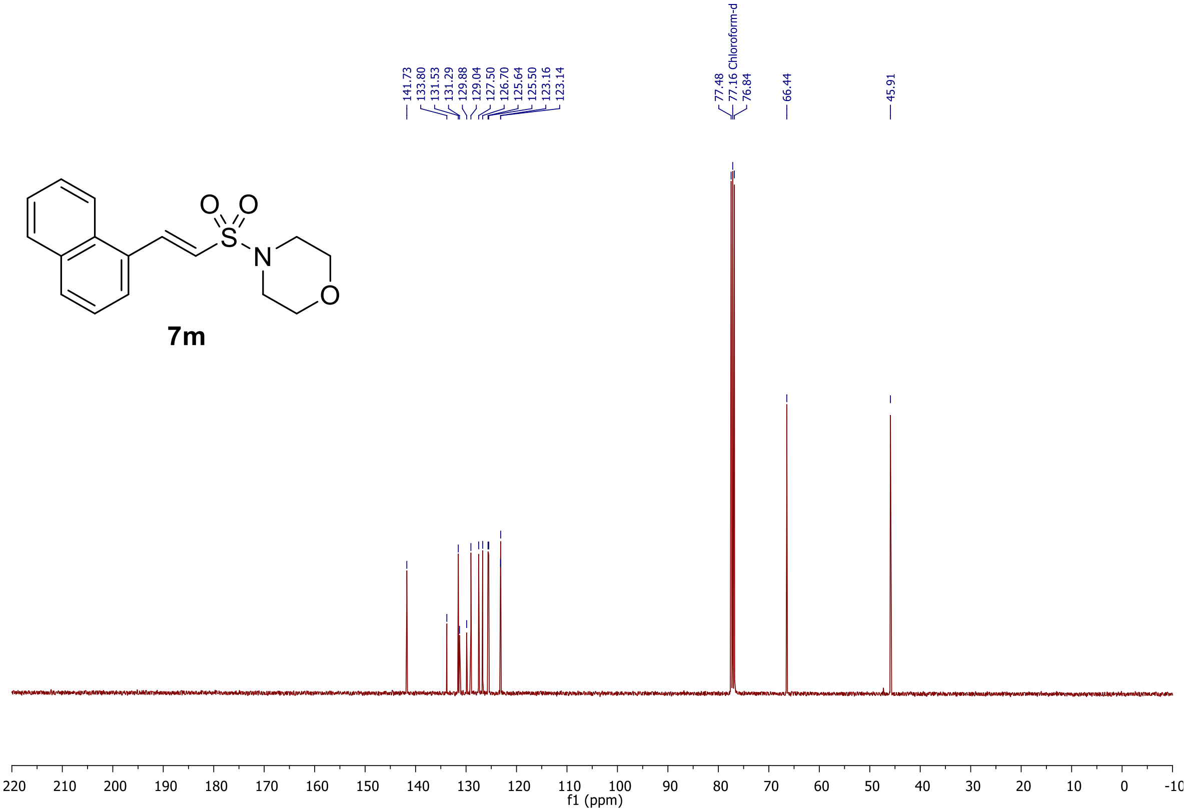

Figure S25: ^{1}H ($CDCl_3$, 400 MHz) and $^{13}C\{^{1}H\}$ ($CDCl_3$, 101 MHz) NMR Spectrum of **7m**.

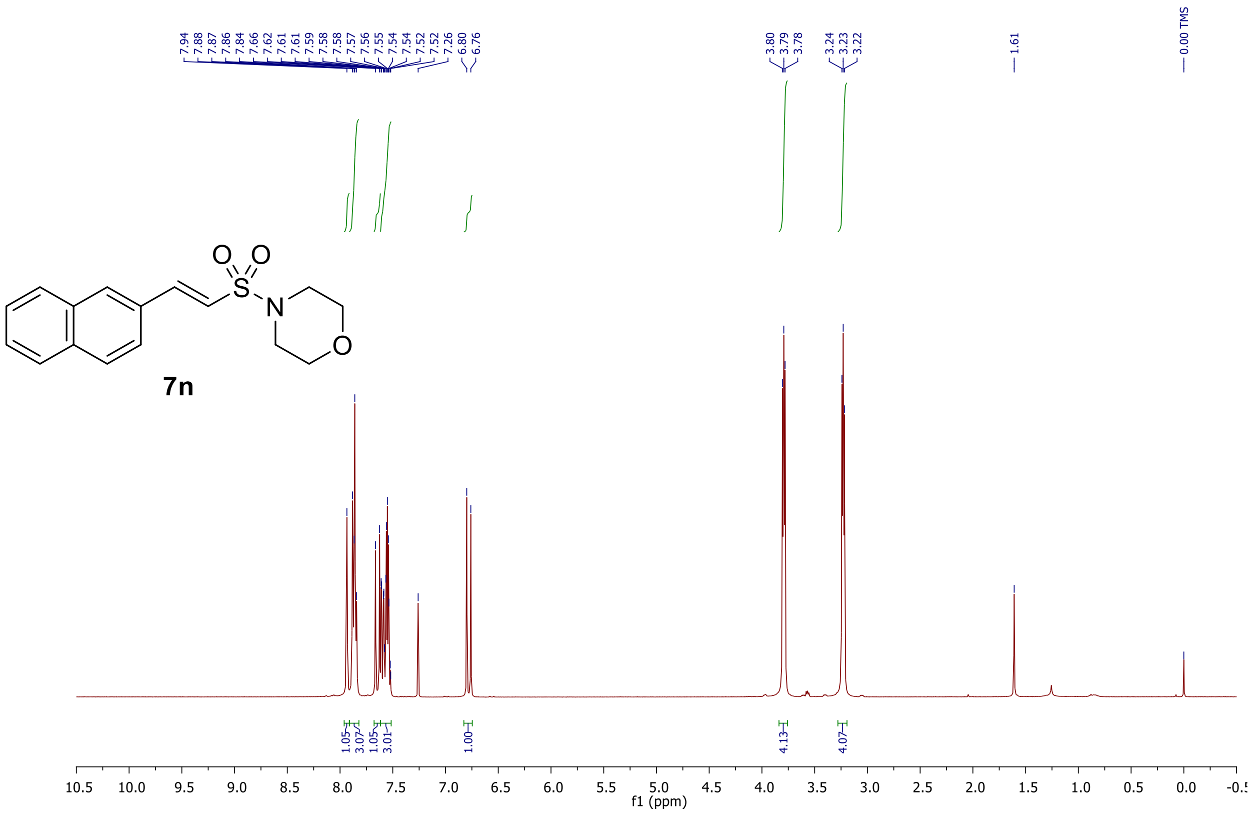

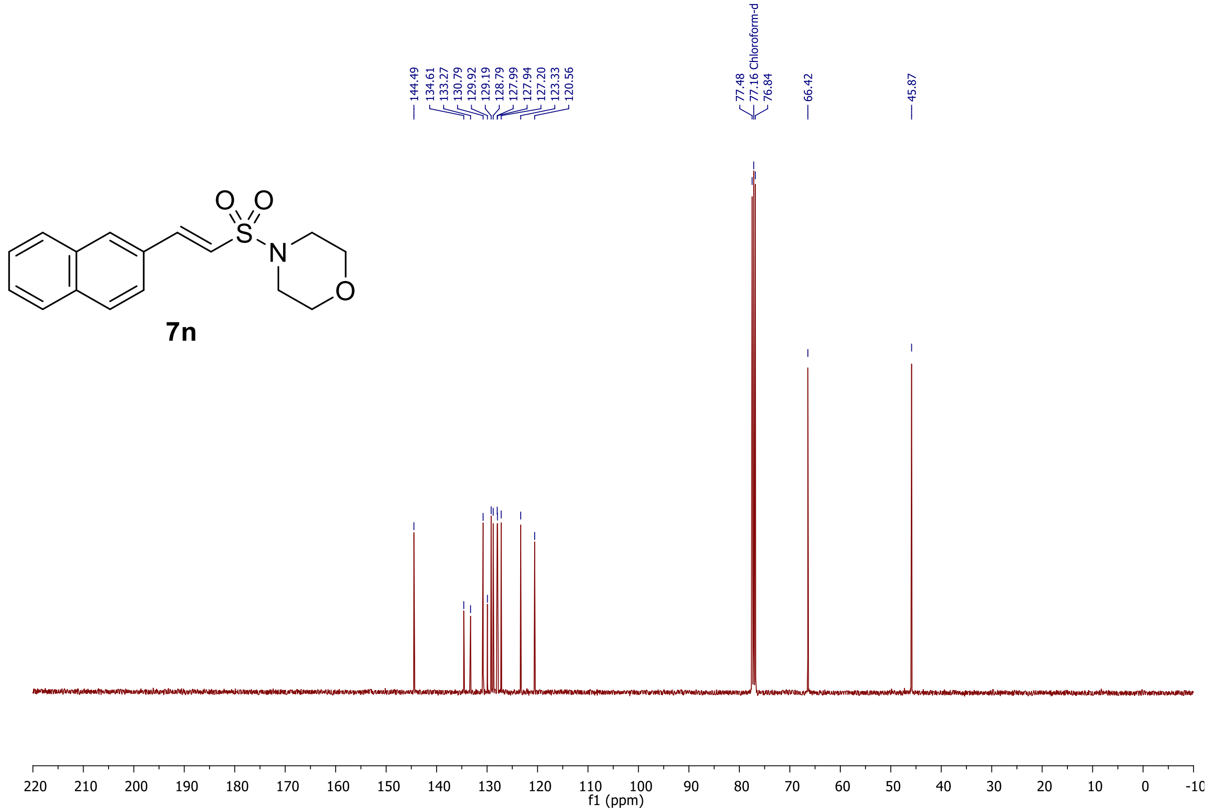

Figure S26: ^{1}H ($CDCl_3$, 400 MHz) and $^{13}C\{^{1}H\}$ ($CDCl_3$, 101 MHz) NMR Spectrum of **7n**.

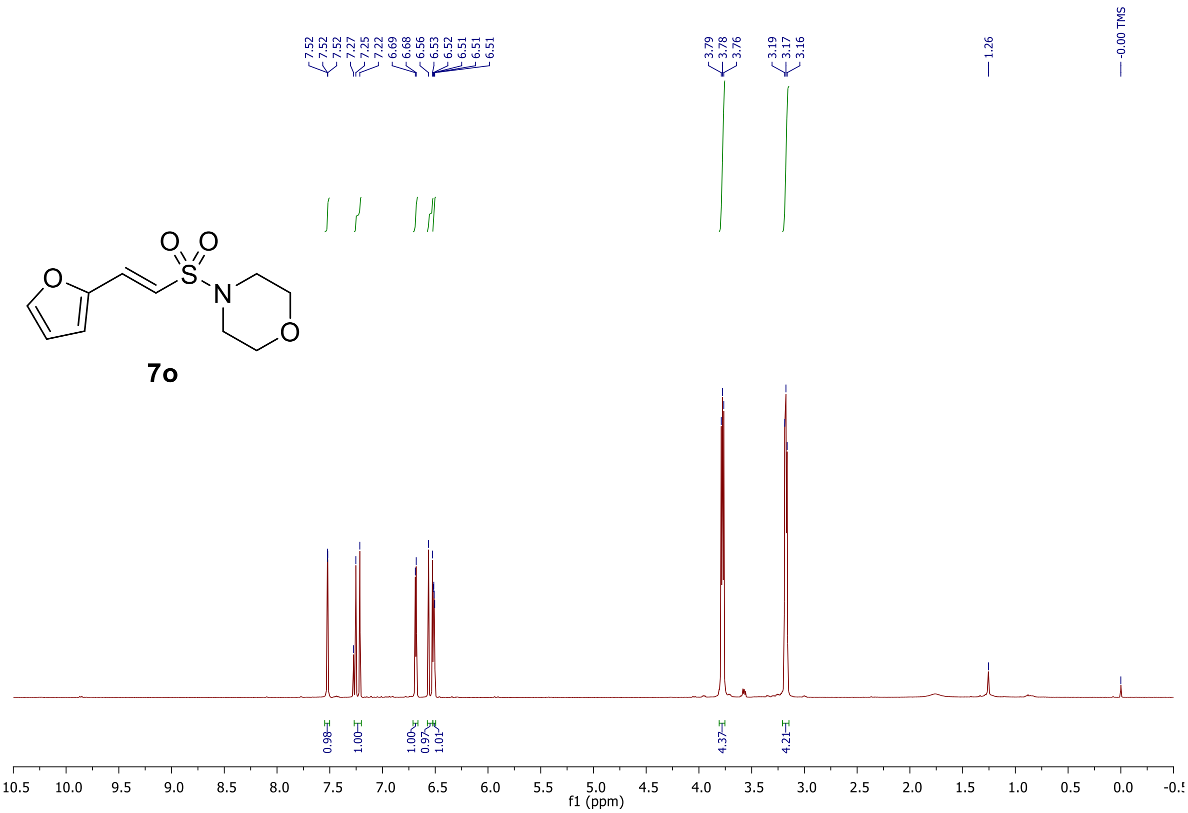

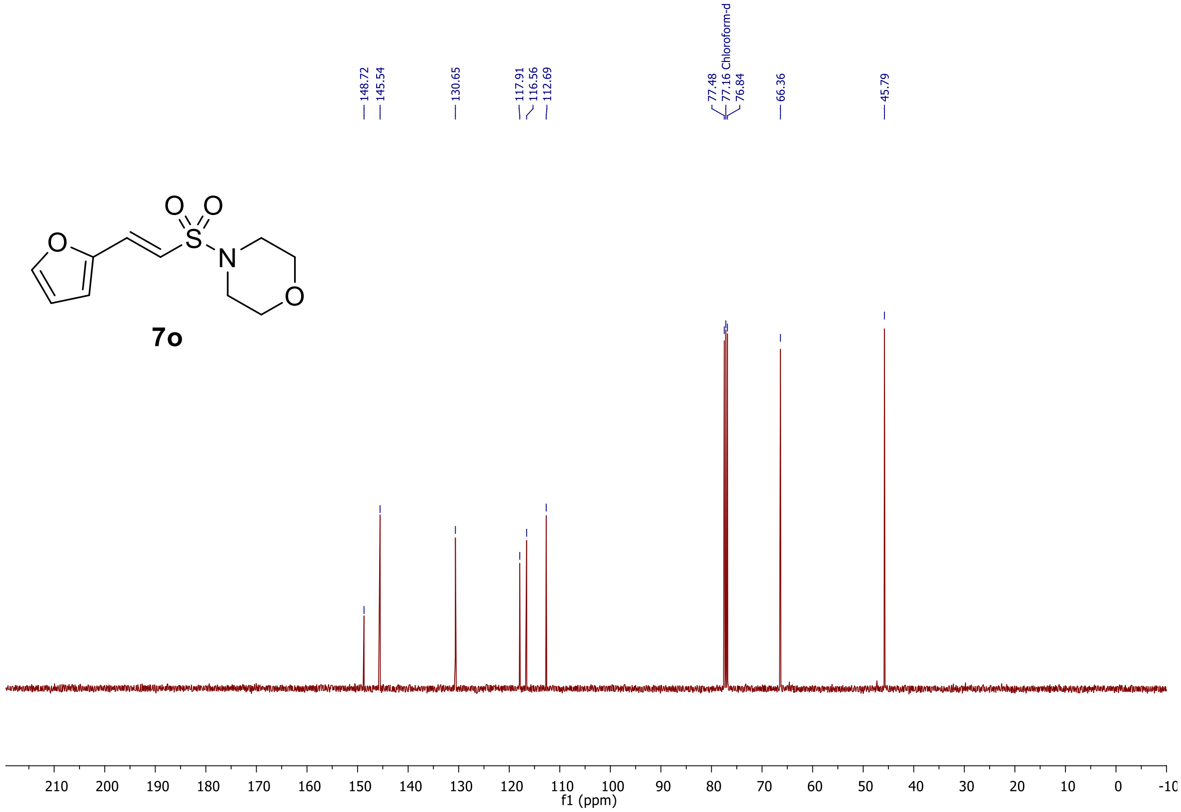

Figure S27: ^{1}H ($CDCl_3$, 400 MHz) and $^{13}C\{^{1}H\}$ ($CDCl_3$, 101 MHz) NMR Spectrum of **7o**.

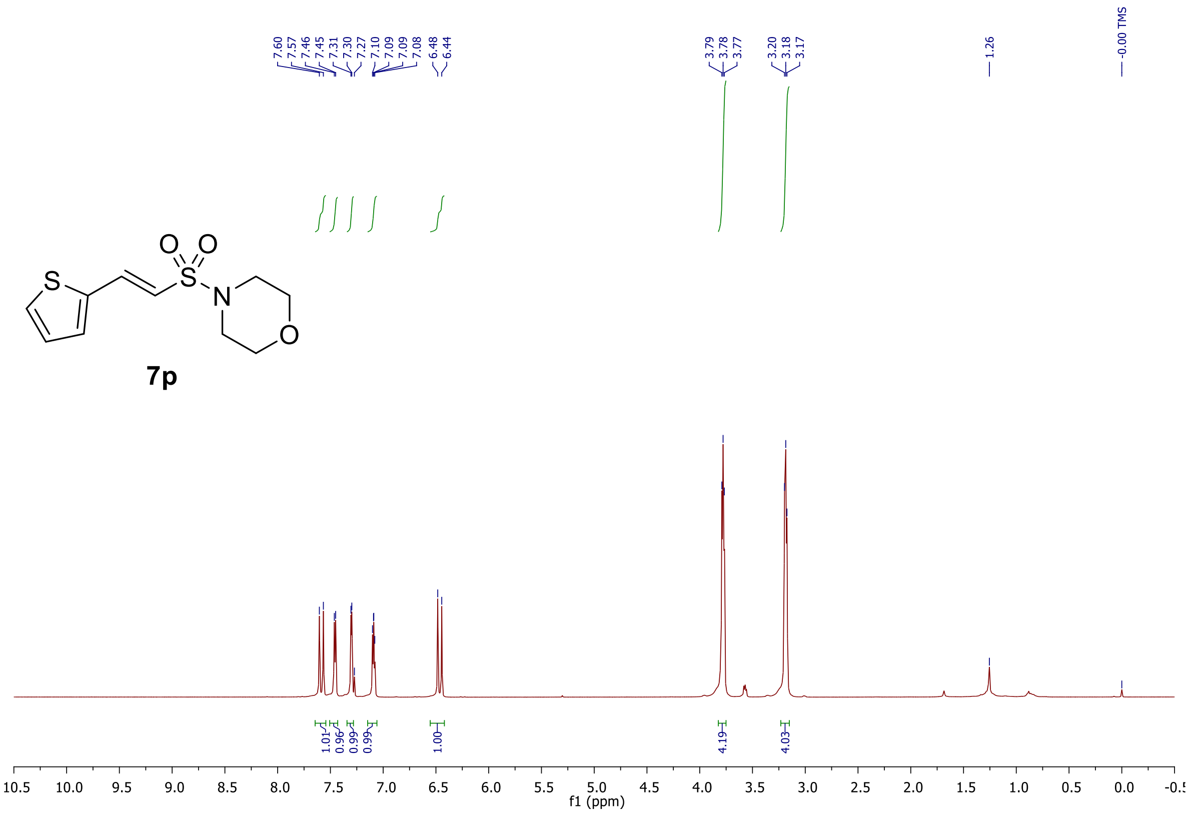

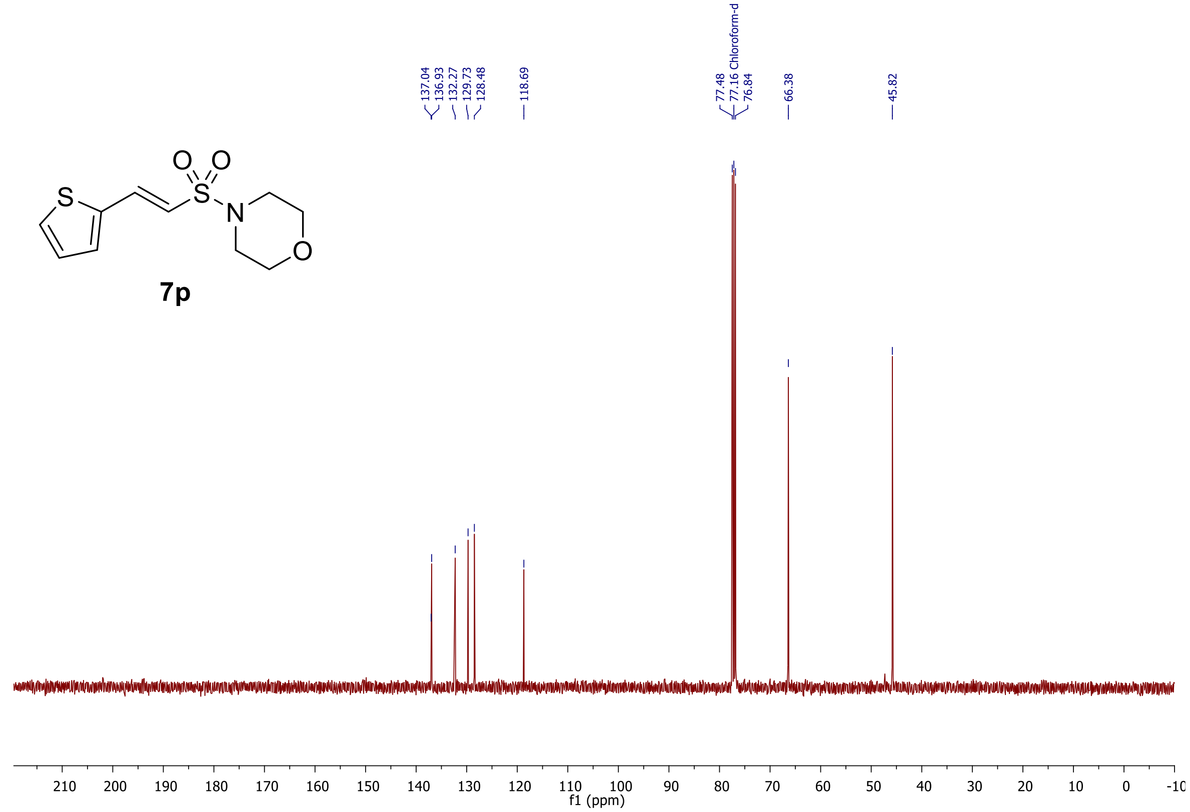

Figure S28: ^{1}H ($CDCl_3$, 400 MHz) and $^{13}C\{^{1}H\}$ ($CDCl_3$, 101 MHz) NMR Spectrum of **7p**.

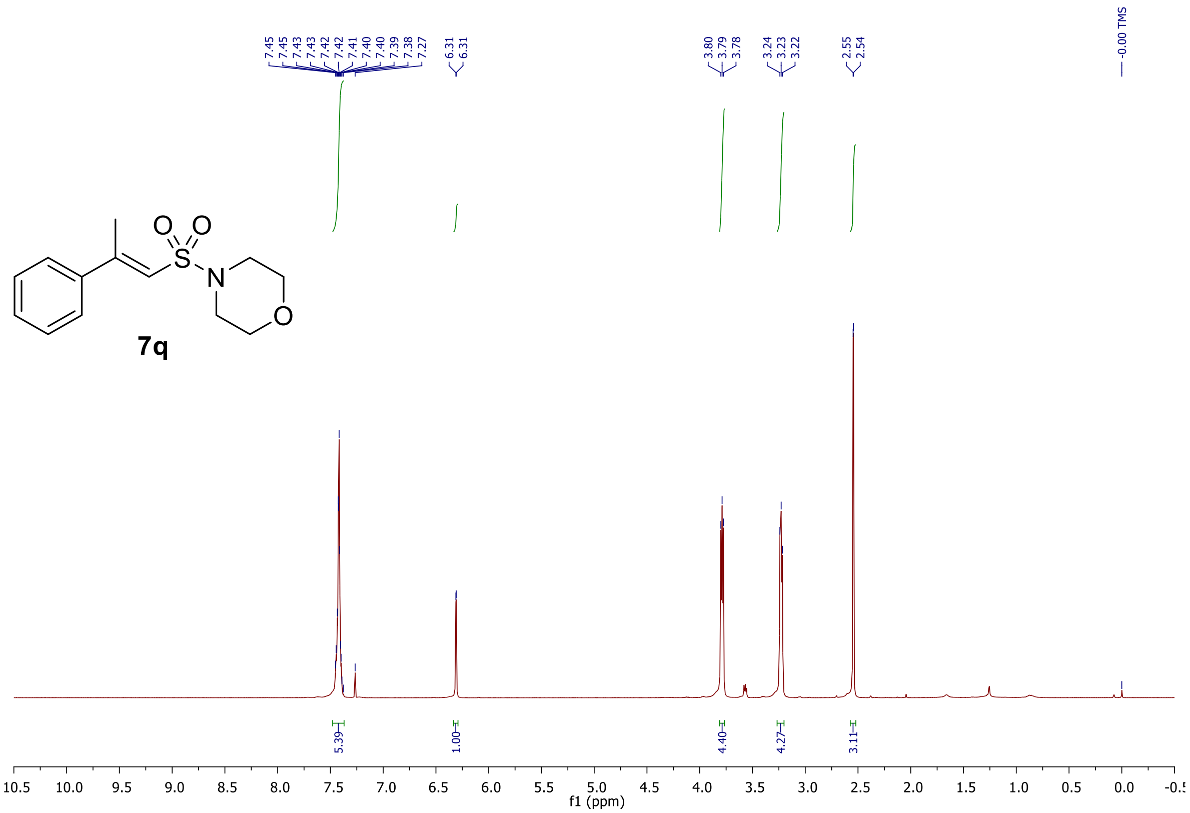

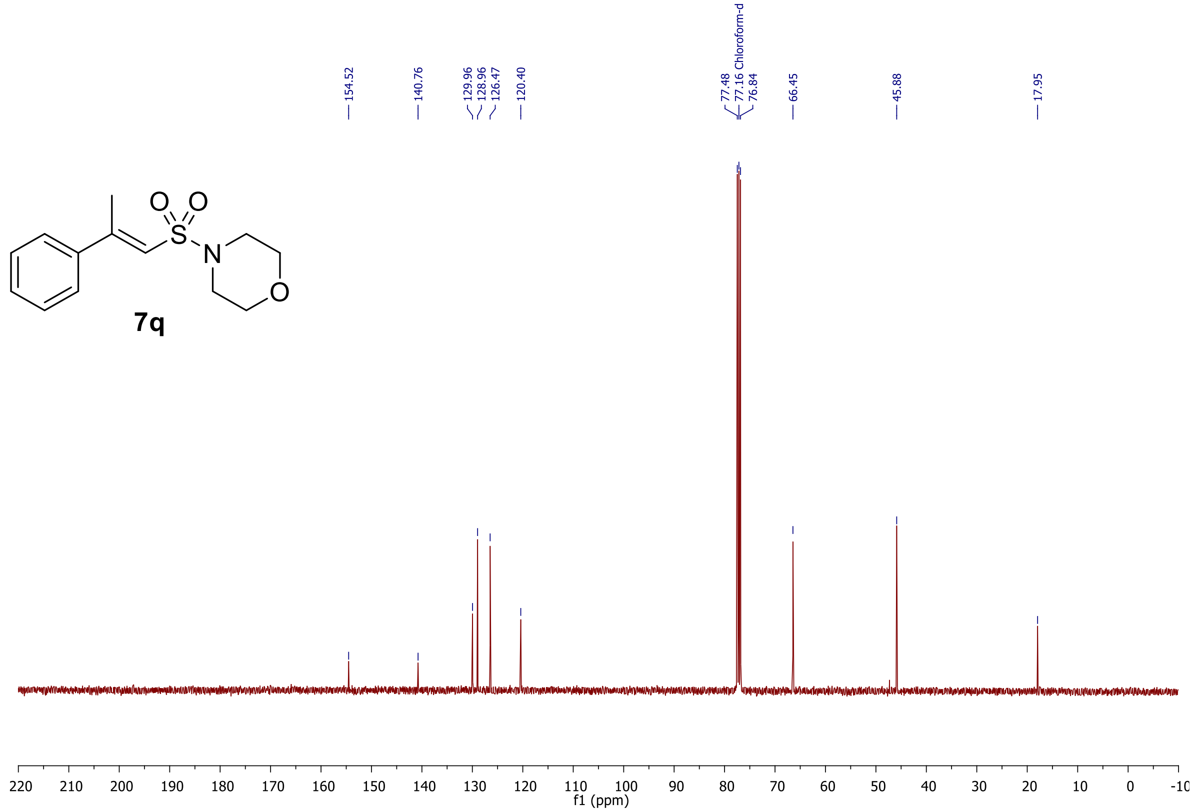

Figure S29: ^{1}H ($CDCl_3$, 400 MHz) and $^{13}C\{^{1}H\}$ ($CDCl_3$, 101 MHz) NMR Spectrum of **7q**.

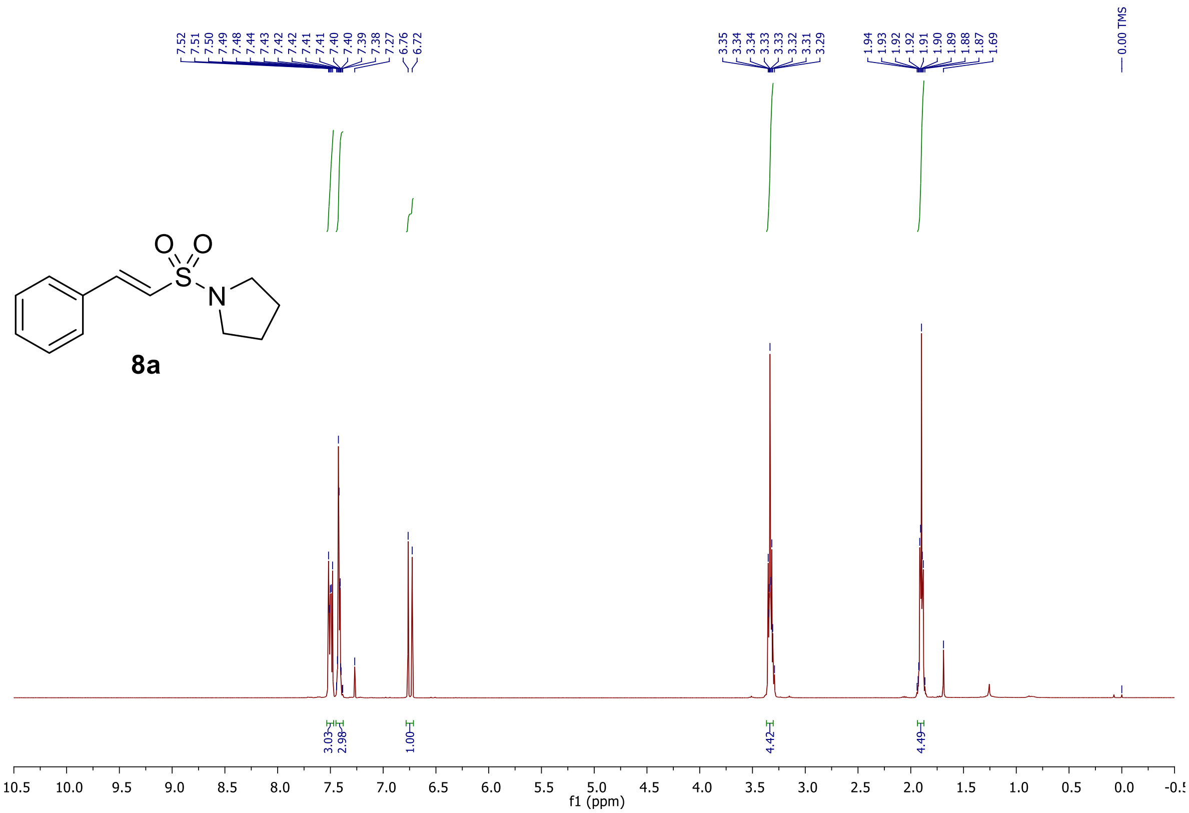

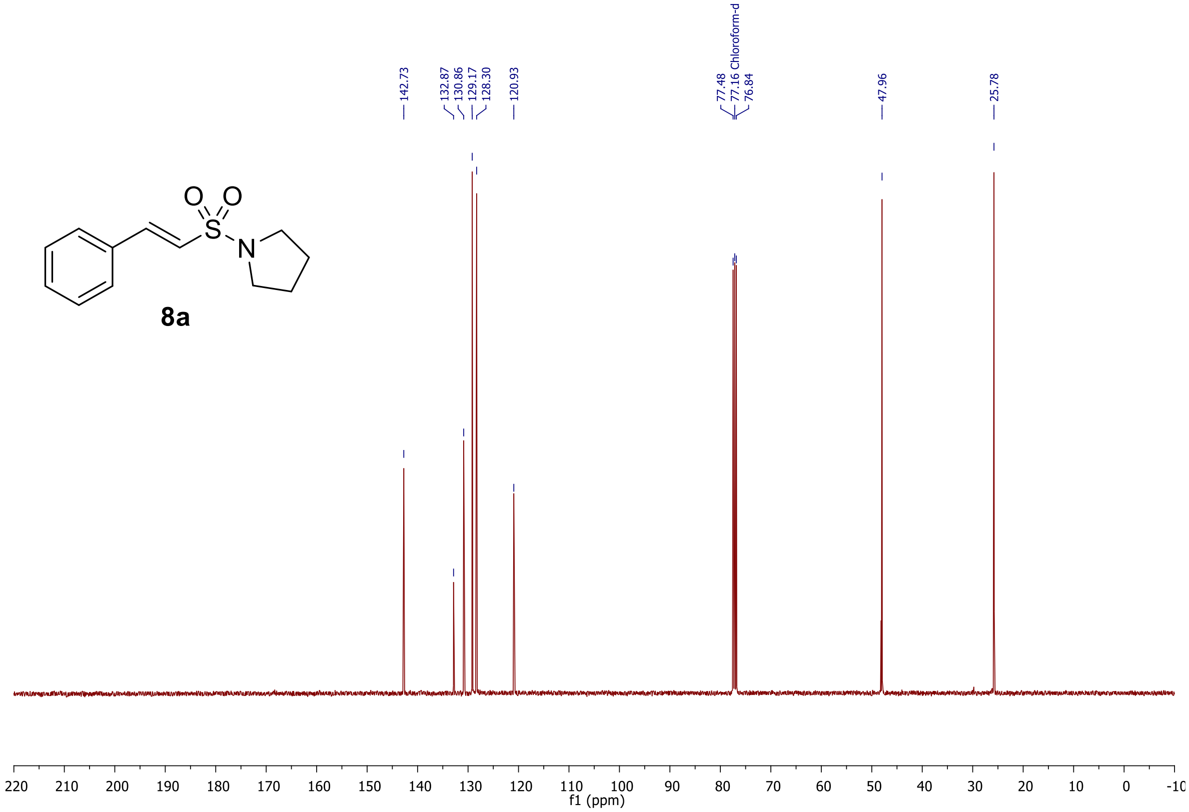

Figure S30: ^{1}H ($CDCl_3$, 400 MHz) and $^{13}C\{^{1}H\}$ ($CDCl_3$, 101 MHz) NMR Spectrum of **8a**.

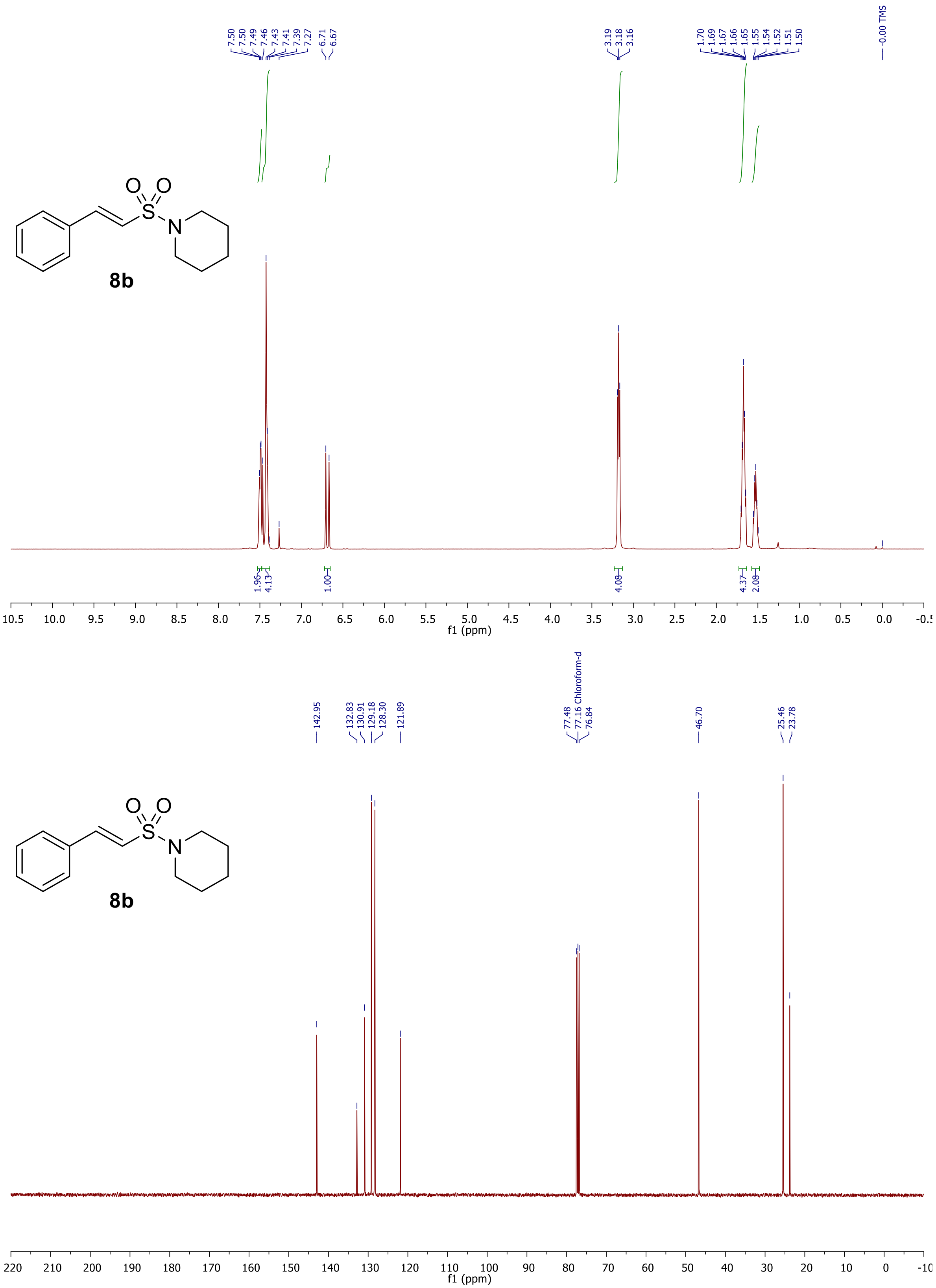

Figure S31: ^{1}H ($CDCl_3$, 400 MHz) and $^{13}C\{^{1}H\}$ ($CDCl_3$, 101 MHz) NMR Spectrum of **8b**.

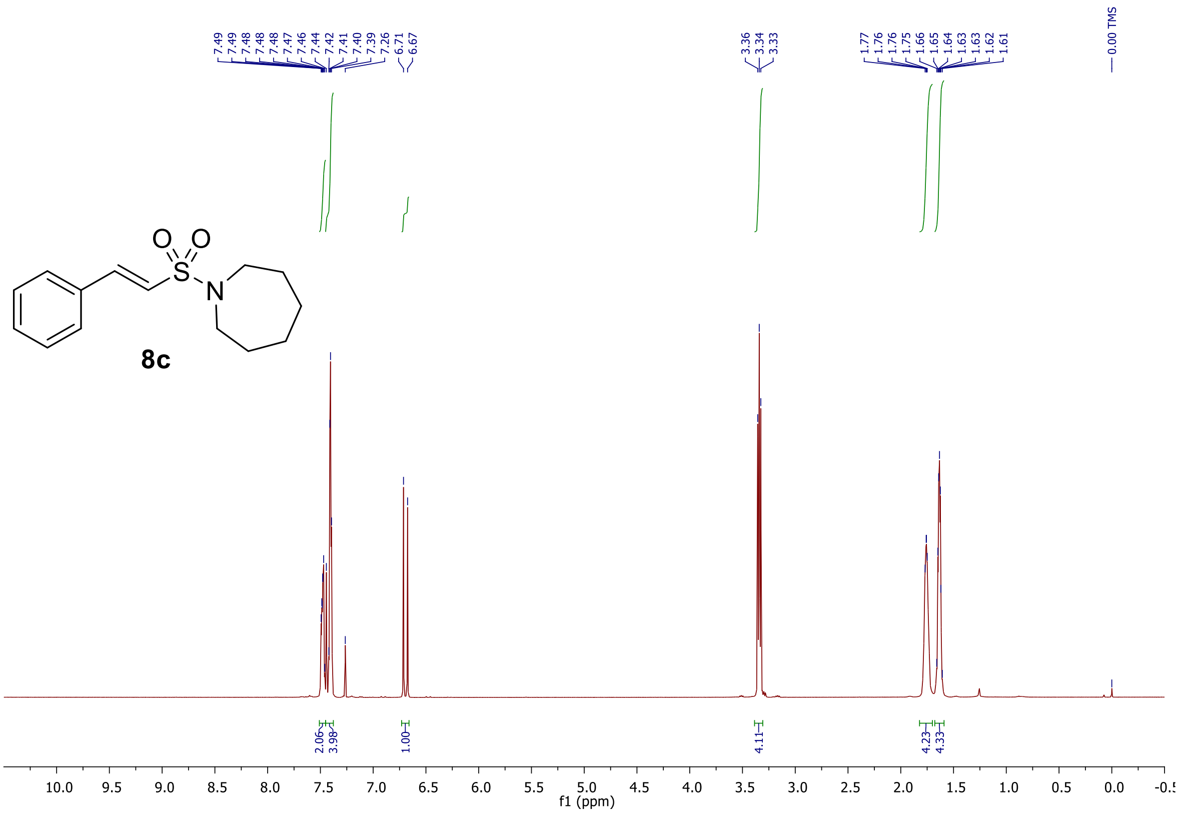

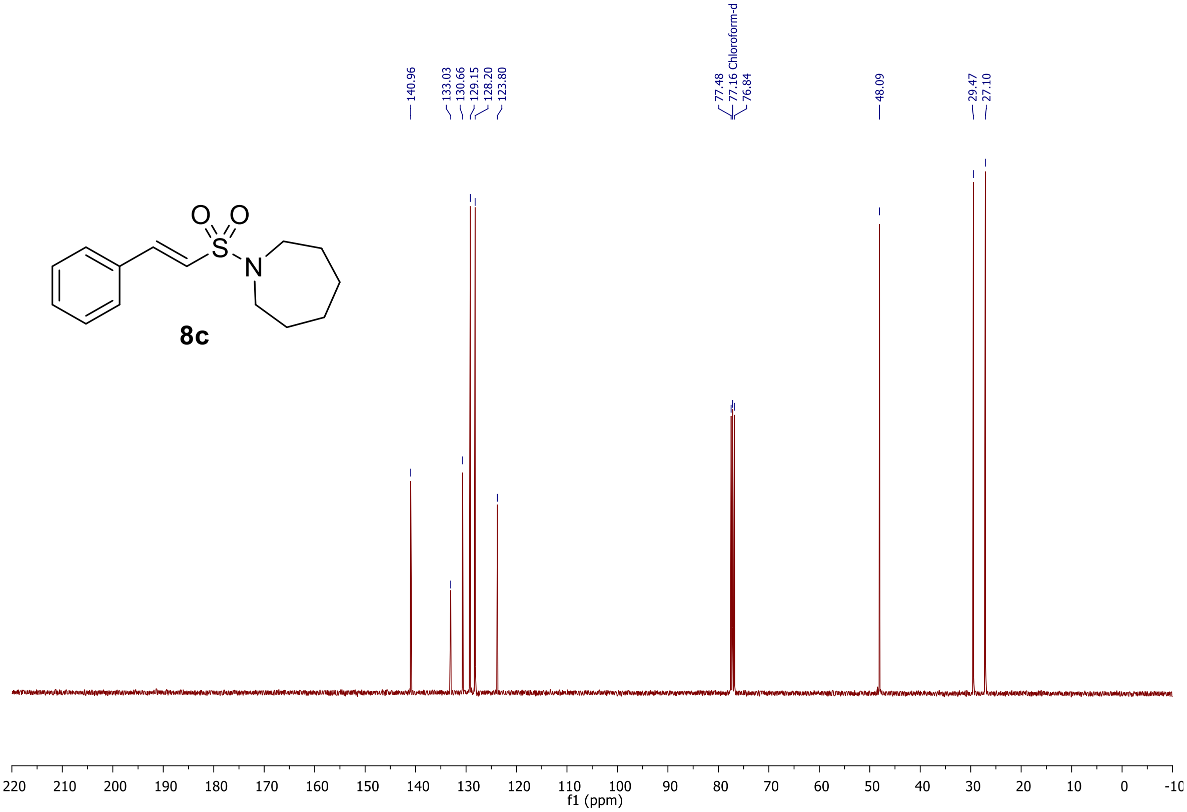

Figure S32: ^{1}H ($CDCl_3$, 400 MHz) and ^{13}C{^{1}H} ($CDCl_3$, 101 MHz) NMR Spectrum of **8c**.

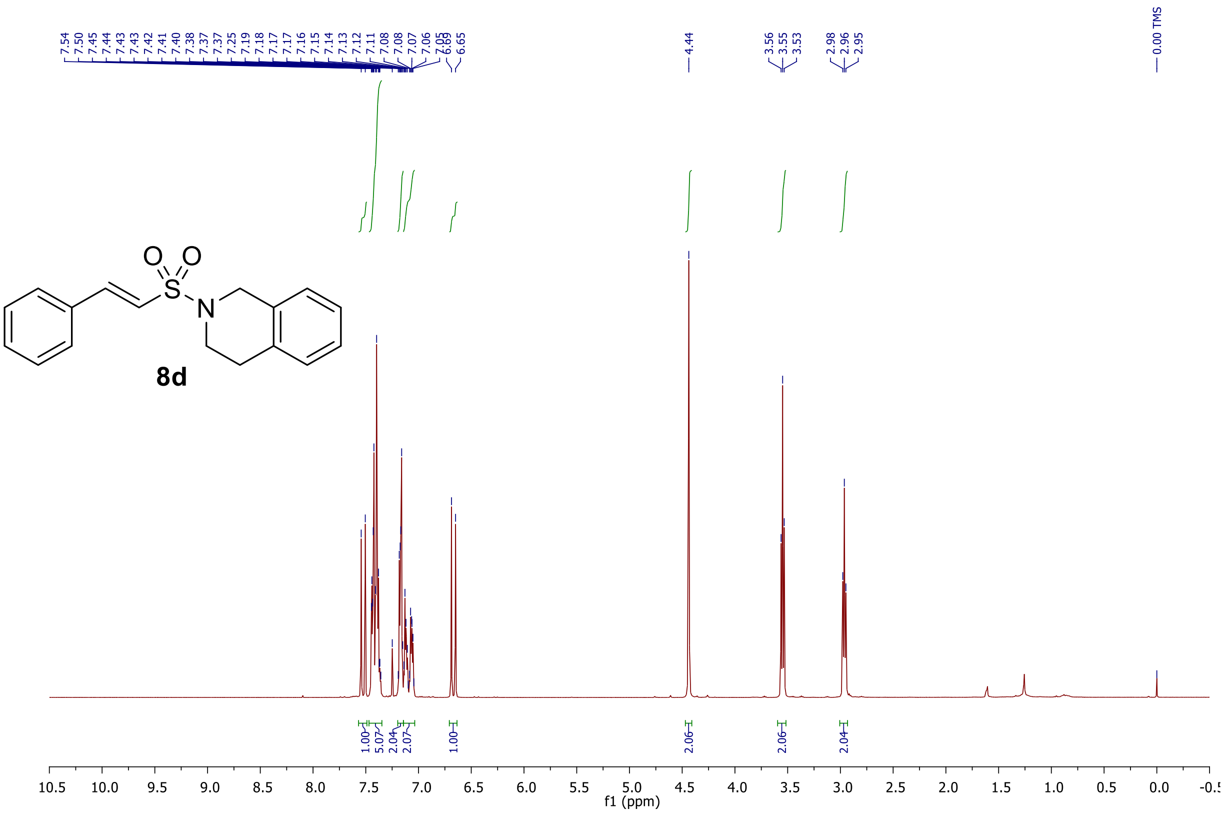

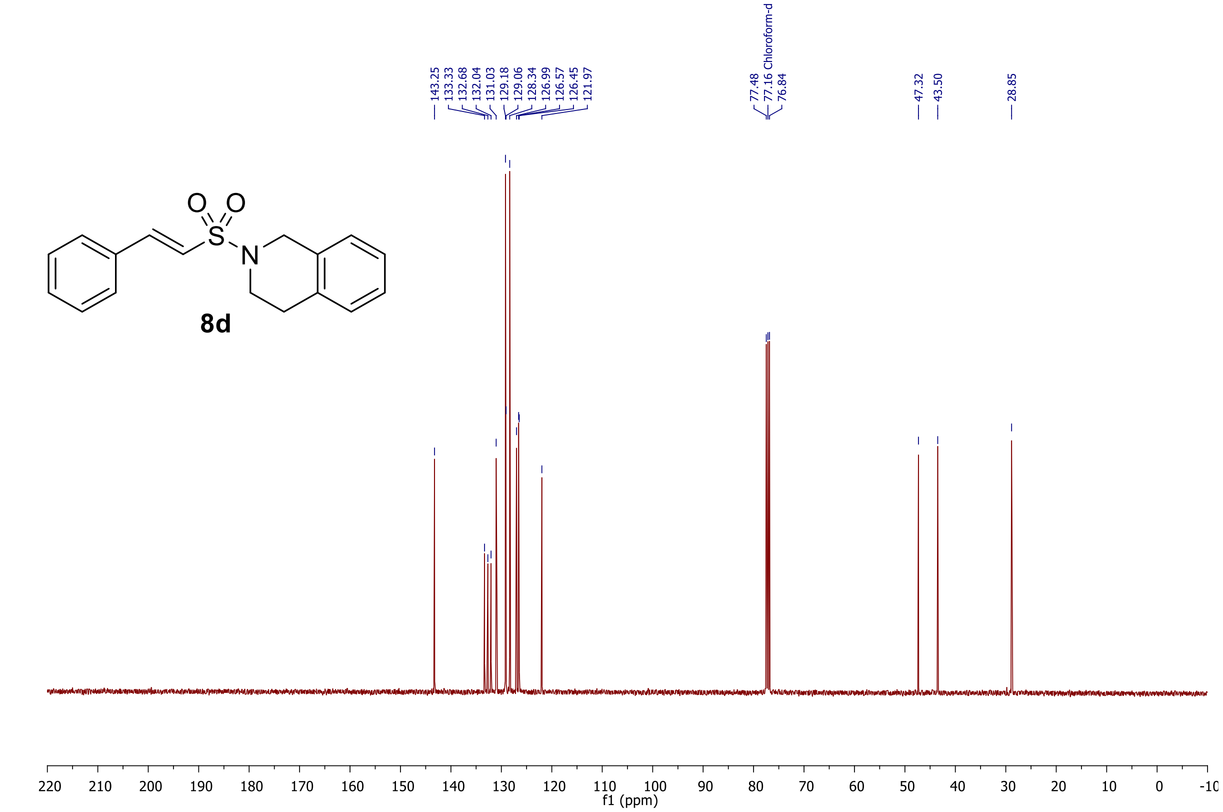

Figure S33: ^{1}H ($CDCl_3$, 400 MHz) and $^{13}C\{^{1}H\}$ ($CDCl_3$, 101 MHz) NMR Spectrum of **8d**.

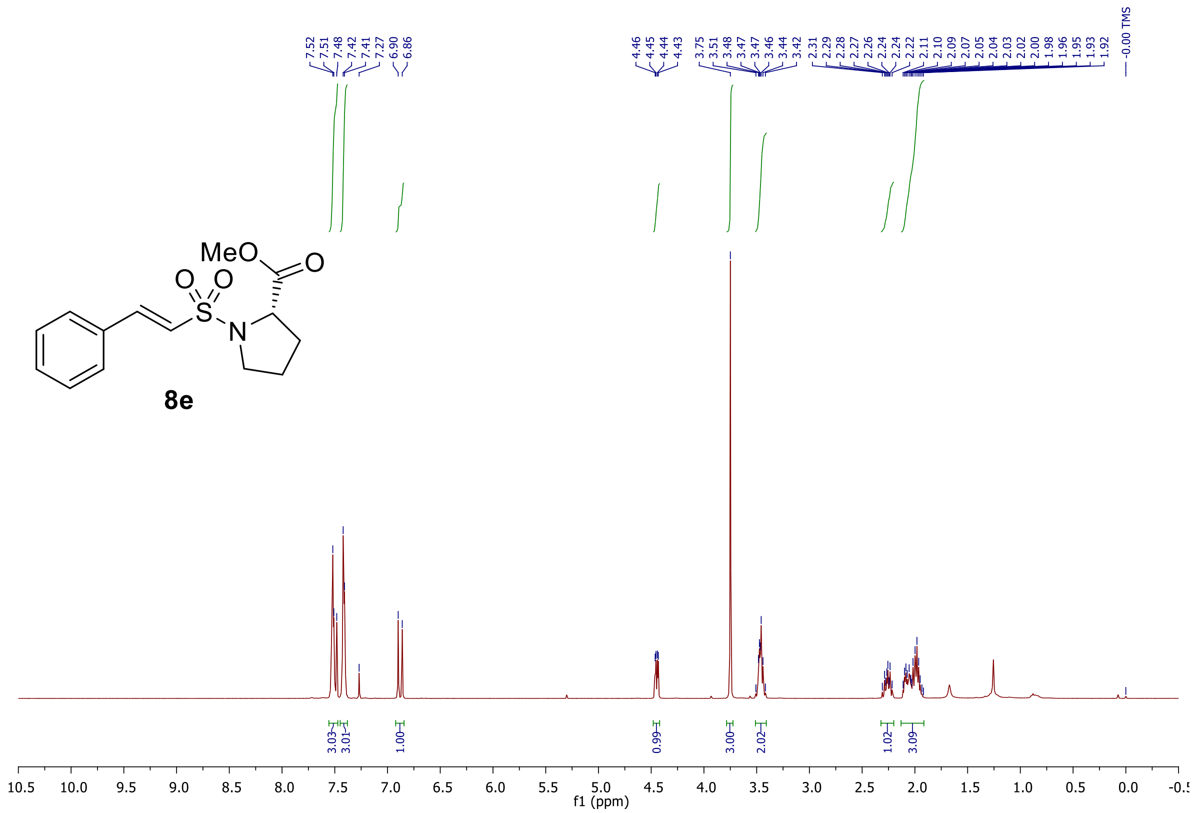

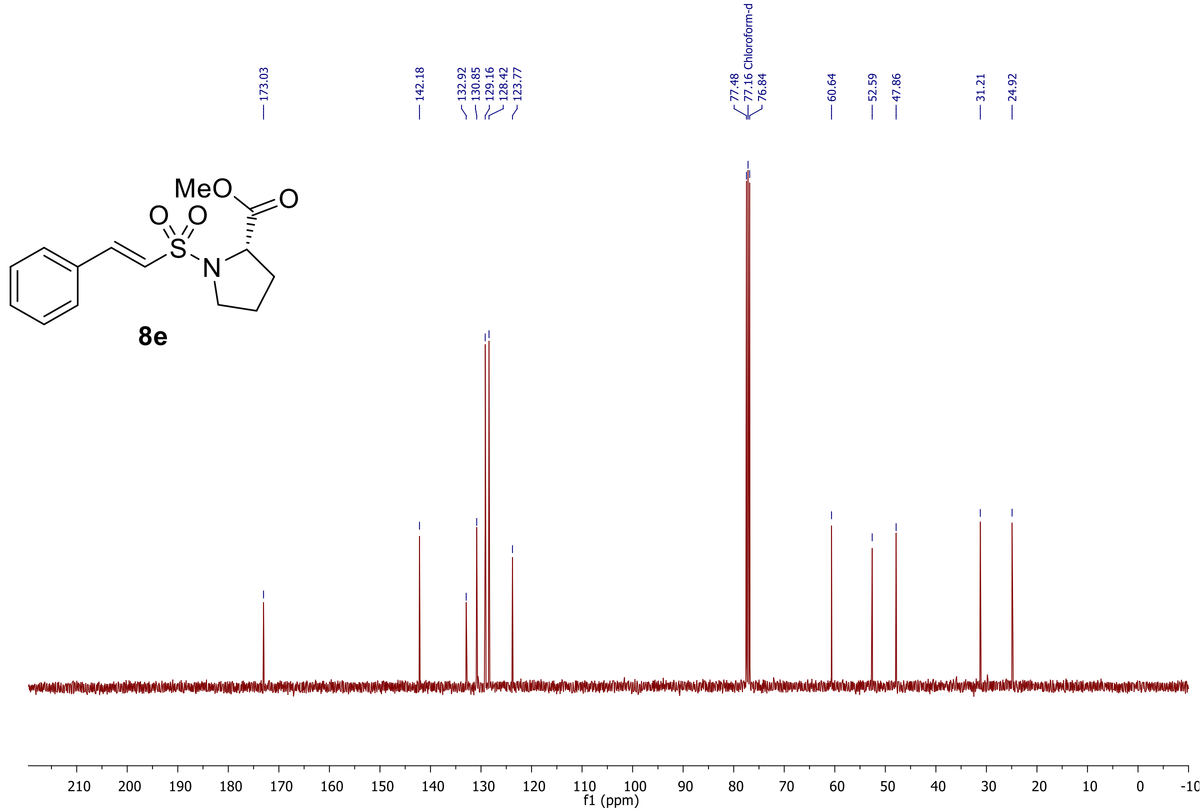

Figure S34: ^{1}H ($CDCl_3$, 400 MHz) and $^{13}C\{^{1}H\}$ ($CDCl_3$, 101 MHz) NMR Spectrum of **8e**.

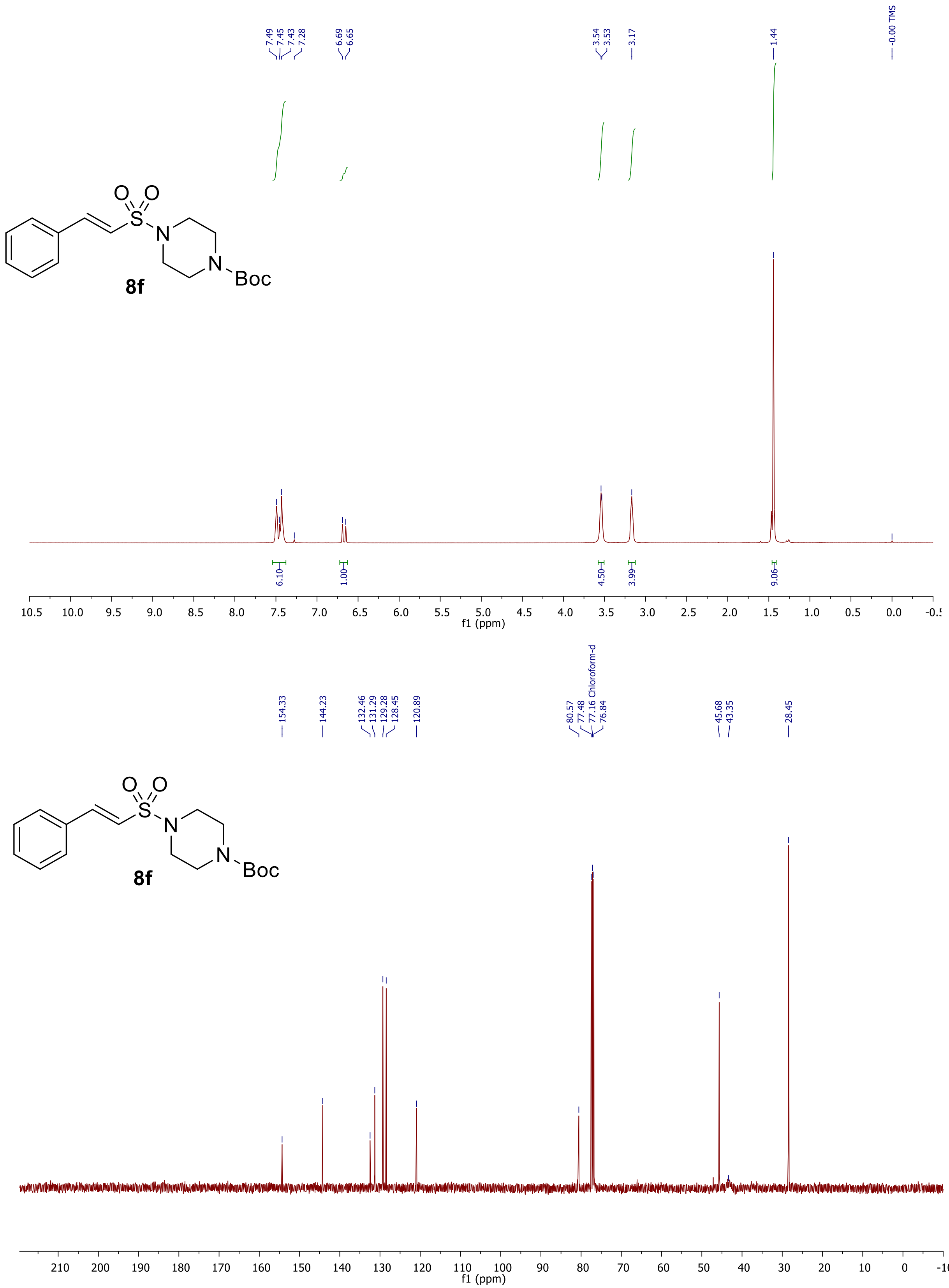

Figure S35: ^{1}H ($CDCl_3$, 400 MHz) and $^{13}C\{^{1}H\}$ ($CDCl_3$, 101 MHz) NMR Spectrum of **8f**.

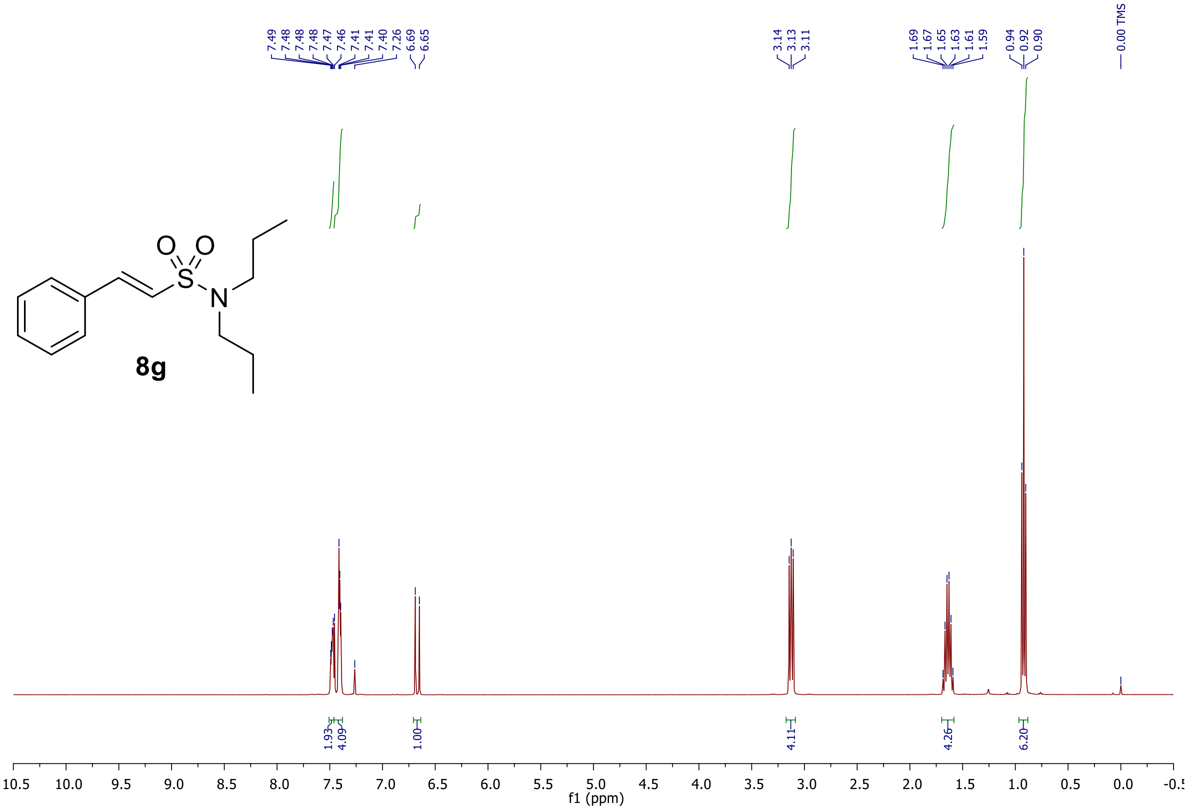

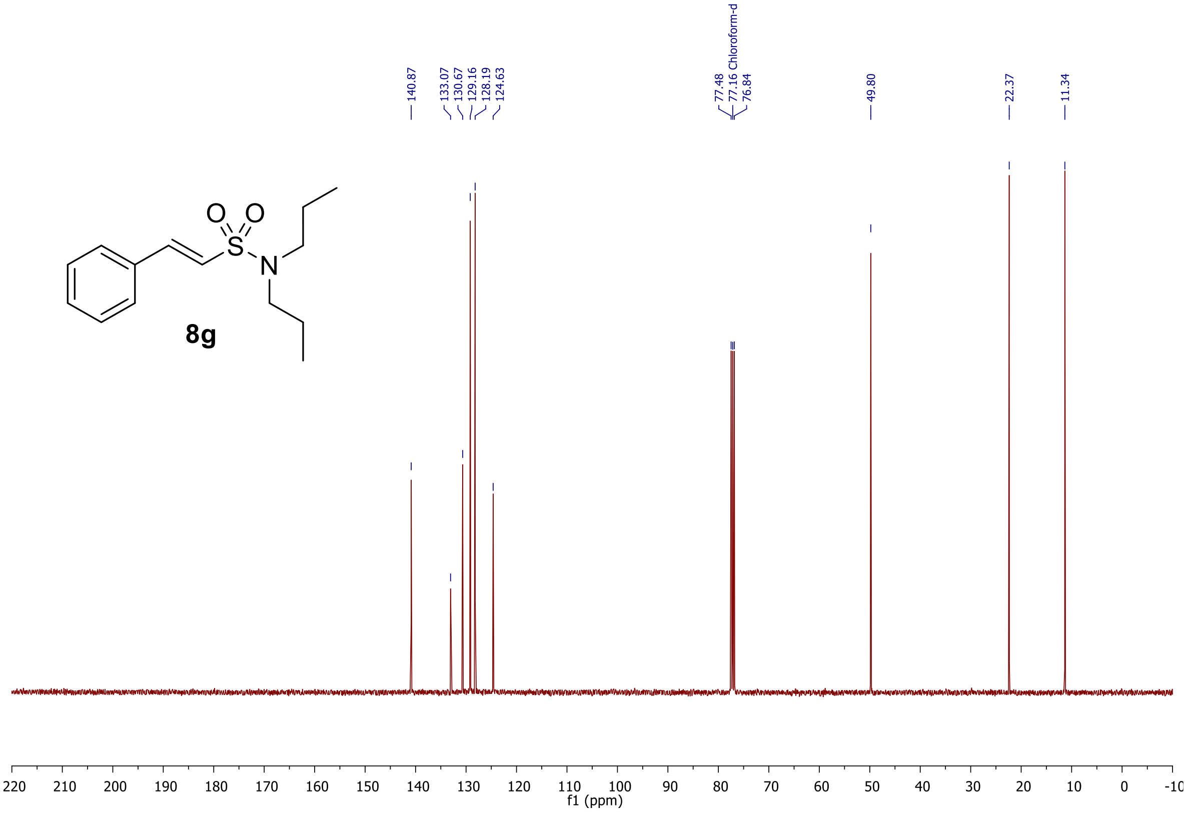

Figure S36: ^{1}H ($CDCl_3$, 400 MHz) and $^{13}C\{^{1}H\}$ ($CDCl_3$, 101 MHz) NMR Spectrum of **8g**.

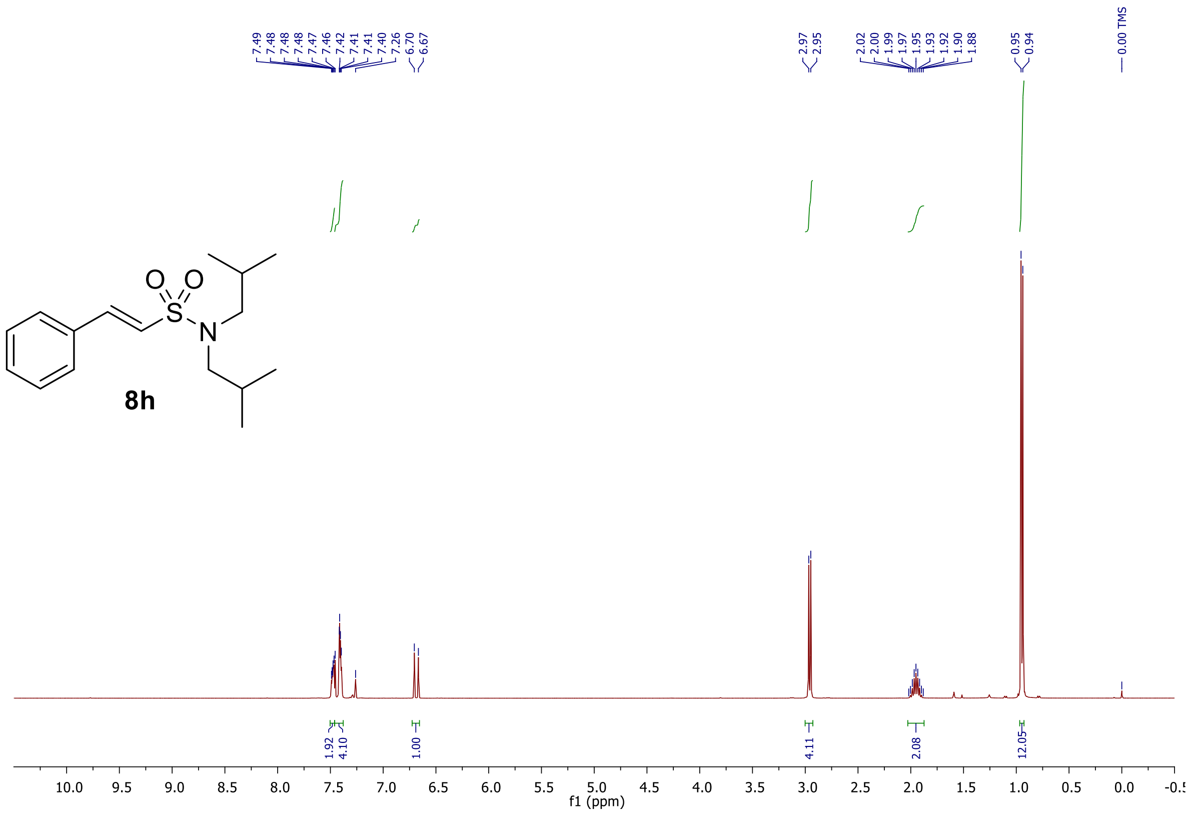

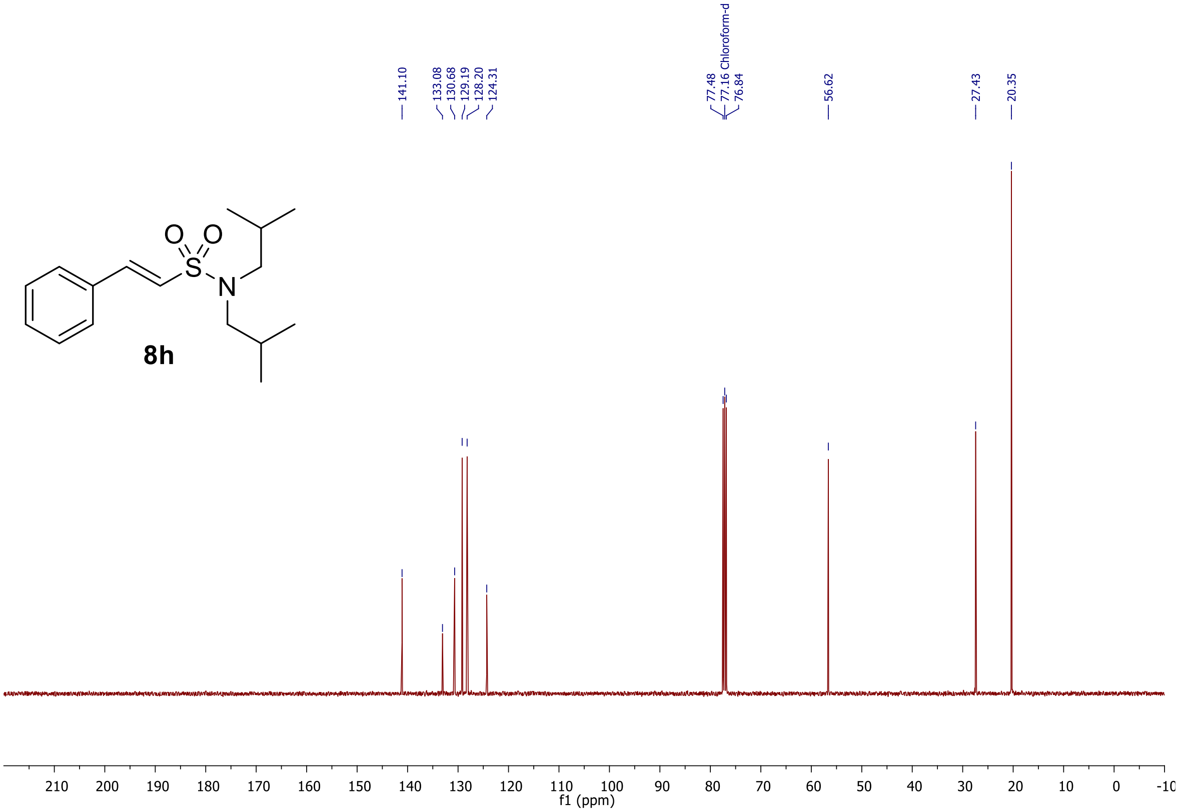

Figure S37: ^{1}H ($CDCl_3$, 400 MHz) and $^{13}C\{^{1}H\}$ ($CDCl_3$, 101 MHz) NMR Spectrum of **8h**.

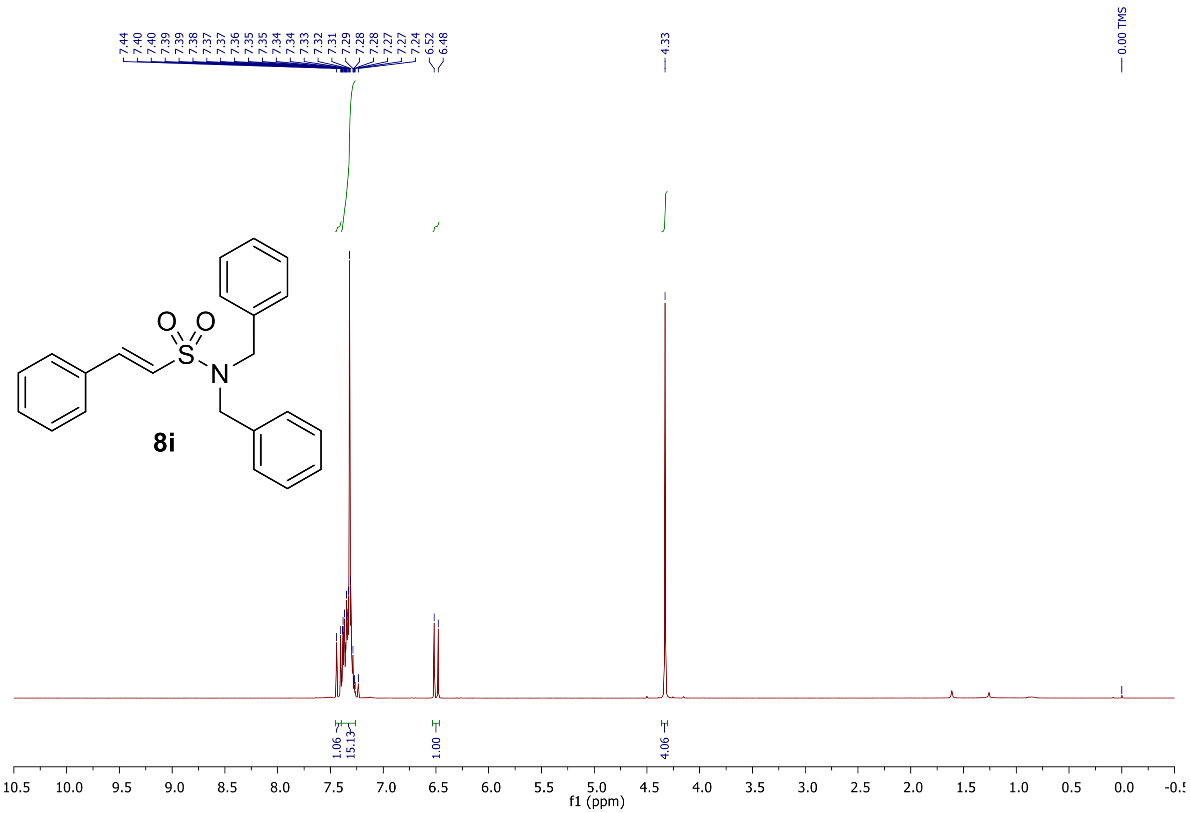

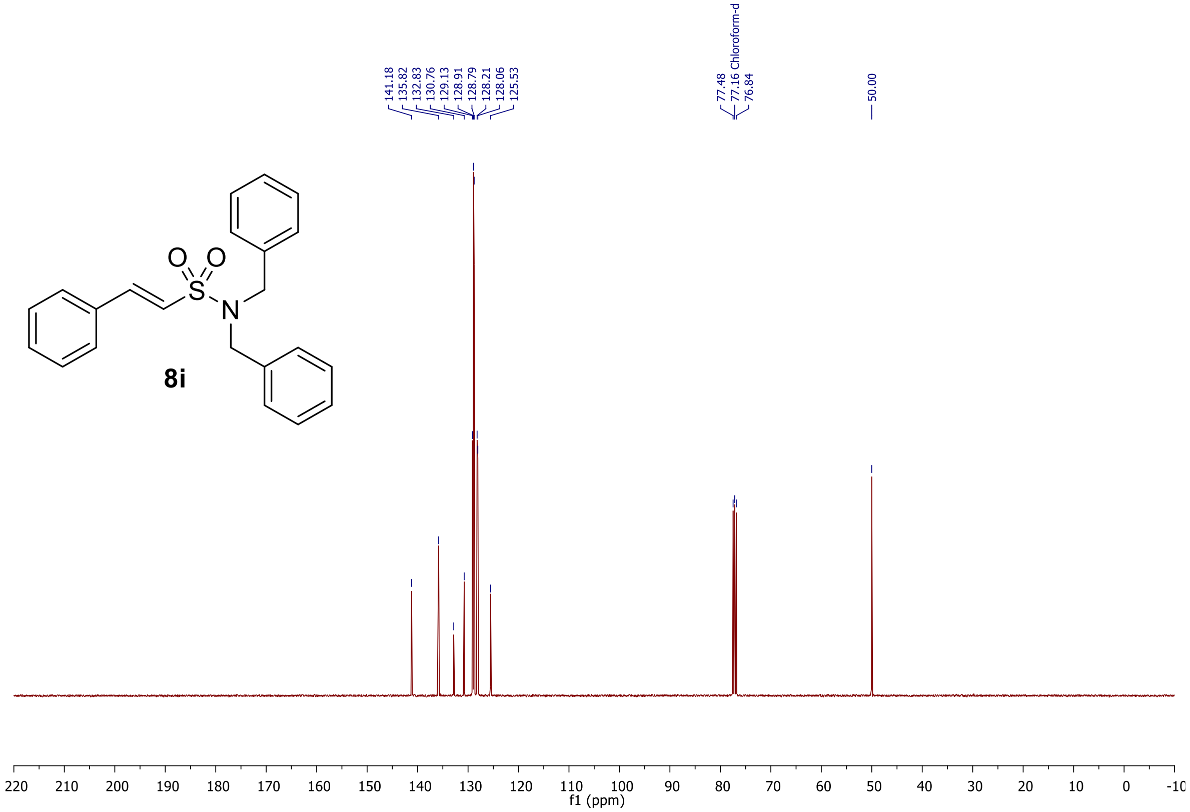

Figure S38: ^{1}H ($CDCl_3$, 400 MHz) and $^{13}C\{^1H\}$ ($CDCl_3$, 101 MHz) NMR Spectrum of **8i**.

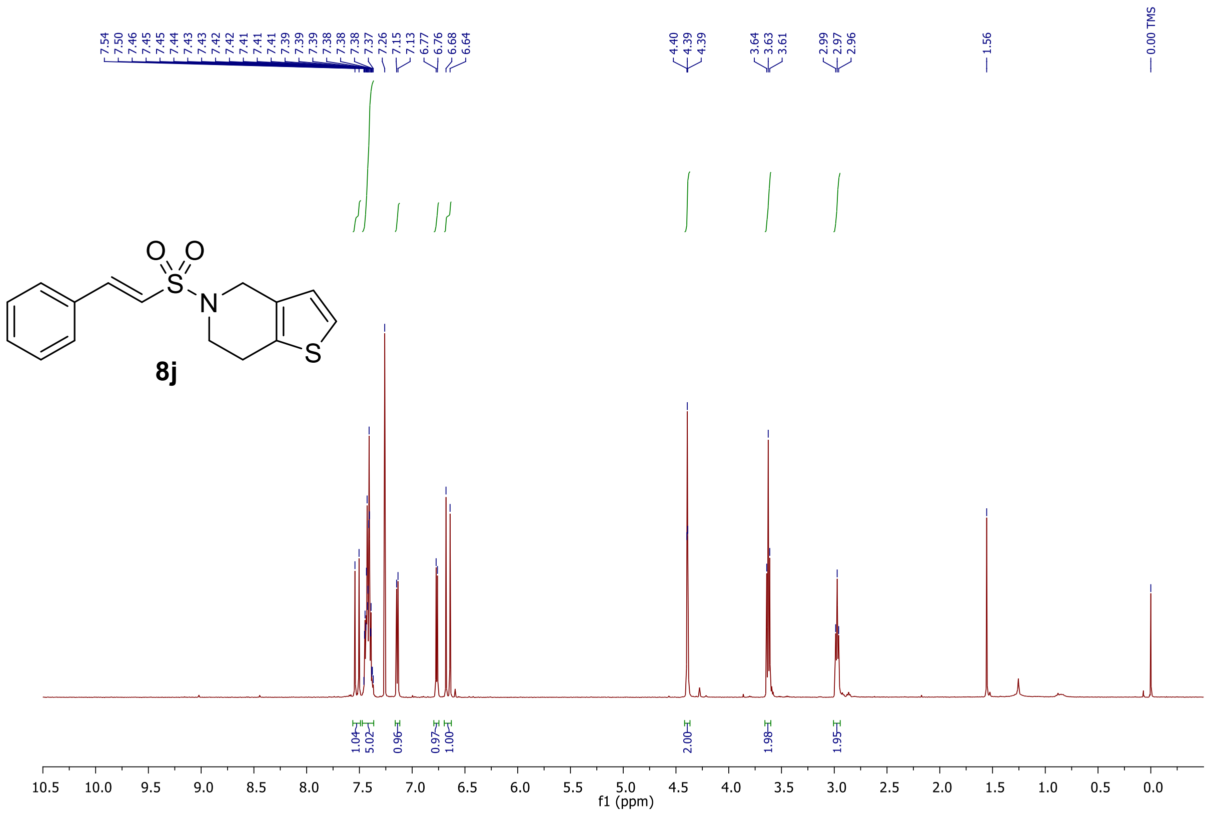

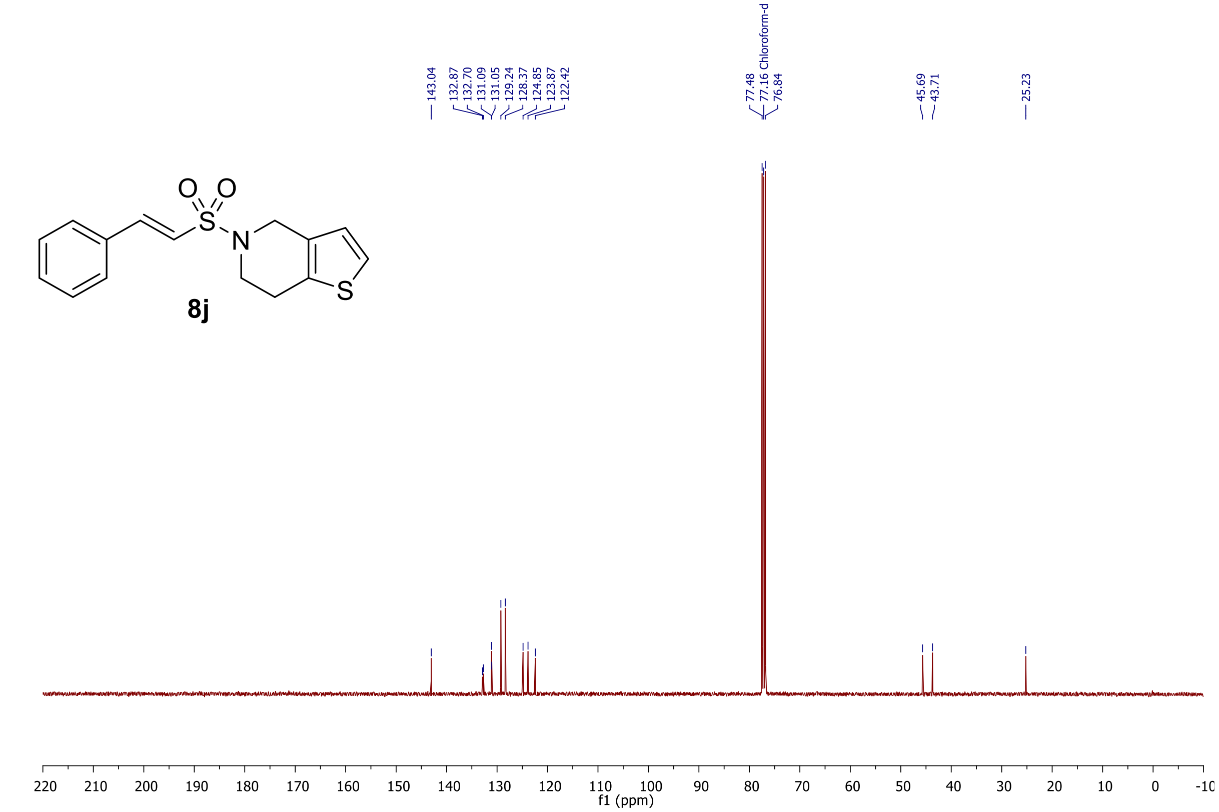

Figure S39: ^{1}H ($CDCl_3$, 400 MHz) and $^{13}C\{^{1}H\}$ ($CDCl_3$, 101 MHz) NMR Spectrum of **8j**.

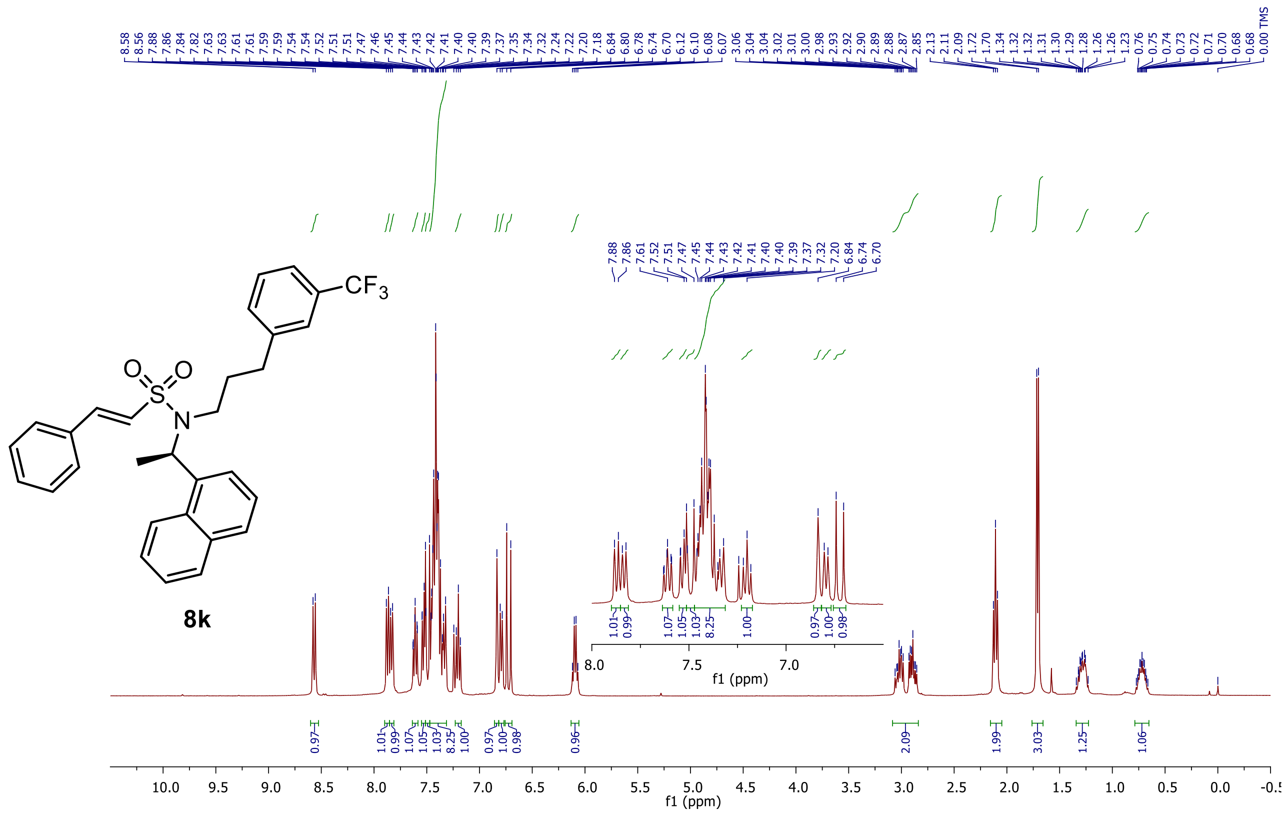

8.58 8.56 7.88 7.86 7.84 7.82 7.63 7.61 7.61 7.59 7.54 7.54 7.52 7.51 7.51 7.47 7.46 7.45 7.44 7.43 7.42 7.42 7.41 7.40 7.39 7.37 7.35 7.34 7.32 7.24 7.22 7.20 7.18 6.84 6.80 6.78 6.74 6.70 6.12 6.10 6.08 6.07 3.06 3.04 3.04 3.02 3.01 3.00 2.98 2.93 2.92 2.90 2.89 2.88 2.87 2.85 2.13 2.11 2.09 1.72 1.70 1.34 1.32 1.32 1.31 1.30 1.29 1.28 1.26 1.23 0.76 0.75 0.74 0.73 0.72 0.71 0.70 0.68 0.00 TMS
CF3
8k
0.97 1.01 0.99 1.07 1.05 1.03 8.25 1.00 0.97 1.00 0.98 0.96 2.09 1.99 3.03 1.25 1.06
f1 (ppm)

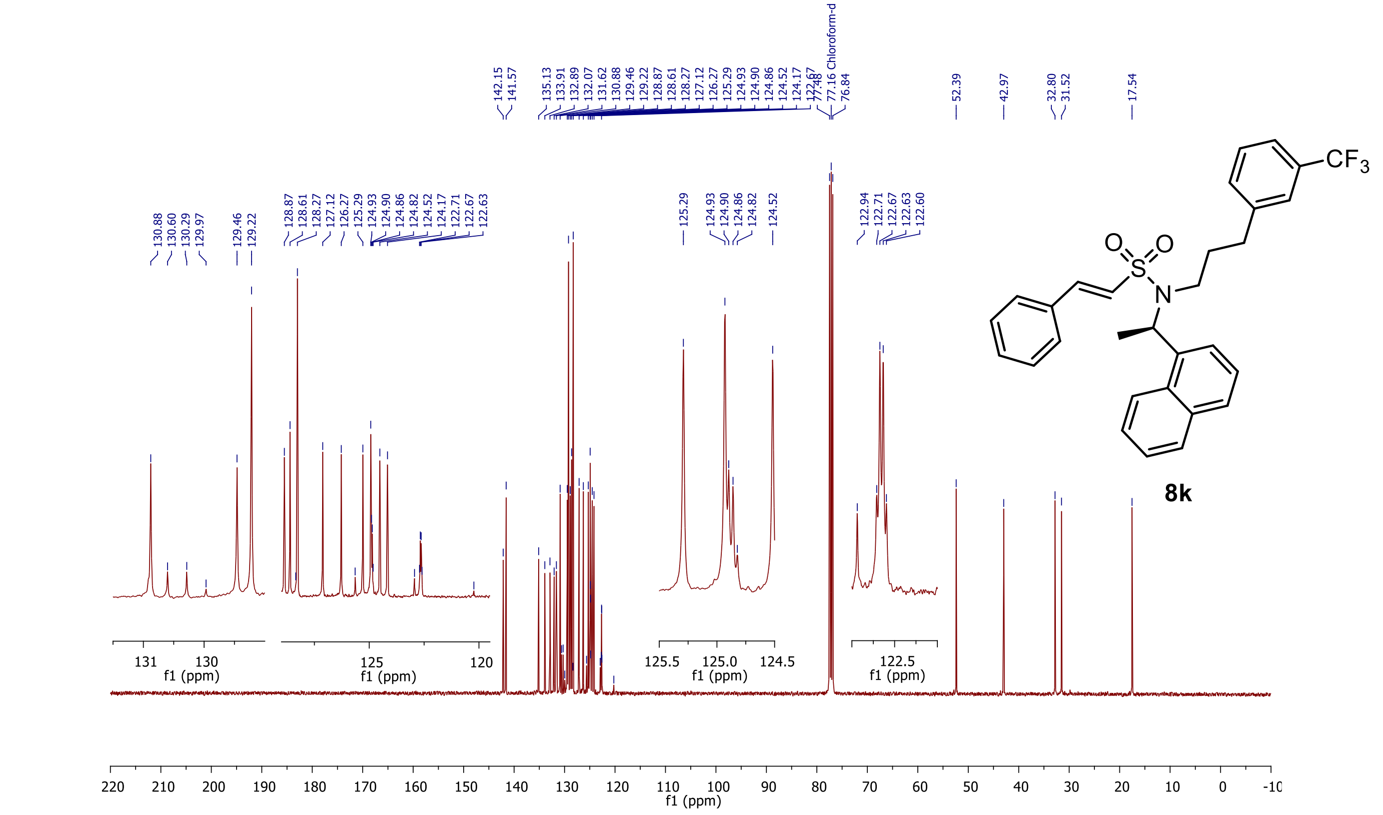

142.15 141.57 135.13 133.91 132.89 132.07 131.62 130.88 129.46 129.22 128.87 128.61 128.27 127.12 126.27 125.29 124.93 124.90 124.86 124.52 124.17 122.87 77.48 77.16 Chloroform-d 76.84 52.39 42.97 32.80 31.52 17.54
130.88 130.60 130.29 129.97 129.46 129.22
128.87 128.61 128.27 127.12 126.27 125.29 124.93 124.90 124.86 124.82 124.52 124.17 122.71 122.67 122.63
125.29 124.93 124.90 124.86 124.82 124.52
122.94 122.71 122.67 122.63 122.60
CF3
8k
f1 (ppm)

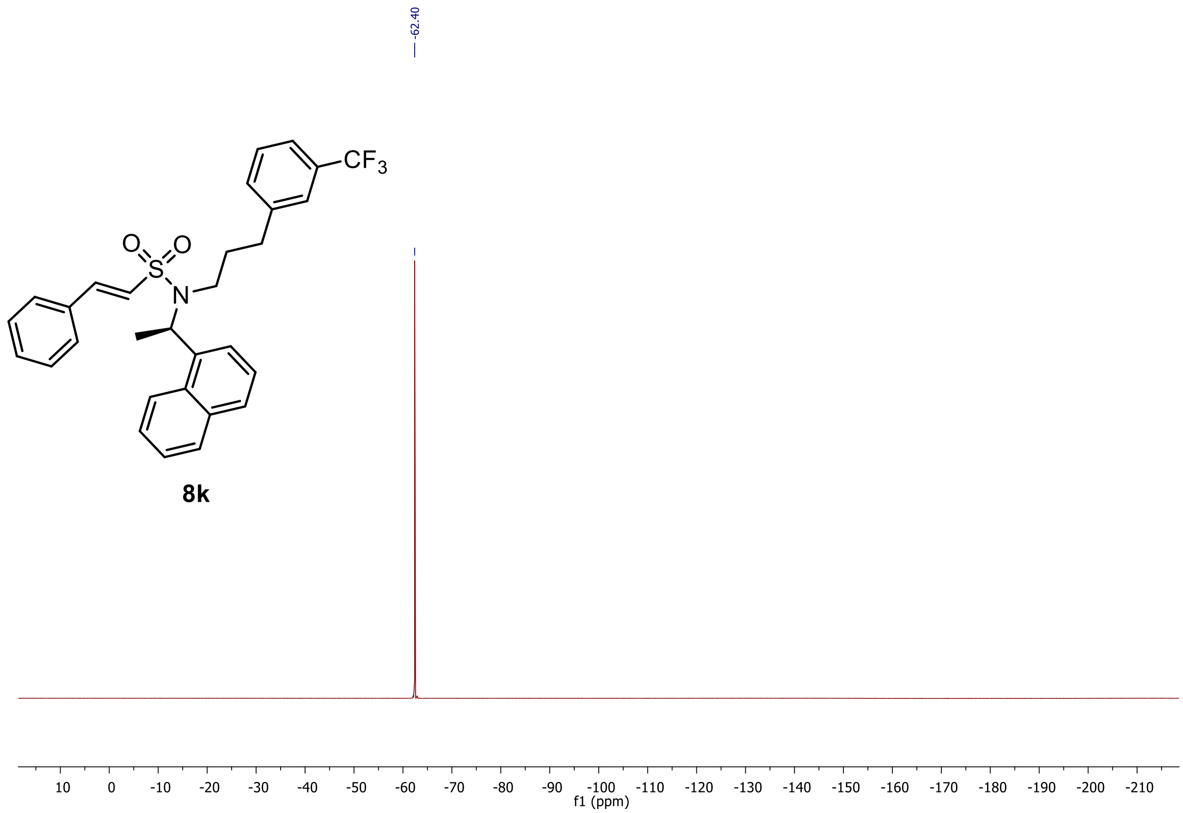

Figure S40: ^{1}H ($CDCl_3$, 400 MHz), $^{13}C\{^{1}H\}$ ($CDCl_3$, 101 MHz), and $^{19}F\{^{1}H\}$ ($CDCl_3$, 376 MHz) NMR Spectrum of **8k**.

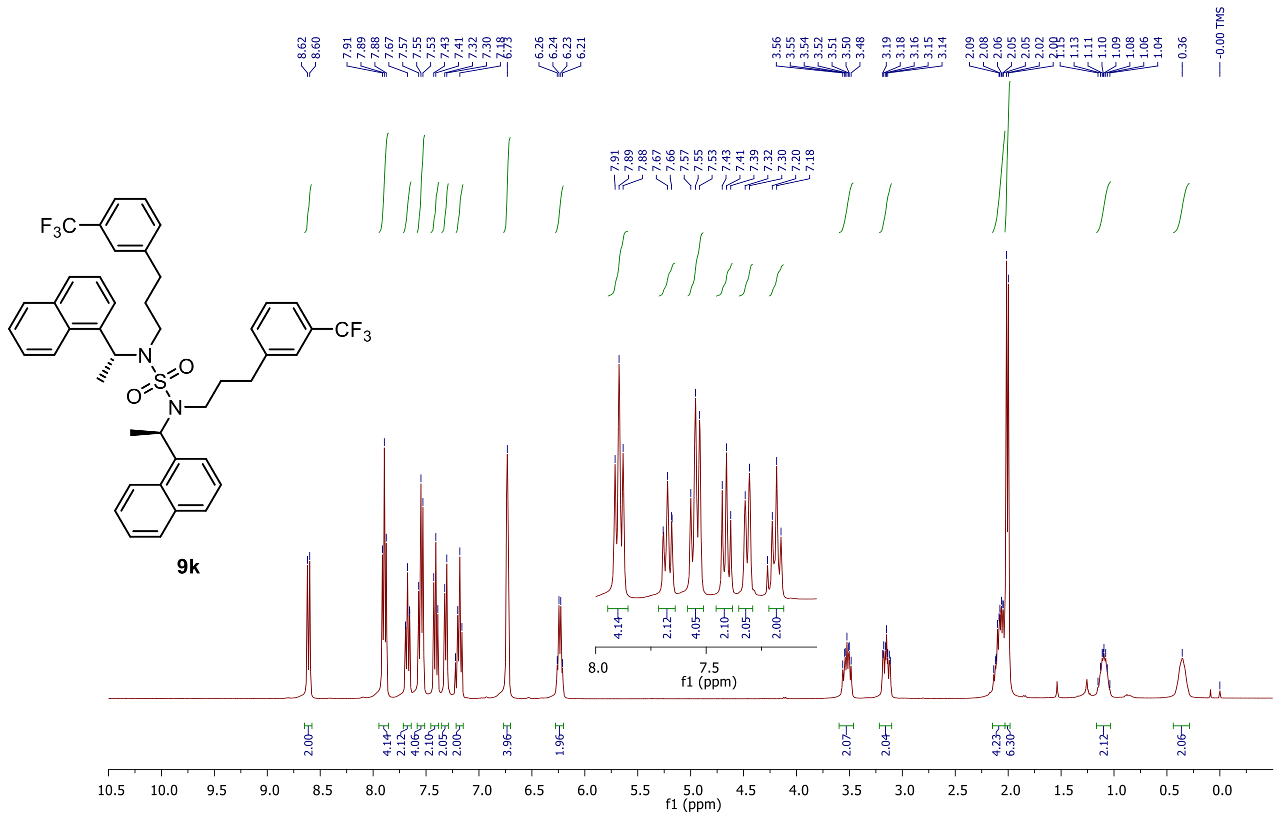

9k

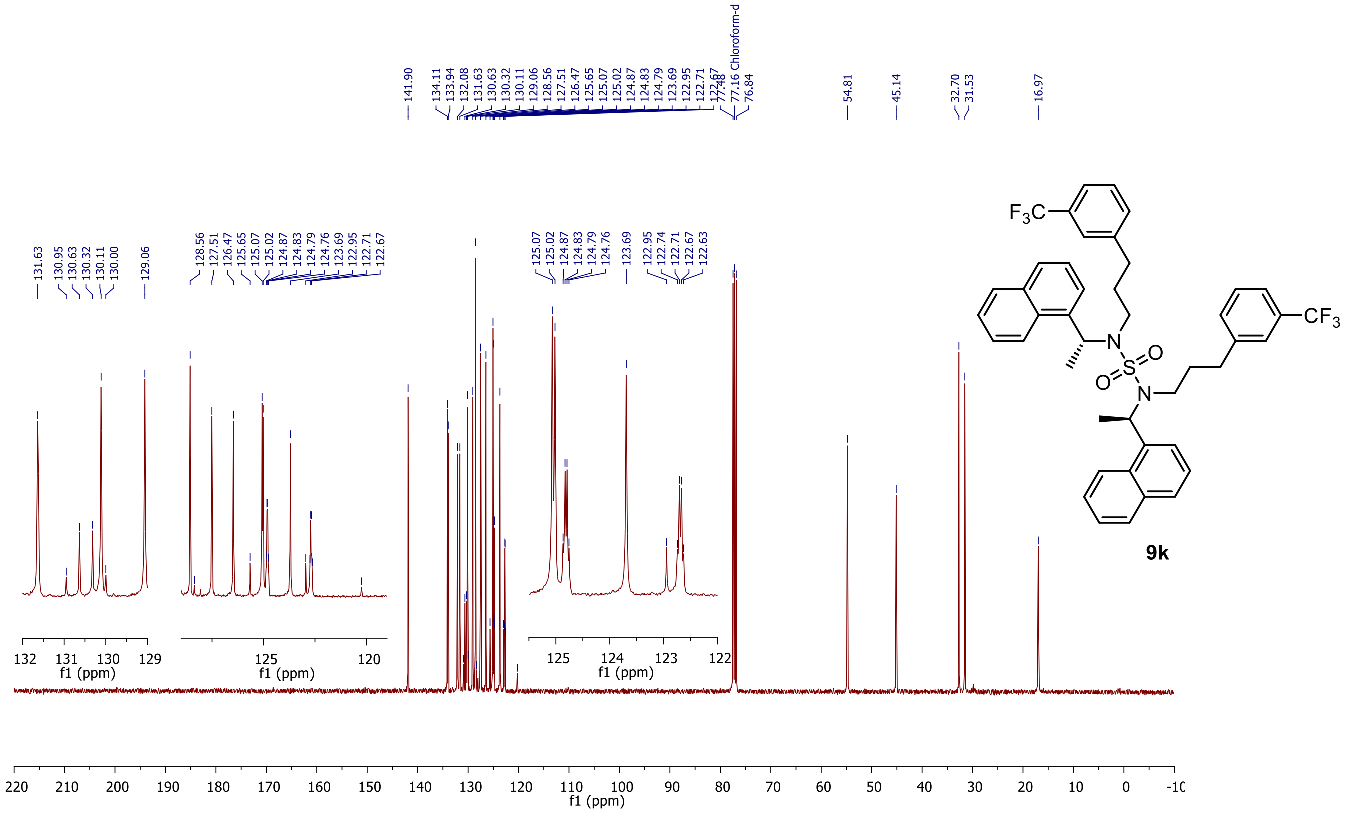

9k

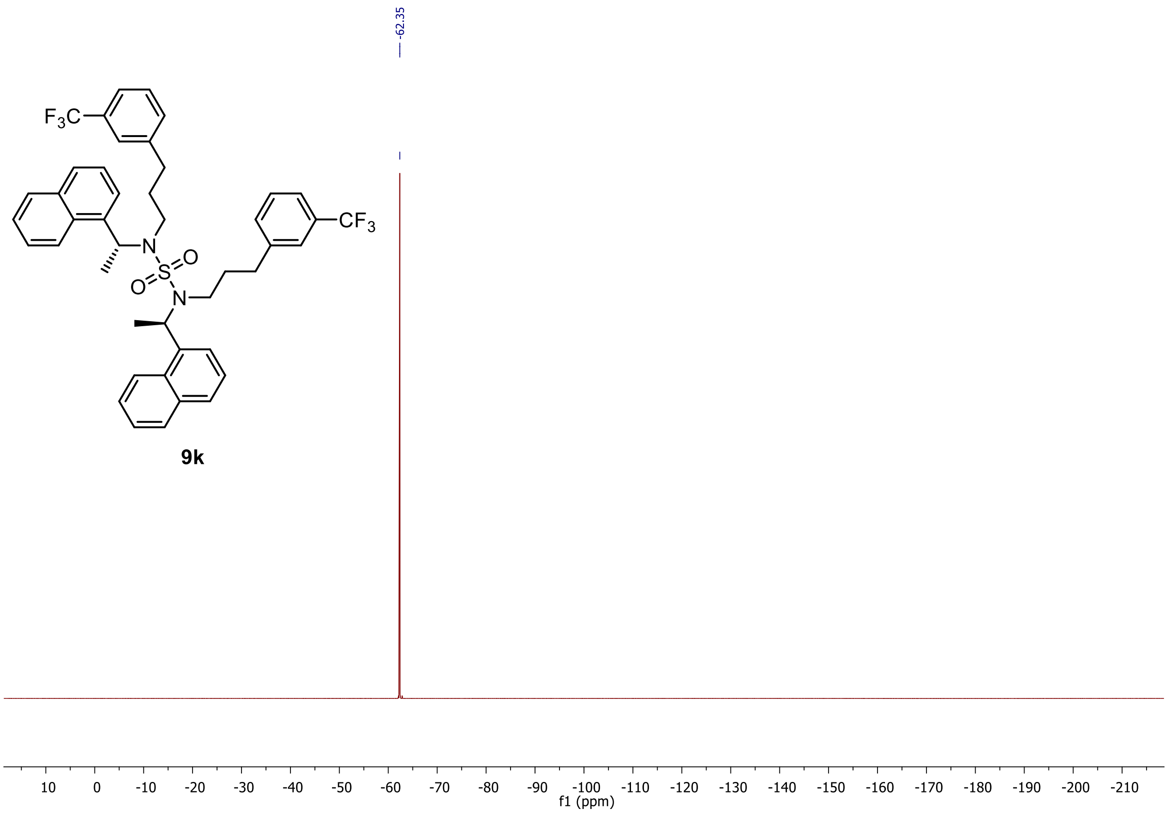

Figure S41: ^{1}H ($CDCl_3$, 400 MHz), ^{13}C{^{1}H} ($CDCl_3$, 101 MHz), and ^{19}F{^{1}H} ($CDCl_3$, 376 MHz) NMR Spectrum of **9k**.

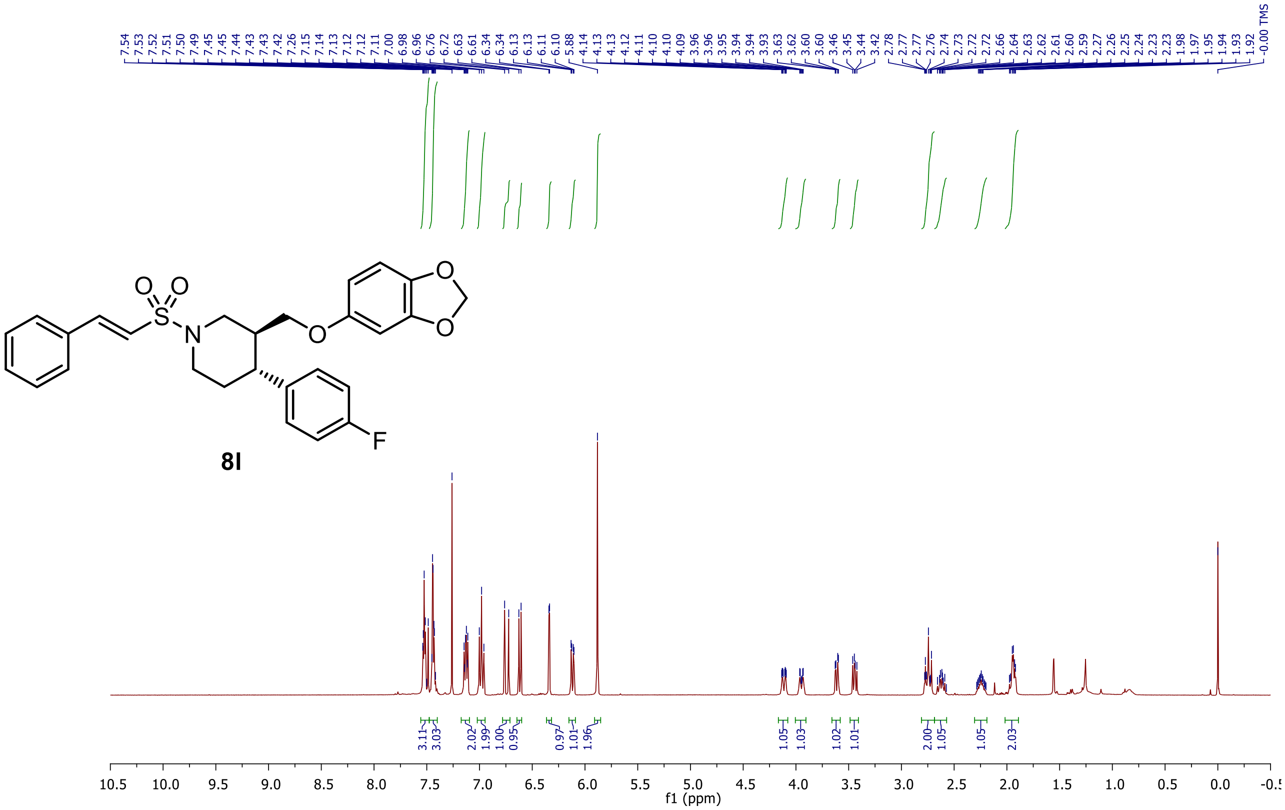
7.54 7.53 7.52 7.51 7.50 7.49 7.45 7.45 7.44 7.43 7.43 7.42 7.26 7.15 7.14 7.13 7.12 7.12 7.11 7.00 6.98 6.96 6.76 6.72 6.63 6.61 6.34 6.34 6.13 6.13 6.11 6.10 5.88 4.14 4.13 4.13 4.12 4.11 4.10 4.09 3.96 3.96 3.95 3.94 3.94 3.93 3.63 3.62 3.60 3.60 3.46 3.45 3.44 3.42 2.78 2.77 2.77 2.76 2.74 2.73 2.72 2.72 2.66 2.64 2.63 2.62 2.61 2.60 2.59 2.27 2.26 2.25 2.24 2.23 2.23 1.98 1.97 1.95 1.94 1.93 1.92
-0.00 TMS
8l
3.11 3.03 2.02 1.99 1.00 0.95 0.97 1.01 1.96 1.05 1.03 1.02 1.01 2.00 1.05 1.05 2.03
f1 (ppm)

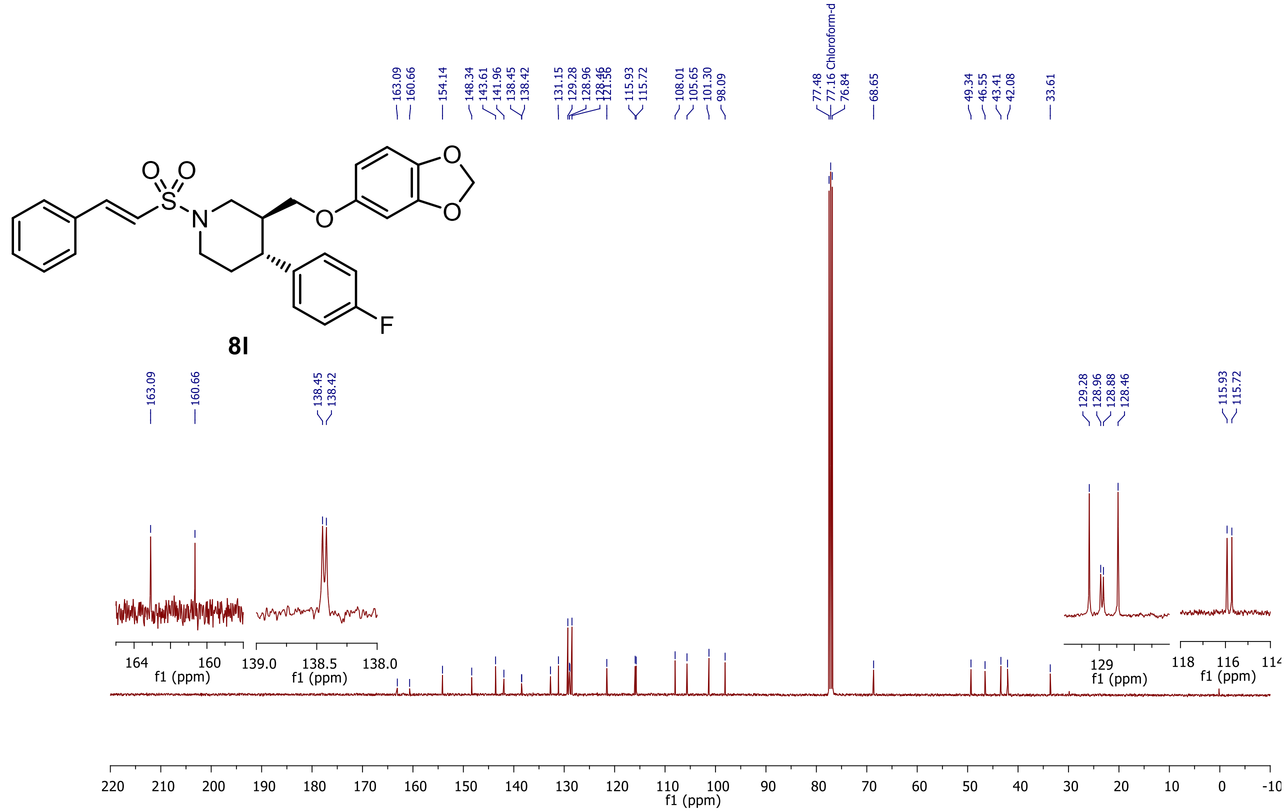
163.09 160.66 154.14 148.34 143.61 141.96 138.45 138.42 131.15 129.28 128.96 128.88 128.46 121.56 115.93 115.72 108.01 105.65 101.30 98.09
77.48 77.16 Chloroform-d 76.84 68.65 49.34 46.55 43.41 42.08 33.61
8l
f1 (ppm)

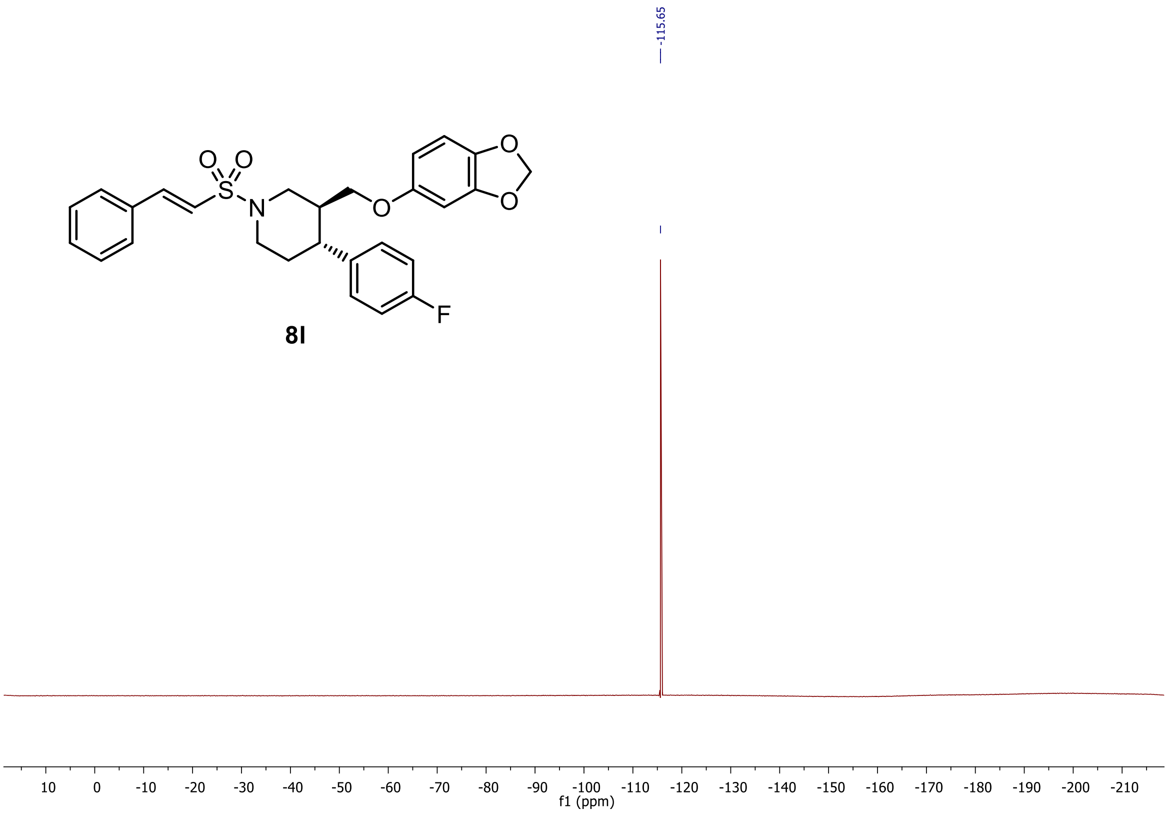

Figure S42: ^{1}H ($CDCl_3$, 400 MHz), $^{13}C\{^{1}H\}$ ($CDCl_3$, 101 MHz), and $^{19}F\{^{1}H\}$ ($CDCl_3$, 376 MHz) NMR Spectrum of **8l**.
